# Synthesis and Characterization of Ir-(κ^2^-NSi) Species Active toward the Solventless Hydrolysis of HSiMe(OSiMe_3_)_2_

**DOI:** 10.1021/acs.inorgchem.2c01973

**Published:** 2022-10-04

**Authors:** Alejandra Gómez-España, Pilar García-Orduña, Jefferson Guzmán, Israel Fernández, Francisco J. Fernández-Alvarez

**Affiliations:** † Departamento de Química Inorgánica-Instituto de Síntesis Química y Catálisis Homogénea (ISQCH), Universidad de Zaragoza−CSIC, Facultad de Ciencias, Zaragoza50009, Spain; ‡ Departamento de Química Orgánica I and Centro de Innovación en Química Avanzada, Facultad de Ciencias Químicas, Universidad Complutense de Madrid, Madrid28040, Spain; § Universidad Pedagógica Nacional Francisco Morazán-UPNFM, Tegucigalpa11101, Honduras

## Abstract

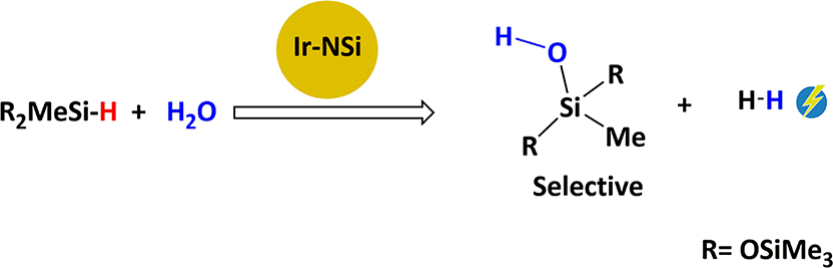

The reaction of [IrH(Cl)(κ^2^-NSi^tBu2^)(coe)] (**1**) with 1 equiv of PCy_3_ (or PH^t^Bu_2_) gives the species [IrH(Cl)(κ^2^-NSi^tBu2^)(L)] (L = PCy_3_, **2a**; PH^t^Bu_2_, **2b**), which reacts with 1 equiv of AgOTf to afford [IrH(OTf)(κ^2^-NSi^tBu2^)(L)] (L = PCy_3_, **3a** and PH^t^Bu_2_, **3b**). Complexes **2a**, **2b**, **3a**, and **3b** have been characterized by means of NMR spectroscopy and HR-MS. The solid-state structures of complexes **2a, 2b**, and **3a** have been determined by X-ray diffraction studies. The reversible coordination of water to **3a**, **3b**, and **4** to afford the corresponding adduct [IrH(OTf)(κ^2^-NSi^tBu2^)(L)(H_2_O)] (L = PCy_3_, **3a**-H_2_O; PH^t^Bu_2_, **3b**-H_2_O; coe, **4**-H_2_O) has been demonstrated spectroscopically by NMR studies. The structure of complexes **3b**-H_2_O and **4**-H_2_O have been determined by X-ray diffraction studies. Computational analyses of the interaction between neutral [NSi^tBu2^]^•^ and [Ir(*H*)L(X)]^•^ fragments in Ir-NSi^tBu2^ species confirm the electron-sharing nature of the Ir–Si bond and the significant role of electrostatics in the interaction between the transition metal fragment and the κ^2^-NSi^*t*Bu2^ ligand. The activity of Ir-(κ^2^-NSi^tBu2^) species as catalysts for the hydrolysis of HSiMe(OSiMe_3_)_2_ depends on the nature of the ancillary ligands. Thus, while the triflate derivatives are active, the related chloride species show no activity. The best catalytic performance has been obtained when using complexes **3a**, with triflate and PCy_3_ ligands, as a catalyst precursor, which allows the selective obtention of the corresponding silanol.

## Abstract 2

The Ir-(κ^2^-NSi^tBu2^) species catalyze the selective hydrolysis of HSiMe(OSiMe_3_)_2_ to give hydrogen and the corresponding silanol.

## Introduction

**Scheme 1:**
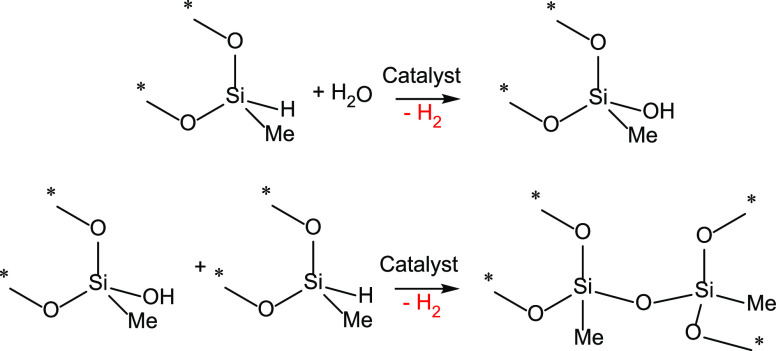


Silicones and siloxanes market reached a value of US$ 15.1 billion and US$ 8.8 billion in 2020, and despite the COVID-19 crisis, a continuous growth of ca. 7.5% is expected by 2026.^1^ In this context, the development of catalytic processes that use siloxanes as raw materials gains importance. Among them, hydrosiloxanes, which are obtained as side-products in the silicone industry, stand out because they have proven to constitute a cheap and easy-to-handle alternative to hydrosilanes as reducing and silylating agents.^2^ One of the simplest applications of hydrosiloxanes is their catalytic hydrolysis, which allows production of hydrogen and silanols. The latter are not stable, and under the reaction conditions, they typically react with the Si–H bond of another hydrosiloxane molecule to give higher-molecular-weight siloxanes and hydrogen (Scheme 1).^3^

**Scheme 2:**
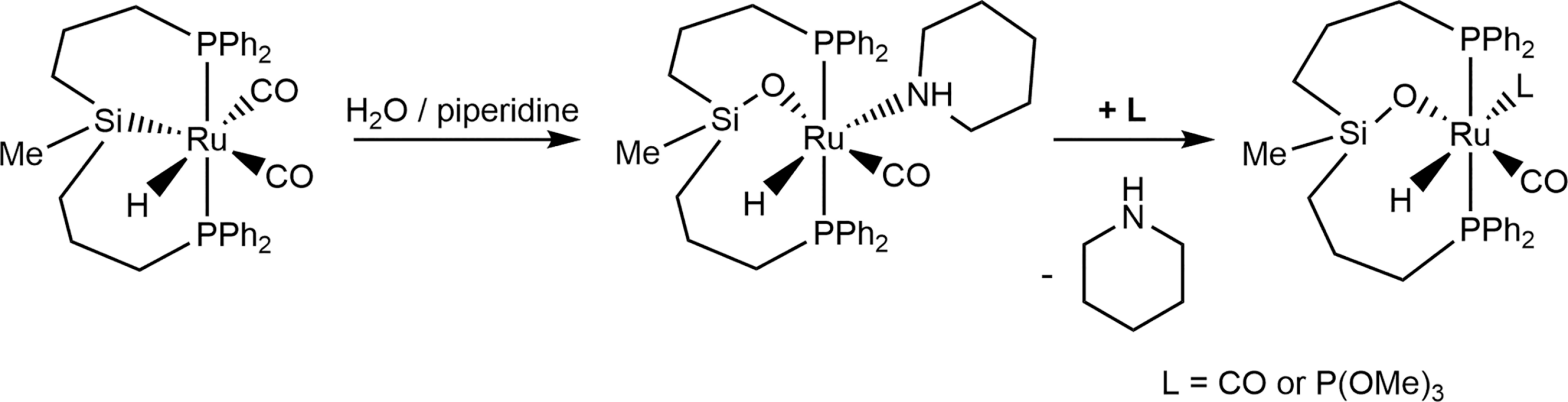


The development of catalysts for the selective hydrolysis of hydrosiloxanes to give hydrogen and the corresponding silanol is of great interest. However, while several examples of transition-metal-based homogeneous catalysts (Cr,^4^ Re,^5^ Fe,^6^ Ru,^7^ Rh,^8^ Ir,^9^ Cu,^10^ Ag,^11^ Au,^12^ and Zn^13^) effective for the hydrolysis of organosilanes have been reported, the catalytic hydrolysis of the Si–H bond in hydrosiloxanes remains challenging.^9c^ The first requirement that a hydrolysis catalyst must meet is to be stable in the presence of water, that is, the catalyst must not hydrolyze. This is a challenge when using transition metal catalysts with silyl-based ligands. In this regard, it should be noted that while metal-silicon bonds in late transition metal-silyl complexes are easily hydrolyzed, the reactivity with water of such bonds in transition metal compounds with κ^3^-ESiE-type (E = P^14^ and N^15^) pincer ligands is hindered and requires the presence of a base. Earlier examples of the reactivity of transition metal-(κ^3^-ESiE) complexes with water were reported by Stobart et al. in 2001.^16^ They described that the ruthenium(II) complex [RuH(PSiP^Ph^)(CO)_2_] (PSiP^Ph^ = *mer*-κ^3^-*P*,*Si*,*P*-Si(Me){(CH_2_)_3_PPh_2_}_2_) did not react with water at 100 °C, being necessary to heat its solutions in wet piperidine at 100 °C for 17 h to achieve the formation of the ruthenium-siloxyde complex [RuH(POP^Ph^)(CO)(piperidine)] (POP^Ph^ = *mer*-κ^3^-*P*,*O*,*P*-OSi(Me){(CH_2_)_3_PPh_2_}_2_), which decomposes upon isolation but reacts *in situ* with CO or P(OMe)_3_ to give the corresponding [RuH(POP^Ph^)(CO)(L)] (L = CO or P(OMe)_3_) species (Scheme 2).^16^

**Scheme 3:**
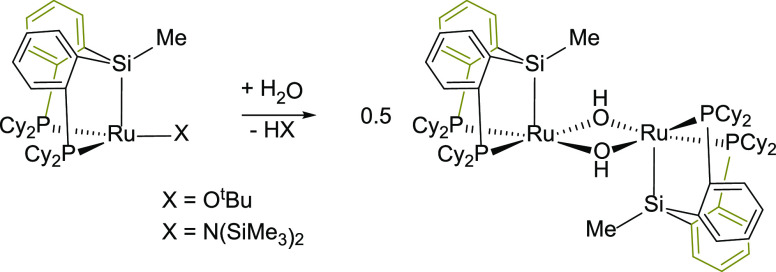


Some years later, Turculet et al. described that the addition of water to the ruthenium(II) complexes [Ru(X)*(*PSiP^Cy^)] (PSiP^Cy^ = *fac*-κ^3^-*P*,*Si*,*P*-Si(Me){PCy_2_(C_6_H_4_)}_2_; X = O^t^Bu and N(SiMe_3_)_2_) did not affect the Ru–Si bond but produced the protonolysis of the corresponding Ru–X bond to afford the dinuclear hydroxo-bridged species [{Ru*(*PSiP^Cy^)}(μ-OH)_2_] (Scheme 3).^17^

**Scheme 4:**
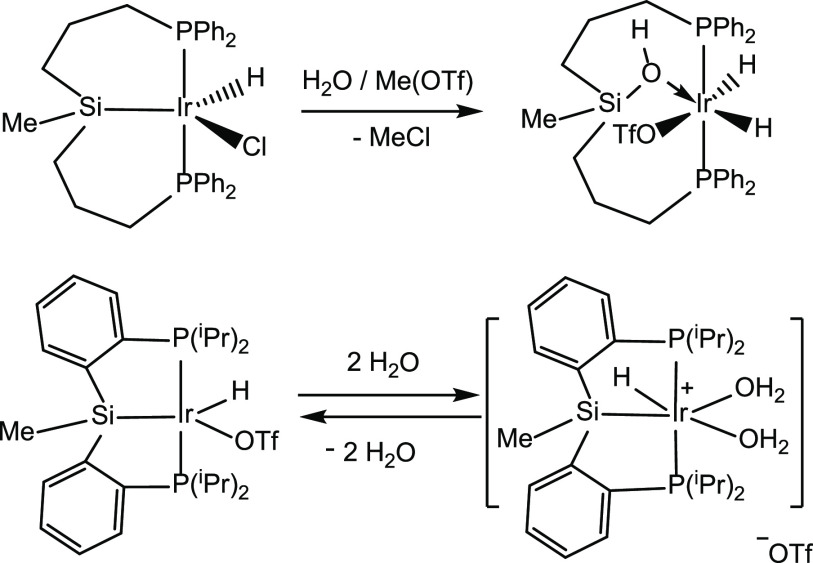


Sola et al. have studied the reactivity of Ir-(κ^3^-*P*,*Si*,*P*-PSiP) species with water. They have found that the reaction outcome strongly depends on the nature of the PSiP ligand. Thus, while the complex [IrH(Cl)*(*PSiP^Ph^)] reacts with MeOTf and water to give the complex [IrH(OTf)(POP^Ph^)],^18^ under the same reaction conditions, the Ir–Si bond in the related species [IrH(OTf)*(*PSiP^iPr^)] (PSiP^iPr^ = *mer*-κ^3^-*P*,*Si*,*P*-Si(Me){P(^i^Pr)_2_(C_6_H_4_)}_2_) is stable. Indeed, under these conditions, the reversible coordination of two molecules of water to afford the complex [IrH(PSiP^iPr^)(H_2_O)_2_][OTf] is observed (Scheme 4).^19^

**Scheme 5:**
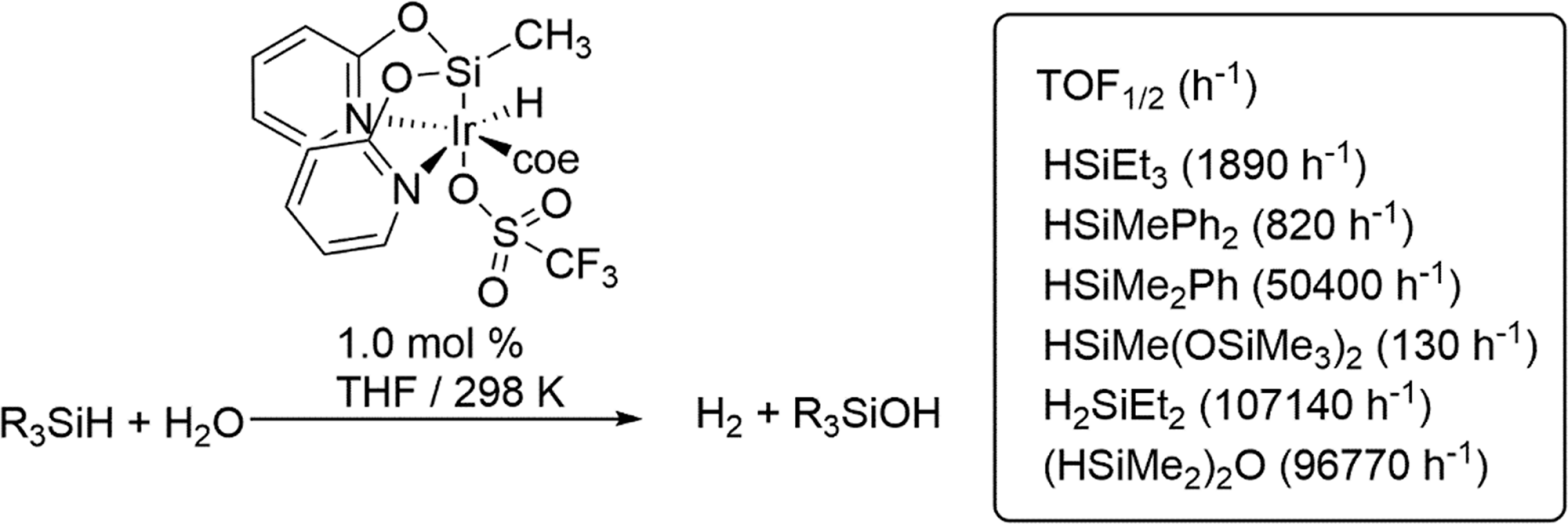


In contrast with κ^3^-PSiP ligands, which are commonly bonded to transition metals in a meridional (*mer*) coordination mode, monoanionic κ^3^-NSiN ligands prefer to coordinate to late transition metal complexes in a *fac*-κ^3^-(*N,Si,N*)-tridentate coordination mode, with the metal in a pseudo-octahedral geometry.^15^ Examples of the hydrolysis of the metal-silicon bond in metal-(κ^3^-*N*,*Si*,*N*-NSiN) species have not been reported so far. ^1^H and ^29^Si NMR studies on the catalytic generation of hydrogen by hydrolysis of hydrosilanes using the Ir(III) complex [Ir(H)(OTf)(NSiN)(coe)] (NSiN = *fac*-κ^3^-(*N,Si,N*)-bis(pyridine-2-yloxy)methylsilyl and coe = cyclooctene) as a catalyst precursor did not evidence the hydrolysis of the Ir–Si bond.^9c^ The low reactivity with water of this type of complexes becomes an advantage for their use as catalysts for the hydrolysis of hydrosilanes. The performance of Ir-{*fac*-κ^3^-(*N,Si,N*)-NSiN} species as catalysts for the hydrolysis of hydrosilanes is strongly dependent on the reaction solvent, and the best activities were obtained in THF.^9c^ The highest activities were reported for Et_2_SiH_2_ (TOF_1/2_ = 107,140 h^–1^) and (Me_2_HSi)_2_O (TOF_1/2_ = 96,770 h^–1^), whereas the lowest one was found for HSiMe(OSiMe_3_)_2_ (TOF_1/2_ = 130 h^–1^).^9c^ The activity trend shown in Scheme 5 has been attributed to the steric hindrance around the Si–H bond.

These previous findings encouraged us to develop less hindered iridium catalysts with bidentate instead of tridentate ligands. Indeed, iridium complexes of the type Ir-(κ^2^-NSi^Me2^)_2_ (NSi^Me2^ = 4-methylpyridin-2-yloxy-dimethylsilyl) are more active catalysts for the reduction of CO_2_ with silanes than the corresponding Ir-{*fac*-κ^3^-(*N,Si,N*)-NSiN} species.^20^ However, the Ir–Si bond in Ir-(κ^2^-NSi^Me2^)_2_ species is not stable under protic conditions.^21^ Therefore, we decided to protect the Ir–Si bond with two bulky tertbutyl substituents at the silicon atom of the κ^2^-NSi^tBu2^ ligand.^22^

Herein, as a continuation of our previous studies on the chemistry of iridium species with bidentate pyridine-yloxy-silyl ligands,^20−22^ we describe the results of our investigations about the potential of Ir(III) species with the monoanionic bidentate ligand 4-methylpyridin-2-yloxy-ditertbutylsilyl (κ^2^-NSi^tBu2^)^22^ as catalysts for the solventless hydrolysis of hydrosiloxanes. In addition, the bonding situation in the novel species was analyzed in detail by means of density functional theory (DFT) calculations.

## Results and Discussion

### Ir-(κ^2^-NSi^tBu2^)(PR_3_) Complexes

**Scheme 6:**
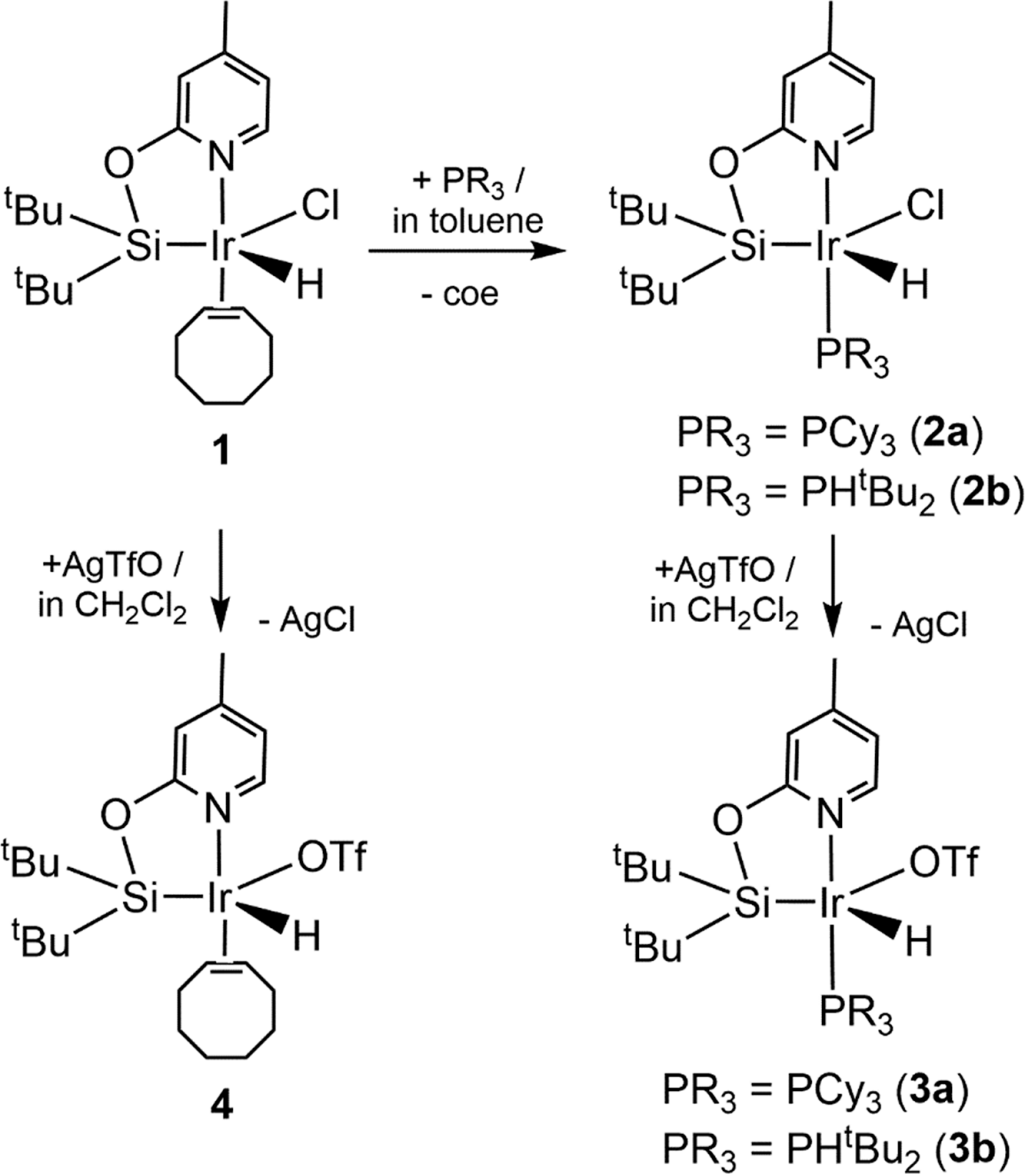


The iridium(III) complex [IrH(Cl)(κ^2^-NSi^tBu2^)(coe)] (**1**)^22^ reacts with 1 equiv of PCy_3_ or PH(^t^Bu_2_) to quantitatively give the corresponding phosphane adduct [IrH(Cl)(κ^2^-NSi^tBu2^)(L)] (L = PCy_3_, **2a**; PH(^t^Bu_2_), **2b**). ^1^H NMR (C_6_D_6_) studies evidenced that these reactions are quantitative and only the resonances due to the corresponding species **2a** (or **2b**) and to free coe are observed. Complexes **2a** and **2b** were isolated as yellow solids in 93 and 77% yield, respectively (Scheme 6). The best methodology for the preparation of **3a** and **3b** was found to be the reaction of light-protected solutions of **2a** or **2b** in CH_2_Cl_2_ with 1 equiv of AgOTf; this procedure leads to species **3a** and **3b**, which were isolated as off-white solids in 82 and 79% yields, respectively (Scheme 6). ^1^H NMR studies of the reaction of [IrH(OTf)(κ^2^-NSi^tBu2^)(coe)] (**4**)^22^ with PR_3_ in C_6_D_6_ show the formation of the corresponding [IrH(X)(κ^2^-NSi^tBu2^)(L)] (L = PCy_3_, **3a** or PH(^t^Bu_2_), **3b**) species; however, these reactions are not selective and mixtures of species, some of them with two phosphine ligands, are observed.

Complexes **2a**, **2b**, **3a**, and **3b** have been characterized by means of multinuclear NMR spectroscopy, IR, and HR-MS (Figures S1–S26). The ^1^H NMR spectra of species **2a** and **3a** in C_6_D_6_ show the Ir–H resonance as a doublet at δ −22.39 ppm (^2^*J_P–H_* ≈ 16 Hz; **2a**) and δ −29.21 (^2^*J_P–H_* ≈ 17 Hz; **3a**). The most characteristic resonances in the ^1^H NMR spectra of **2b** and **3b** in C_6_D_6_ are a double-doublet resonance centered at δ −23.82 (^2^*J_P–H_* ≈ 16 Hz, ^3^*J_H–H_* ≈ 2.9 Hz; **2b**) and δ −29.31 (^1^*J_P–H_* ≈ 17 Hz, ^3^*J_H–H_* ≈ 2.4 Hz; **3b**) and a double-doublet centered at δ 4.98 (^1^*J_P–H_* ≈ 346 Hz, ^3^*J_H–H_* ≈ 2.9 Hz; **2b**) and δ 4.85 (^1^*J_P–H_* ≈ 355 Hz, ^3^*J_H–H_* ≈ 2.4 Hz; **3b**), which correspond to the Ir–H and P–H protons, respectively. The Ir–H resonances in species **2** and **3** are high-field shifted in comparison with those observed for the parent complexes [IrH(X)(κ^2^-NSi^tBu2^)(coe)] (X = Cl, **1**; OTf, **4**), which appear at δ −20.68 (**1**) and −27.46 (**4**). The ^31^P{^1^H} NMR spectra of these complexes show a singlet at δ 11.8 ppm (**2a**), 26.9 (**2b**), 10.3 (**3a**), and 27.9 (**3b**). Their ^29^Si{^1^H} NMR spectra at 298 K show a singlet resonance at δ 44.1 (**2a**), 43.3 (**2b**), 44.5 (**3a**), and 45.4 (**3b**). These values compare well with those observed in the ^29^Si{^1^H} NMR spectra of **1** (δ 41.9) and **4** (δ 45.8). The IR spectra of species **2** and **3** show a clear dependence between the value of the signal corresponding to the Ir–H bond and the ancillary ligands that appear at 2244 cm^–1^ (**2a**) and 2266 cm^–1^ (**2b**) for the chloride derivatives and at 2309 cm^–1^ (**3a**) and 2338 cm^–1^ (**3b**) for the triflate derivatives.

The structures proposed for **2a**, **2b**, and **3a** in Scheme 6 have been confirmed by single-crystal X-ray diffraction. Selected geometrical parameters, describing the metal coordination sphere, are reported in Table 1.

**Table 1:** Selected Bond Lengths (Å) and Angles (°) for Complexes **2a**, **2b**, and **3a**

	**2a**	**2b**	**3a**		**2a**	**2b**	**3a**
Ir–X^a^	2.4015(3)	2.3902(4)	2.2355(14)	X^a^–Ir–H	165.1(7)	98.0	177.0(11)
Ir–P	2.2782(3)	2.2610(4)	2.2873(5)	P–Ir–Si	113.359(12)	103.357(14)	112.190(17)
Ir–Si	2.2792(3)	2.2814(4)	2.2835(5)	P–Ir–N	163.19(3)	171.17(3)	167.54(4)
Ir–N	2.0967(10)	2.1212(12)	2.0936(15)	P–Ir–H	85.9(7)	93.0	89.8(11)
Ir–H	1.473(19)	1.60	1.550(17)	Si–Ir–N	79.42(3)	79.70(3)	79.76(4)
X^a^–Ir–P	93.034(11)	93.507(14)	91.19(4)	Si–Ir–H	71.1(7)	121.00	74.4(11)
X^a^–Ir–Si	122.469(12)	135.987(15)	107.83(4)	N–Ir–H	88.4(7)	78.0	90.2(11)
X^a^–Ir–N	88.53(3)	89.77(4)	88.24(6)				

a X = Cl in complexes **2a** and **2b**; O(2) in complex **3a**.

**Figure 1:**
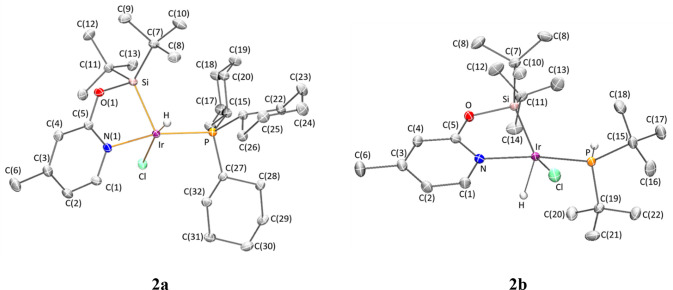
Molecular structures of complexes **2a** and **2b**. Hydrogen atoms (except hydrides) have been omitted for clarity.

**Figure 2:**
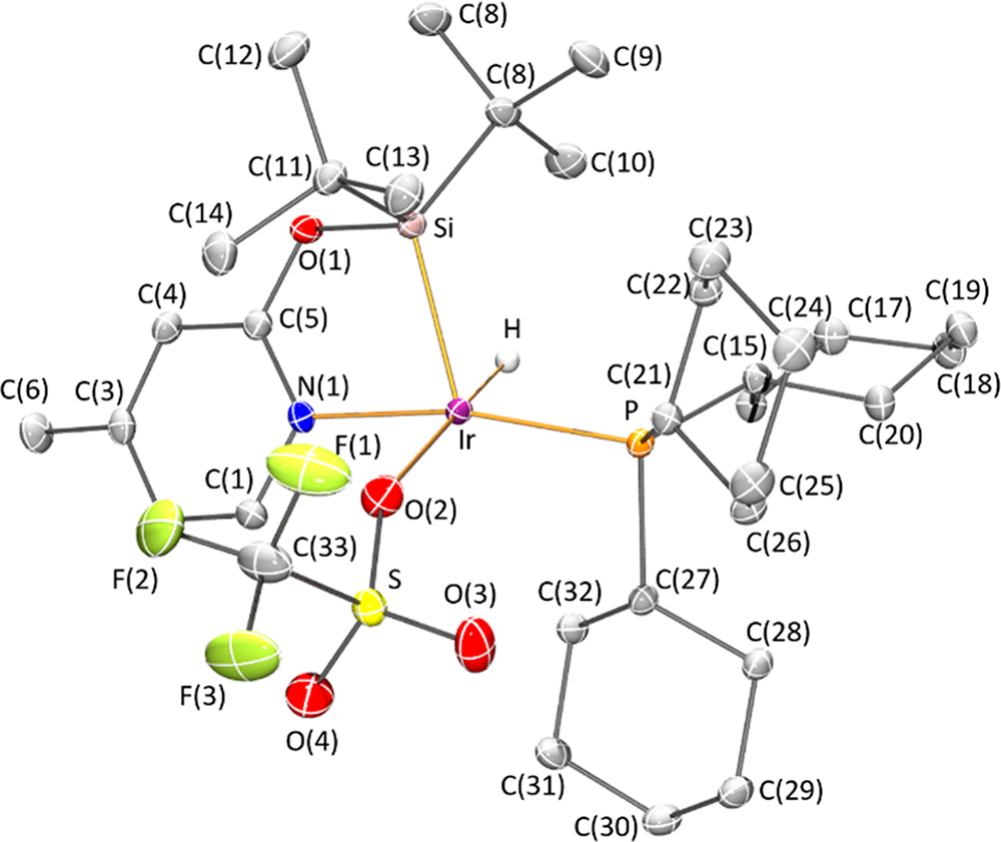
Molecular structure of complex **3a**. Hydrogen atoms (except hydride) have been omitted for clarity.

As illustrated in Figures 1 and 2 and pointed out in Table 1, the geometry of the metal coordination sphere of pentacoordinate complexes **2a**, **2b**, and **3a** depends on the phosphine ligand. On one hand, in complexes **2a** and **3a**, with bulkier PCy_3_, the coordination of the κ^2^-NSi^tBu2^ ligand through the N and Si atoms, together with that of the phosphorous, the hydride and a chloride (**2a**) or an oxygen atom of a triflate ligand (**3a**) leads to a square pyramidal geometry around the iridium atom, with a silicon atom in the apical position and the chloride (**2a**) or the oxygen atom of the triflate ligand (**3a**) located *trans* to the hydride. Geometry indexes for these compounds are τ =0.03 and 0.19 for **2a** and **3a**, respectively (τ = 0 and τ = 1 correspond to ideal square pyramidal and trigonal bipyramidal).^23^ Interestingly, in both crystal structures, a hydrogen atom of a cyclohexyl ring (H32A) directly points toward the lone pair of the metal.

On the other hand, complex **2b** exhibits an intermediate situation between a square pyramidal geometry and a distorted trigonal bipyramidal iridium atom with nitrogen and the phosphorous atoms at apical positions and equatorial sites occupied by silicon, chloro, and hydrogen atoms, with a geometry index of τ = 0.58.

The Ir–Si (2.2792(3) Å, **2a** and 2.2814(4) Å, **2b**) and the Ir–Cl (2.4015(3) Å, **2a** and 2.3902(4) Å, **2b**) bond lengths compare well with the values 2.2853(6) Å and 2.3950(6) Å reported for **1**,^22^ respectively. Geometrical parameters of the coordination of the bidentate (κ^2^-NSi^tBu2^) fragment and the phosphano ligands to the metal in **3a** nicely agree with those observed for complexes **2a** and **2b**.

### Addition of Water to [IrH(X)(κ^2^-NSi^tBu2^)(L)] (X = Cl and OTf; L = Coe, PCy_3_, and PH^t^Bu_2_)

**Figure 3:**
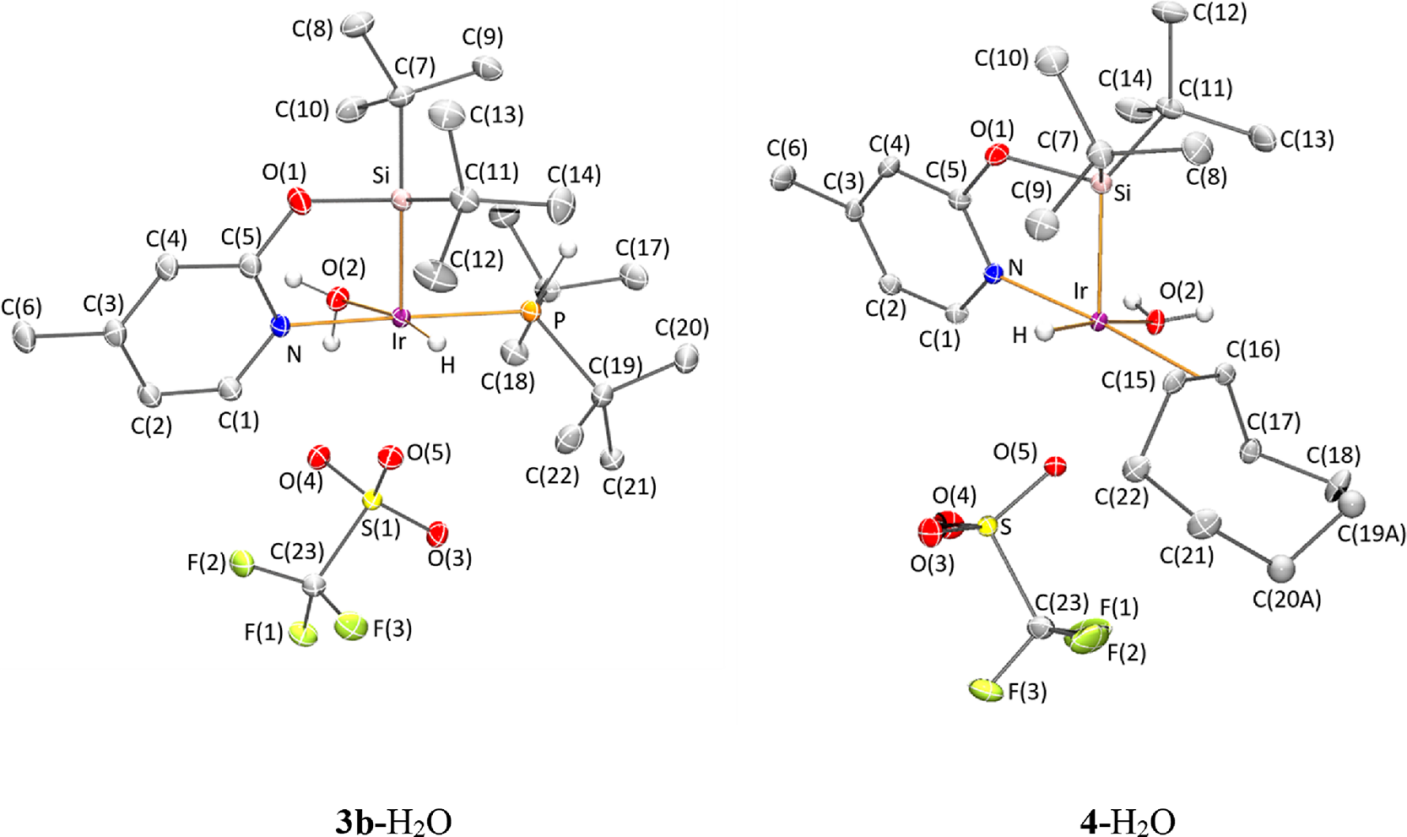
Molecular structure of complexes **3b-**H_2_O and **4-**H_2_O.

While the ^1^H NMR spectra of solutions of the Ir-chloride derivatives **1**, **2a**, and **2b** in CD_2_Cl_2_ (298 K) do not show detectable changes after the addition of water (10 μL), those of the related Ir-triflate complexes **4**, **3a**, and **3b** evidenced a shift in most of the resonances, which is significant for the signals due to the Ir–H moiety. In particular, in the ^1^H NMR (CD_2_Cl_2_) spectra, they appear shifted downfield from δ −27.39 to −25.82 ppm (**4**), from δ −29.75 to −29.08 (**3a**), and from δ −29.71 to −28.98 (**3b**) (Figures S27–S29). Moreover, the serendipitous obtention of single crystals from wet C_6_D_6_ and pentane solutions of **4** and **3b**, respectively, allowed us to determine the solid-state structure of the water adducts and **3b**-H_2_O and **4**-H_2_O (Figure 3).

In complexes **3b**-H_2_O and **4**-H_2_O, the metal atom exhibits a distorted pseudo-octahedral geometry with nitrogen, oxygen atom of water, hydride, and phosphorous (**3b**-H_2_O) or the olefinic bond of coe ligand (**4**-H_2_O) in the equatorial plane, while apical positions are fulfilled by silicon and an oxygen atom of the triflate ligand. The Ir–Si bonds (2.2835(5), 2.2876(4), and 2.2915(6) Å in **3a**, **3b**-H_2_O, and **4**-H_2_O, respectively) (Table 2) are comparable to those found in complexes **1**, **2a**, and **2b**, while the trans-located Ir···O bonds in **3b**-H_2_O and **4**-H_2_O are found to be significantly elongated. These Ir–O_triflate_ bond lengths in **3b**-H_2_O and **4**-H_2_O (2.4921(11) and 2.6073(1) Å) are longer than those found in complex **3a** (2.2355(14) Å) where the *trans* effect of the hydride is evident, but they are also longer than those reported in related complexes where oxygen is *trans* to silicon, as in [Ir(μ-OTf)(κ^2^-NSi^Me2^)_2_]_2_ dinuclear complex (2.3653(12) and 2.4331(13) Å)^20b^ or in species [Ir(CF_3_CO_2_)(κ^2^-NSi^Me2^)_2_] (2.363(3) and 2.418(3) Å).^20a^

**Table 2:** Selected Bond Lengths (Å) and Angles (°) for Complexes **3b**-H_2_O and **4**-H_2_O

	**3b**-H_2_O	**4**-H_2_O		**3b**-H_2_O	**4**-H_2_O
Ir–X^a^	2.2795(4)	2.0709(15)	X^a^–Ir–H	84.8(9)	87.5(10)
Ir–Si	2.2876(4)	2.2915(6)	Si–Ir–O(2)	112.31(3)	109.60(4)
Ir–O(2)	2.2789(11)	2.2218(15)	Si–Ir···O(5)	164.83(3)	165.3(1)
Ir···O(5)	2.4921(11)	2.6073(1)	Si–Ir–N	80.96(4)	80.82(5)
Ir–N	2.1167(12)	2.0797(17)	Si–Ir–H	74.8(9)	78.6(10)
Ir–H	1.45(2)	1.464(16)	O(2)–Ir···O(5)	78.68(4)	77.10(6)
X^a^–Ir–Si	97.918(15)	100.47(7)	O(2)–Ir–N	84.37(5)	85.74(6)
X^a^–Ir–O(2)	99.46(3)	99.11(7)	O(2)–Ir–H	170.9(9)	168.1(10)
X^a^–Ir···O(5)	90.18(3)	91.03(7)	O(5)···Ir–N	90.11(4)	86.84(6)
X^a^–Ir–N	176.14(3)	174.15(6)	O(5)···Ir–H	93.3(9)	93.0(10)

a X = P in complex **3b**-H_2_O; centroid of the olefinic bond in complex **4**-H_2_O.

**Figure 4:**
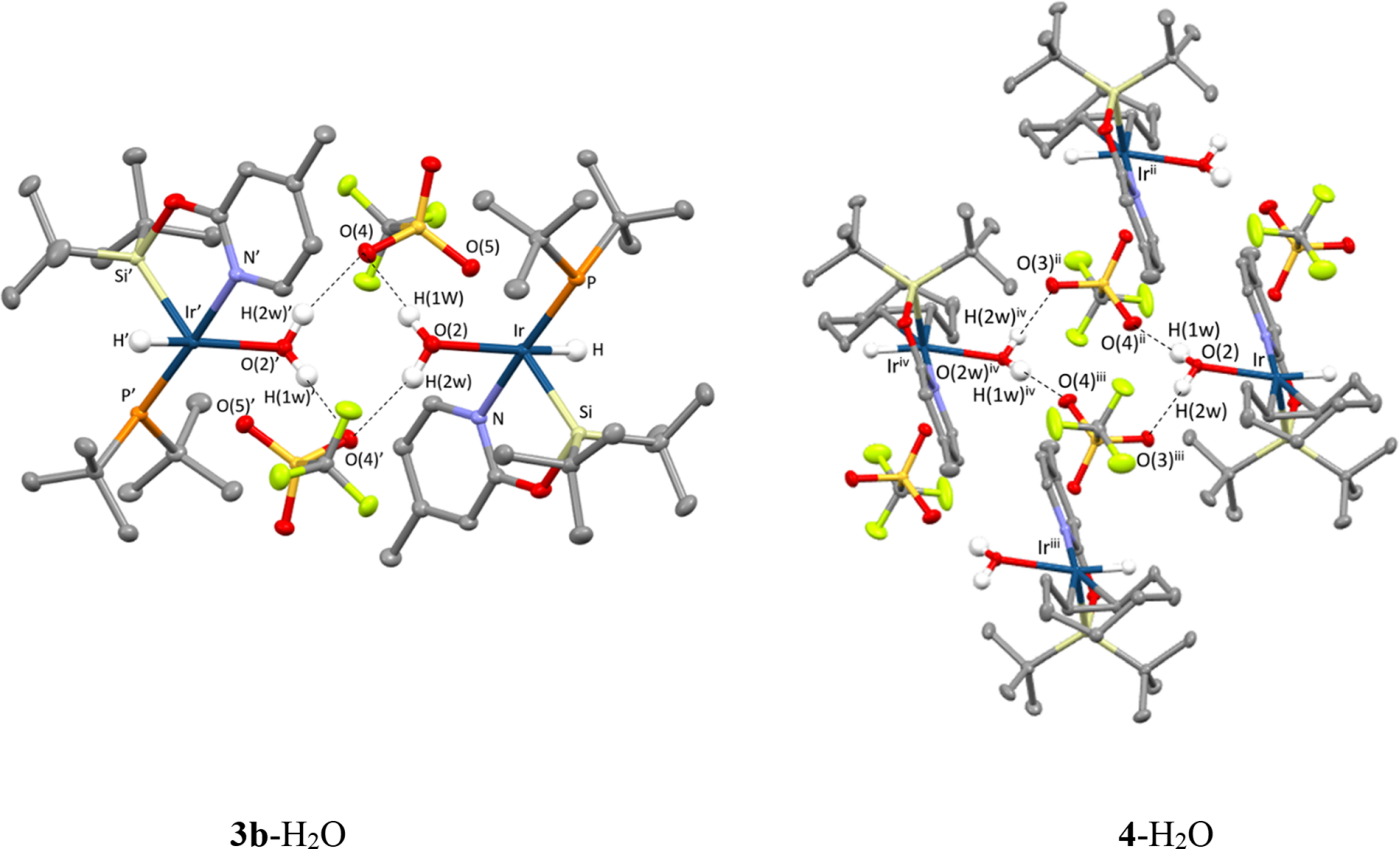
Hydrogen bond interactions in **3b**-H_2_O and **4**-H_2_O. Symmetry operations: (i)1 – *x*, 1 – *y*, −*z*; (ii) 1–*x*, −*y*, 2 – *z*; (iii) 1 – *x*, *y*, *z*; (iv) −*x*, −*y*, 2 – *z*.

The coordinated water molecule in **3b**-H_2_O and **4**-H_2_O is placed *trans* to the hydride ligand. Both hydrogen atoms of the water molecule establish hydrogen bond interactions with the oxygen atoms of the triflate ligands, whose geometrical parameters are reported in Table 3. In the **3b**-H_2_O crystal, these interactions form a symmetric *R*_2_^2^(8) pattern,^24^ involving two **3b**-H_2_O molecules, through an intramolecular (O(2)-H(1 W)···O(4)) and an intermolecular ((O(2)-H(2 W)···O(3)′)) contacts. A different relative orientation of coordinated water and triflate ligand in the solid-state structure of **4**-H_2_O leads to the formation of intermolecular O–H···O_triflate_ interactions, forming an *R*_2_^4^(8) pattern, as depicted in Figure 4.

**Table 3:** Geometrical Parameters (Å, °) of Hydrogen Bond Interactions^a^

compound	D–H···A	D–H	H···A	D···A	D–H···A
**3b**-H_2_O	O(2)–H(1 W)···O(4)	0.86(2)	2.07(2)	2.9130(18)	167(2)
**3b**-H_2_O	O(2)–H(2 W)···O(4)^i^	0.84(3)	1.99(3)	2.7987(18)	162(3)
**4**-H_2_O	O(2)–H(1 W)···O(4)^ii^	0.82(4)	1.92(3)	2.733(2)	174(4)
**4**-H_2_O	O(2)–H(2 W)···O(3)^iii^	0.82(3)	1.98(3)	2.796(2)	176(3)

a Symmetry operations: (i): 1 – *x*, 1 – *y*, −*z*; (ii) 1 – *x*, −*y*, 2 – *z*; (iii) 1 – *x*, *y*, *z*.

**Scheme 7:**
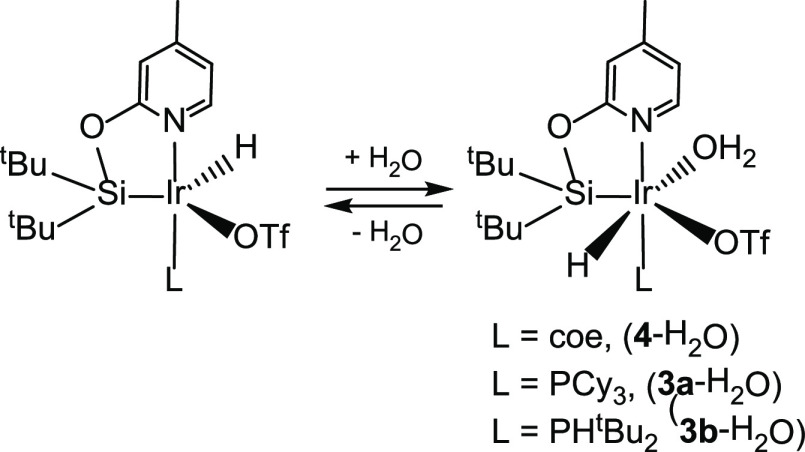


The above-described results encouraged us to further study the reactivity of the Ir-triflate derivatives **3a**, **3b**, and **4** with water. ^1^H NMR spectra of CD_2_Cl_2_ solutions of complexes **3a**-H_2_O, **3b**-H_2_O, and **4**-H_2_O are temperature-dependent (Figures S30–S35). In all the cases, a reversible shift of the signals was observed in the ^1^H NMR spectra with decreasing temperatures, which agrees with the water coordination/dissociation equilibrium depicted in Scheme 7. Species **4**-H_2_O and **3b**-H_2_O show a fast equilibrium in the NMR time scale between 298 and 193 K. However, in the case of **3a**-H_2_O, the ^1^H NMR variable temperature (VT NMR) studies in CD_2_Cl_2_ show the coalescence at 273 K (*T*_c_), below *T*_c_, and an equilibrium between two unequally populated species is observed (Figure S31).

**Figure 5:**
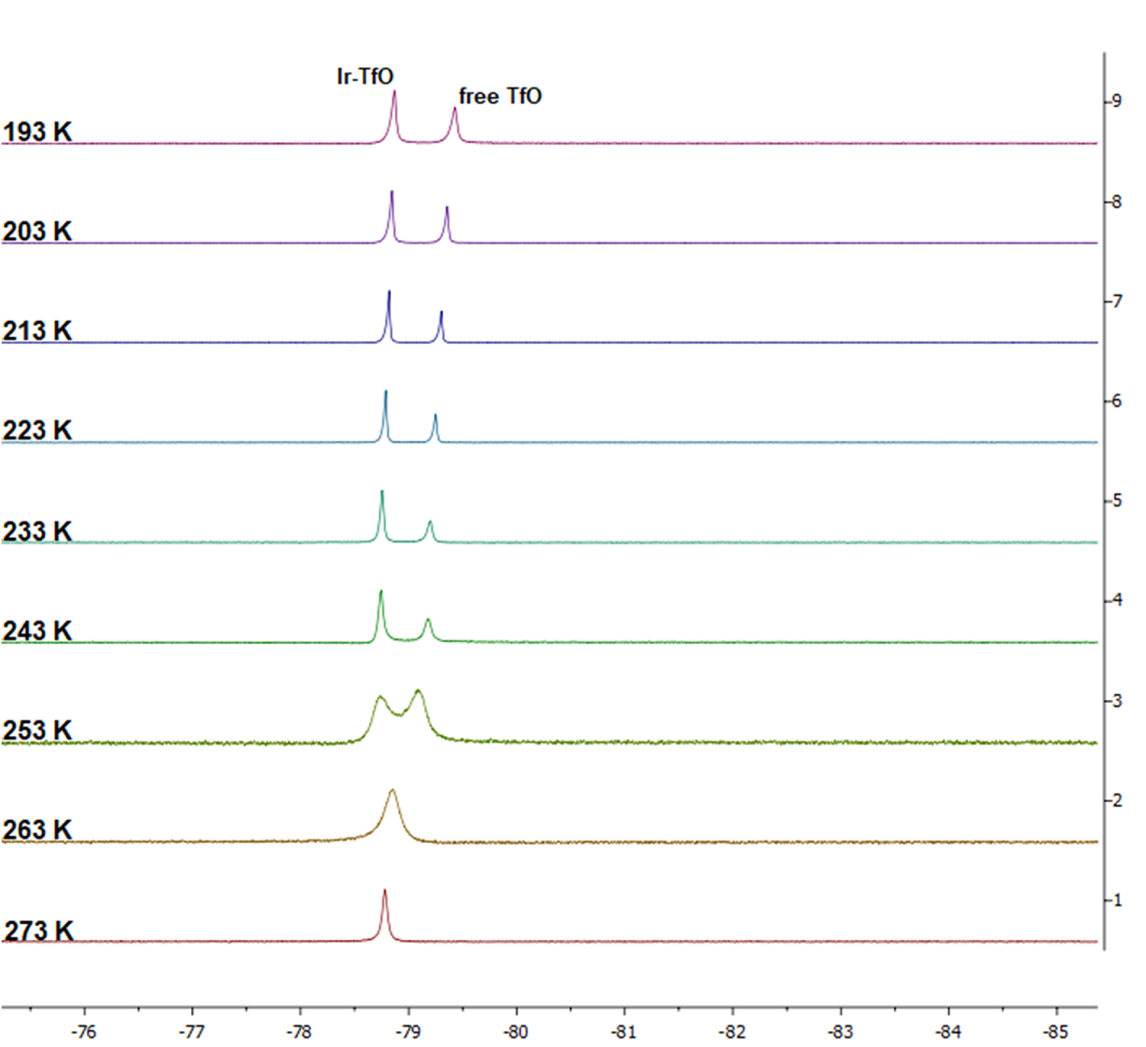
VT ^19^F{^1^H} NMR spectra of **3a** ⇆ **3a**-H_2_O from 273 to 193 K.

The ^19^F{^1^H} NMR spectra of **3a** ⇆ **3a**-H_2_O, **3b** ⇆ **3b**-H_2_O, and **4** ⇆ **4**-H_2_O solutions in CD_2_Cl_2_ are also temperature-dependent. The ^19^F{^1^H} NMR spectra of **3b** ⇆ **3b**-H_2_O and **4** ⇆ **4**-H_2_O show, in both cases, only one resonance at δ −78.6 (298 K) to −79.0 (193 K); these values are close to the value reported for free triflate in CD_2_Cl_2_ at 298 K (δ −79.0)^19^ and agree with the long Ir–O_triflate_ bond lengths found in **3b**-H_2_O and **4**-H_2_O. The ^19^F{^1^H} NMR spectra of **3a** ⇆ **3a**-H_2_O (CD_2_Cl_2_) show a resonance at δ −78.6 at 298 K that broadens as the temperature decreases; below 253 K, the signal splits into two clearly different peaks at δ −78.7 and −79.2 ppm (ratio: 1.00:0.58), corresponding to Ir–OTf and free ^–^OTf, respectively (Figure 5). Cooling the sample to 193 K produces not only a slight high-field shifting of the two signals to δ −78.9 and −79.4 ppm, respectively but also a population change (ratio: 1.00:0.77). These results suggest that in the case of complex **3a**-H_2_O, probably due to the high steric demand of the PCy_3_ ligand, a triflate ligand dissociation/coordination equilibrium also takes place.

The chemical shift variations with temperature observed in the ^1^H, ^19^F, and ^29^Si NMR spectra of CD_2_Cl_2_ solutions of species **4**-H_2_O and **3b**-H_2_O are not significant, and consequently, these data were not suitable to study the equilibria **4** ⇆ **4**-H_2_O and **3b** ⇆ **3b**-H_2_O. Fortunately, the ^31^P{^1^H} NMR spectra of **3b**-H_2_O (CD_2_Cl_2_) show a greater chemical shift variation with temperature, and therefore, these data were used to calculate the Δ*H*° (−3.45 ± 0.35 kcal mol^–1^), Δ*S*° (−1.47 ± 0.15 cal mol^–1^ K^–1^), and Δ*G*°_298_ (−3.01 ± 0.30 kcal mol^–1^) values for the **3b** ⇆ **3b**-H_2_O equilibrium, accordingly to the fast exchange equations.^25,26^

The Δ*G*^‡^ values for the interconversion **3a** ⇆ **3a**-H_2_O have been calculated using the Ir–H resonances in the ^1^H NMR spectra and the equations for the interconversion between two unequally populated species Δ*G*^‡^_A_ = 4.57*T*_c_{10.62 + log[*X*/2π(1 – Δ*P*)] + log(*T*_c_/Δν)} and Δ*G*^‡^_B_ = 4.57*T*_c_{10.62 + log[*X*/2π(1 + Δ*P*)] + log(*T*_c_/Δν)},^27^ where *T*_c_ is the coalescence temperature (273 K). The difference of populations between **3a** and **3a**-H_2_O (Δ*P*) at 193 K is 0.16, and the value of Δν, which is the difference between the two studied resonances in Hz, at 193 K is 729 Hz. The parameter *X* can be obtained from the equation Δ*P* = [(*X*^2^ – 2)/3]^3/2^ × 1/*X*, and for Δ*P* = 0.16 is 1.8218.^28^ Thereby, the Δ*G*^‡^_A_ and Δ*G*^‡^_B_ values were calculated as 12.6 and 12.4 kcal mol^–1^, respectively.

These results show that the coordination of the water molecule to the iridium atom in complexes **3a**, **3b**, and **4** to give the corresponding adduct depends on the temperature, being preferred at low temperatures. Accordingly, none of the aquo complexes **3a**-H_2_O, **3b**-H_2_O, or **4**-H_2_O could be isolated at a preparative scale and even using an excess of water; the starting products are obtained after the workup.

### Computational Studies on the Ir–Si and Ir–OTf Bonds

The remarkable stability of the Ir–Si bond in complexes **1**–**3** prompted us to investigate the nature of the Ir–Si bond in detail by means of computational tools at the relativistic and dispersion-corrected ZORA-BP86-D3/TZ2P//BP86-D3/def2-SVP level (see computational details in the Supporting Information). To this end, we applied state-of-the-art methods based on the natural bond orbital (NBO) and energy decomposition analysis-natural orbital for chemical valence (EDA-NOCV) methods on the representative **1**, **3b**, and **4** complexes as well as on their corresponding H_2_O complexes.

**Figure 6:**
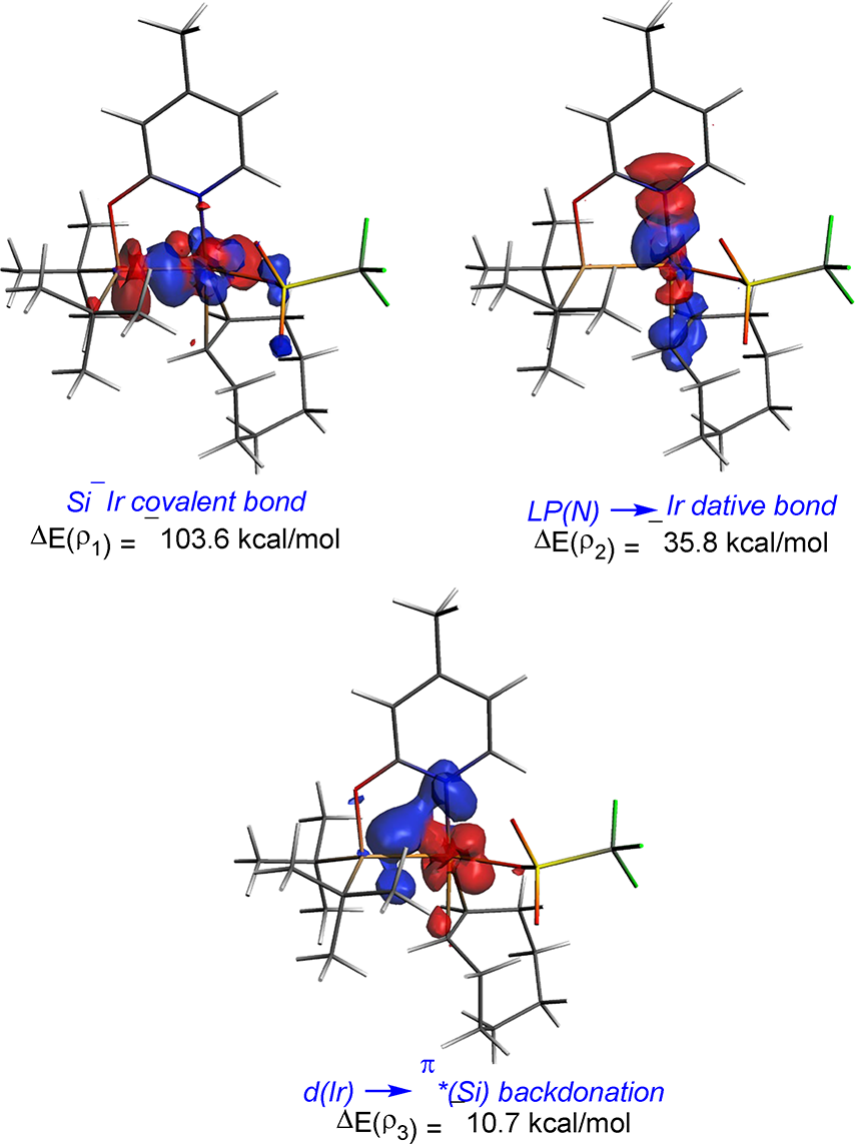
NOCV-deformation densities and associated stabilization energies computed for complex **4**. The charge flow takes place in the direction red → blue.

Similar to the slightly related [Ir(H)(X)(NSiN)(coe)] (X = Cl and OTf) complexes,^29^ the bonding situation in the considered [IrH(X)(κ^2^-NSi^tBu2^)(L)] (*X* = Cl and OTf; L = coe, PCy_3_, and PH^t^Bu_2_) species is best described as possessing a dative LP(N) → Ir bond (where LP(N) refers to the nitrogen lone pair) and a covalent (i.e., electron-sharing) Ir–Si bond. Indeed, the NOCV approach confirms the occurrence of three main orbital interactions in these species, namely, the covalent σ–Ir–Si bond (denoted as ρ_1_, Figure 6), the dative bond involving the donation from the lone-pair of the pyridine nitrogen atom to a vacant d atomic orbital of the transition metal (denoted as ρ_2_), and the π-backdonation from a doubly occupied d atomic orbital of the iridium center to a vacant p_π_(Si) atomic orbital of the silicon atom (denoted as ρ_3_, Figure 6). Consistent with the data in Table 4, which gathers the EDA-NOCV results analyzing the interaction between neutral [NSi^tBu2^]^•^ and [Ir(*H*)L(X)]^•^ fragments, the covalent Ir–Si bond (ρ_1_) is almost three times stronger than the dative LP(N) → d(Ir) bond (ρ_2_), while the π-backdonation (ρ_3_) is comparatively much weaker. Although it seems that the Ir–Si bond becomes slightly weaker in the aquo-complexes, in all cases, it is confirmed that the Ir–Si bond is relatively strong regardless of the ligands directly attached to the transition metal center. Nevertheless, it becomes evident that the replacement of the chloride ligand by OTf leads to stronger Ir–Si bonds (compare **1** vs **4** or **1**-H_2_O vs **4-**H_2_O) because of the higher electron-withdrawing character of the triflate ligand, which further polarizes the Ir–Si interaction as confirmed by the enhancement of both the electrostatic (Δ*E*_elstat_) and orbital (Δ*E*_orb_) attractions.

**Table 4:** EDA-NOCV Results (in kcal/mol) and NBO Data for Complexes **1**, **3b**, **4**, and Their Corresponding Water Complexes Computed at the ZORA-BP86-D3/TZ2P//BP86-D3/def2-SVP Level

	**1**	**4**	**3b**	**1**-H_2_O	**4**-H_2_O	**3b**-H_2_O
Δ*E*_int_	–142.7	–149.6	–158.7	–141.8	–151.0	–146.0 (−110.4)^a^
Δ*E*_Pauli_	329.6	351.4	330.0	320.9	324.3	317.2 (−79.4)^a^
Δ*E*_elstat_	–277.9	–284.7	–264.3	–273.8	–279.4	–270.8 (−123.1)^a^
Δ*E*_orb_	–173.2	–190.1	–200.4	–166.4	–171.6	–164.7 (−50.0)^a^
Δ*E*(ρ_1_) (σ–Ir–Si)	–93.0	–103.6	–104.7	–90.3	–90.7	–88.7
Δ*E*(ρ_2_) (LP(N) → d(Ir))	–33.4	–35.8	–33.8	–31.2	–33.9	–29.9
ΔE(ρ_3_) (d(Ir) → π*(Si))	–11.1	–10.7	–13.1	–10.5	–10.6	–12.2
Δ*E*_disp_	–21.2	–26.2	–24.0	–22.4	–24.3	–27.8 (−16.8)^b^
r(Si–Ir)/Å	2.290	2.310	2.303	2.311	2.298 (2.291)	2.307 (2.427)^b^
WBI (Si–Ir)	0.69	0.67	0.72	0.67	0.73	0.75 (0.09)^a^

a Δ*E*_int_ = Δ*E*_elstat_ + Δ*E*_Pauli_ + Δ*E*_orb_ + Δ*E*_disp_ (see computational details).

b Data within parentheses refer to the [Ir]^+^···(OTf)^−^ interaction.

The remarkable strength of the Ir–Si bond in the considered [IrH(X)(κ^2^-NSi^tBu2^)(L)] (X = Cl and OTf; L = coe, PCy_3_, and PH^t^Bu_2_) complexes is reflected in both the markedly short Ir–Si bond (ranging from 2.290 to 2.310 Å, see Table 4) and the high Ir–Si Wiberg bond order (WBI ≈ 0.70). Both values resemble those observed for the related Ir-(*fac*-κ^3^-NSiN) complexes, which also feature strong Ir–Si bonds exhibiting rather short Ir–Si bond lengths (ranging from 2.220 to 2.235 Å).^29^

**Figure 7:**
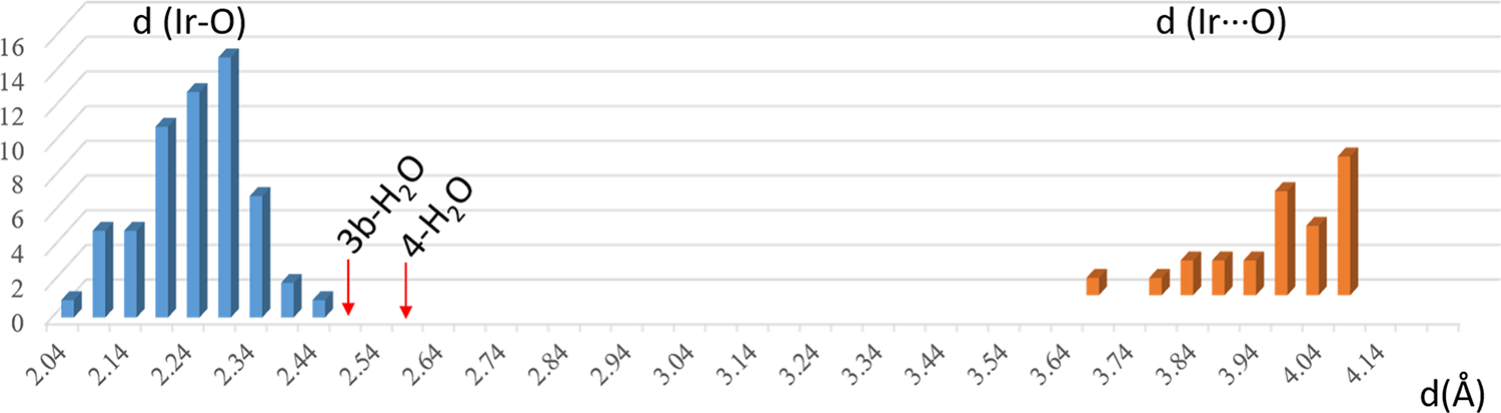
Histogram of the statistical analysis of Ir–O distances in Ir–OTf fragments. Results for searches 1 and 2 are depicted in blue and orange, respectively. The Ir···O distances found in **3b-**H_2_O and **4-**H_2_O complexes are indicated by arrows.

The remarkable long Ir···OTf distance observed in the solid state of the aquo-complexes **3b-**H_2_O and **4-**H_2_O (2.4921(11) and 2.6073(1) Å, respectively) poses the question of whether there is indeed an interaction between the transition metal and the oxygen atom of the triflate ligand or the complexes are better described as ion pairs therefore dominated by electrostatic interactions. To further understand the character of the Ir–OTf bond in **3b-**H_2_O and **4-**H_2_O, two searches were carried out in the Cambridge Structural Database (CSD version 5.43 update Jun 2022),^30^ with structures containing no error and an *R* factor of <0.1. One referred to chemical bonds where the Ir–OTf fragment has been included with the Ir–O bond type defined as “any”. This search led to 41 crystal structures with 60 Ir···O distances in the (2.04–2.402.40) Å range. The other search, considering inter- and intramolecular contacts up to a 4.02 Å value (sum of the van der Waals radii: +0.5 Å), led to 26 crystal structures, with 26 Ir···O distances in the (3.60–4.014.01 ) Å range (Figure 7).

Figure 7 shows that the Ir···O distances values found in **3b-**H_2_O and **4-**H_2_O (2.4921(11) and 2.6073(1) Å) lie between the longest distance reported for an Ir–OTf bond (2.401(12) Å) in ((2,4-dimethylpenta-1,3-dien-5-oyl)-tris(triphenylphosphine)Ir OTf)^31^ and the shortest value found for an interaction (3.606 Å in carbonyl-(pyridine)-bis(tris(biphenyl-4-yl)phosphine)Ir OTf).^32^

Moreover, our gas-phase calculations on **3b**-H_2_O as a representative triflate complex concur quite well with the experiment and nicely reproduce the observed long Ir···OTf distance (2.427 Å). This long bond distance is translated into a WBI of 0.09, which is much lower than that computed for its non-aquo complex counterpart **3b** (0.20, 2.216 Å). Our EDA-NOCV calculations indicate that the [Ir]^+^···(OTf)^−^ interaction is mainly electrostatic as the Δ*E*_elstat_ term contributes ca. 65% to the total attractive interactions (Δ*E*_elstat_ + Δ*E*_orb_ + Δ*E*_disp_) between the [Ir]^+^ and (OTf)^−^ fragments (see Table 4). Despite that, the orbital term (Δ*E*_orb_) is not negligible (contributing ca. 26% to the total bonding) and is mainly dominated by the dative bond involving the donation from the lone pair of the oxygen atom of the triflate to a vacant d atomic orbital of the transition metal. Therefore, it can be concluded that complex **3b**-H_2_O presents an intermediate situation between an ion pair and a standard donor–acceptor complex.

### [IrH(X)(κ^2^-NSi^tBu2^)(L)] (X = Cl and OTf; L = coe, PCy_3_, and PH^t^Bu_2_)-Catalyzed Hydrolysis of HSiMe(OSiMe_3_)_2_

The presence of the triflate ligand is a key factor in the activity of [IrH(X)(κ^2^-NSi^tBu2^)(L)] (X = Cl and OTf; L = coe, PCy_3_, and PH^t^Bu_2_) species as HSiMe(OSiMe_3_)_2_ hydrolysis catalysts. Thus, while complexes **3a**, **3b**, and **4** (0.5 mol %) catalyze the solventless hydrolysis of HSiMe(OSiMe_3_)_2_ at 323 K, under the same reaction conditions, the chloride derivatives **1**, **2a**, and **2b** are not active.

**Figure 8:**
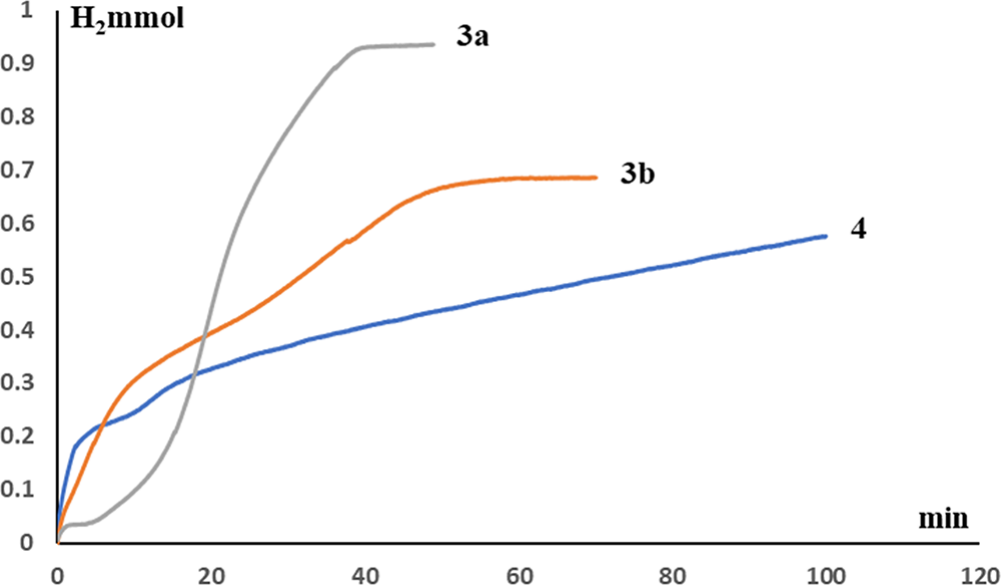
Mol of H_2_*vs* time observed during the **3a**-, **3b**-, and **4**-catalyzed (0.1 mol %) solventless hydrolysis of HSiMe(OSiMe_3_)_2_ at 323 K.

In all the cases, the catalytic reactions were performed in a microreactor and monitored by measuring the hydrogen pressure generated during the hydrolysis processes. The resulting liquid residues were studied by ^1^H and ^29^Si NMR spectroscopy to identify the Si-containing reaction products. These studies confirm that **3a** (TOF_1/2_ = 284 h^–1^) is more active catalyst than **3b** (TOF_1/2_ = 190 h^–1^) and **4** (TOF_1/2_ = 84 h^–1^) for the generation of H_2_ from the hydrolysis of HSiMe(OSiMe_3_)_2_ at 323 K (Figure 8).

**Figure 9:**
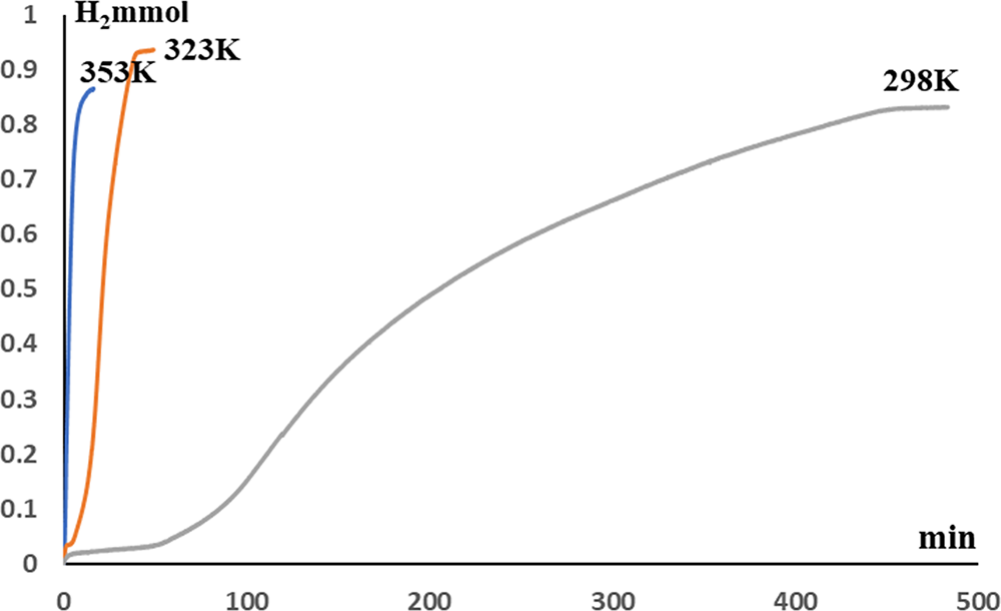
Mol of H_2_*vs* time observed during the **3a**-catalyzed (0.1 mol %) solventless hydrolysis of HSiMe(OSiMe_3_)_2_ at 298, 323, and 353 K.

The activity of the **3a** as a catalyst for the solventless hydrolysis of HSiMe(OSiMe_3_)_2_ depends on the reaction temperature. The best activity has been found at 353 K (TOF_1/2_ = 2000 h^–1^). This value is the highest catalytic activity so far reported for a catalytic solventless hydrolysis of HSiMe(OSiMe_3_)_2_. Moreover, while at 353 K, the catalytic system does not require an activation period; at 323 and 298 K, activation periods of around 7 and 56 min were required, respectively (Figure 9). However, at 353 K, a decrease in selectivity is observed and a mixture of the silanol HOSiMe(OSiMe_3_)_2_ (88 mol %) and the siloxane O{SiMe(OSiMe_3_)_2_}_2_ (12 mol %) was obtained.

The selectivity of the catalytic process clearly depends on the ancillary ligand (coe, PCy_3_, or PH^t^Bu_2_). Thus, the ^1^H and ^29^Si NMR (CD_2_Cl_2_) spectra of the oily residue obtained from the **3a**-catalyzed (0.5 mol %) reaction show the full conversion of the starting hydrosiloxane to give HOSiMe(OSiMe_3_)_2_^9c^ as a major reaction product (>94 mol %) together with O{(SiMe(OSiMe_3_)_2_}_2_ (≈ 4 mol %)^20a^ and traces of other unidentified products (Figure S42). Under the same reaction conditions, but using **3b** (0.5 mol %) instead of **3a** as a catalyst precursor, a lack of selectivity (HOSiMe(OSiMe_3_)_2_ ≈ 84 mol %; O{(SiMe(OSiMe_3_)_2_} ≈ 12 mol %) was observed (Figure S43). Moreover, when the reactions were performed in the presence of catalytic amounts of **4** (0.5 mol %), a complicated mixture of products, consisting of HOSiMe(OSiMe_3_)_2_ (≈ 45 mol %) and O{(SiMe(OSiMe_3_)_2_} (≈ 18 mol %) together with other unidentified species, was obtained (Figure S44). Therefore, the best catalytic performance for the selective solventless hydrolysis of HSiMe(OSiMe_3_)_2_ to HOSiMe(OSiMe_3_)_2_ was obtained using **3a** as a catalytic precursor at 323 K.

^1^H-^29^Si HMBC NMR studies of the oily product obtained from the **3a**-catalyzed hydrolysis of HSiMe(OSiMe_3_)_2_ show that along with the signals due to the reaction product, HOSiMe(OSiMe_3_)_2_, a distinct signal appears corresponding to the correlation of a singlet at δ 1.04 ppm in ^1^H NMR due to the Si-^t^Bu_2_ protons, with a resonance that appears at δ −7.7 ppm in the ^29^Si{^1^H} NMR spectra. This value is upper-field shifted with respect to the value of around δ 43–45 ppm found for the Ir–Si bond in Ir-(κ^2^-NSi^tBu2^) species and δ 12.0 ppm reported for the ligand precursor.^22^ This observation allows us to conclude that catalyst deactivation involving hydrolysis of the Ir–Si bond takes place during the catalytic reaction. Accordingly, when an extra 1.0 mmol of HSiMe(OSiMe_3_)_2_ is added to the reactor at 323 K, a clear decrease in activity is observed. Indeed, it was only possible to achieve the hydrolysis of the 80% of the starting hydrosiloxane before catalyst deactivation (Figure S41).

To explore the scope of the reaction, we have also studied the potential of **3a** as a catalyst for the hydrolysis of HSi(OSiMe_3_)_3_. The results of these studies show that in the presence of catalytic amounts of **3a** (0.5 mol %) at 323 K, the addition of 10 μL of H_2_O to HSi(OSiMe_3_)_3_ did not produce evolution of gas. Heating at 353 K, a very slow generation of H_2_ was observed. These results suggest that the greater steric protection of the Si–H bond in HSi(OSiMe_3_)_3_ hinders its hydrolysis.

## Conclusions

The reaction of the iridium(III) complex [Ir(Cl)(κ^2^-NSi^tBu2^)(coe)] (**1**) with 1 equiv of phosphine quantitatively gives the corresponding species [Ir(Cl)(κ^2^-NSi^tBu2^)(L)] (L = PCy_3_, **2a**; PH^t^Bu_2_, **2b**), which reacts with 1 equiv of silver triflate to afford [Ir(OTf)(κ^2^-NSi^tBu2^)(L)] (L = PCy_3_, **3a** and PH^t^Bu_2_, **3b**). The reversible coordination of water to **4**, **3a**, and **3b** to afford the corresponding adduct [Ir(OTf)(κ^2^-NSi^tBu2^)(L)(H_2_O)] (L = coe, **4**-H_2_O; PCy_3_, **3a**-H_2_O; PH^t^Bu_2_, **3b**-H_2_O) has been demonstrated by means of NMR studies. Moreover, the solid-state structures of complexes **4**-H_2_O and **3b**-H_2_O have been determined by X-ray diffraction studies.

The results of our computational (EDA-NOCV) analysis of the interaction between neutral [NSi^tBu2^]^•^ and [Ir(*H*)L(X)]^•^ fragments in Ir-NSi^tBu2^ species show that the covalent (i.e., electron-sharing) Ir–Si bond is almost three times stronger than the dative LP(N) → d(Ir) bond, while the π-backdonation (ρ_3_) is comparatively much weaker. The coordination of the water molecule produces a slight weakening of the Ir–Si bond. In all the studied cases, it becomes evident that the replacement of the chloride ligand by OTf leads to stronger Ir–Si bonds due to its higher electron-withdrawing character, which further polarizes the Ir–Si bond.

The activity of Ir-(κ^2^-NSi^tBu2^) species as a catalyst for the hydrolysis of HSiMe(OSiMe_3_)_2_ depends on the nature of the ancillary ligands. Thus, while the triflate derivatives are active, the related chloride species show no measurable activity. The best catalytic performance has been obtained when using complexes **3a**, with triflate and PCy_3_ ligands, as catalyst precursors at 323 K, which allows the selective obtention of the corresponding silanol (TOF_1/2_ = 284 h^–1^). NMR studies confirm that the hydrolysis of the Ir–Si bond in **3a** during the process results in the deactivation of the catalyst.

## Experimental Section

### General Information

All manipulations were performed with rigorous exclusion of air at an argon/vacuo manifold using standard Schlenk-tube or glovebox techniques. Solvents were dried by the usual procedures and distilled under argon prior to use. ^1^H, ^13^C{^1^H}, ^31^P{^1^H}, ^29^Si{^1^H}, and ^19^F{^1^H} NMR spectra were recorded on a Bruker ARX or Bruker Avance 300 MHz instrument. Chemical shifts (expressed in parts per million) are referenced to residual solvent peaks (^1^H and ^13^C{^1^H}) and 85% H_3_PO_4_ (^31^P{^1^H}). Coupling constants *J* are given in hertz. Infrared spectra were recorded on a JASCO FT-IR 6600 spectrometer. [IrH(Cl)(κ^2^-NSi^tBu2^)(coe)] (**1**) and [IrH(OTf)(κ^2^-NSi^tBu2^)(coe)] (**4**) were prepared according to published methodologies.^22^

### Preparation of [IrH(cl)(κ^2^-NSi^tBu2^)(PCy_3_)] (**2a**)

Toluene (8 mL) was added to a mixture of [IrH(Cl)(κ^2^-NSi^tBu2^)(coe)] (**1**) (250 mg, 0.424 mmol) and PCy_3_ (118 mg, 0.424 mmol). The resulting solution was stirred overnight at room temperature. The solvent was removed *in vacuo*, and the residue was washed with hexane (2 × 5 mL) and dried *in vacuo*, at r.t. for 1 h, to give yellow powder of **2a**. Yield: 300 mg (93%). Anal. calcd. for C_32_H_58_ClIrNOPSi: C, 50.60; H, 7.70; N, 1.84. Found: C, 50.91; H, 7.84; N, 2.00. ^1^H NMR (300 MHz, 298 K, C_6_D_6_): δ 8.96 (dd, ^3^*J_H–H_* = 6.2 Hz, ^4^*J_H–P_* = 2.7 Hz 1H, py-*H^6^*), 6.36 (s, 1H, py-*H^3^*), 6.07 (d, ^3^*J_H–H_* = 6.2 Hz, 1H, py-*H^5^*), 2.51 (m, 3H, CH-PCy_3_), 2.29 (m, 3H, CH_2_-PCy_3_), 2.13 (s, 3H, CH_2_-PCy_3_), 1.78 (m, 6H, CH_2_-PCy_3_), 1.75 (m, 3H, CH_2_-PCy_3_), 1.66 (m, 3H, CH_2_-PCy_3_), 1.56 (s, 3H, CH_3_-Py), 1.41 (s, 9 H, CH_3_-^t^Bu), 1.36 (s, 9H, CH_3_-^t^Bu), 1.29 (m, 6H, CH_2_-PCy_3_), 1.21 (m, 3H, CH_2_-PCy_3_), −22.39 (Ir–H, d, ^2^*J_P_*–_*H*_ = 16.2 Hz). ^13^C{^1^H} APT plus HSQC ^1^H–^13^C (75 MHz, 298 K, C_6_D_6_): δ 166.4 (s, ipso-py-*C^2^*), 151.5 (s, ipso-py-*C^4^*), 147.6 (s, py-*C^6^*), 117.5 (s, py-*C^5^*), 110.4 (s, py-*C^3^*), 37.1 (d, ^1^*J_P_*–_*C*_ = 30 Hz, 3C, CH-PCy_3_), 32.2 (s, 6C, CH_3_-^t^Bu), 32.1 (s, 3C, CH_2_-PCy_3_), 29.9 (s, 3C, CH_2_-PCy_3_), 29.8 (s, 6C, CH_3_-^t^Bu), 27.8 (d, ^2^*J_P–C_* = 15.0 Hz, 3C, CH_2_-PCy_3_), 27.7 (d, ^2^*J_P_*–_*C*_ = 15.7 Hz, 3C, CH_2_-PCy_3_), 26.8 (d, 3C, *J_P_*–_*C*_ = 1.6 Hz CH_2_-PCy_3_), 25.9 (s, 1C, C-^t^Bu), 25.8 (s, 1C, C-^t^Bu), 20.6 (s, *C*H_3_-py). ^31^P{^1^H} NMR (121 MHz, 298 K, C_6_D_6_): δ 11.8 (s). ^29^Si{^1^H} NMR (60 MHz, 298 K, C_6_D_6_) plus HMBC ^1^H–^29^Si: δ 44.1(s). High-resolution mass spectrometry (ESI^+^): calcd. *m*/*z* = 759.3343; found *m*/*z* = 724.3474 (M^+^-Cl). IR: 2244 cm^–1^ (Ir–H).

### Preparation of [IrH(cl)(κ^2^-NSi^tBu2^)(PH^t^Bu_2_)] (**2b**)

A toluene solution (3.0 mL) of PH^t^Bu_2_ (86.3 mg, 0.594 mmol) was added to a toluene (8 mL) solution of [IrH(Cl)(κ^2^-NSi^tBu2^)(coe)] (**1**) (350 mg, 0.594 mmol). The resulting solution was stirred overnight at room temperature. The solvent was removed *in vacuo*, and the residue was washed with hexane (2 × 5 mL) and dried *in vacuo*, at r.t. for 1 h, to give yellow powder of **2b**. Yield: 287 mg (77%). Anal. calcd. for C_22_H_44_ClIrNOPSi: C, 42.26; H, 7.09; N, 2.24. Found: C, 42.10; H, 6.76; N, 2.18. ^1^H NMR (300 MHz, 298 K, C_6_D_6_): δ 9.12 (dd, ^3^*J_H–H_* = 6.2 Hz, ^4^*J_H–P_* = 2.7 Hz 1H, py-*H*^6^), 6.37 (s, 1H, py-*H*^3^), 6.07 (psd, ^3^*J_H–H_* = 6.2 Hz, 1H, py-*H^5^*), 4.98 (dd, ^1^*J_P-H_* = 346 Hz, ^3^*J_H–H_* = 2.9 Hz, 1H, P-H), 1.55 (d, ^4^*J_H–P_* = 14.3 Hz, 1H 9 H, P-^t^Bu), 1.54 (s, 3H, Me-py), 1.38 (d, 9H, ^4^*J_H–P_* = 13.4 Hz P-^t^Bu), 1.32 (s, 18H, CH_3_-^t^Bu), 1.31 (s, 18H, CH_3_-^t^Bu), −23.82 (Ir–H; dd, ^2^*J_P–H_* = 16.1 Hz, ^4^*J_H–H_* = 2.9 Hz, 1H). ^13^C{^1^H} APT plus HSQC ^1^H–^13^C (75 MHz, 298 K, C_6_D_6_): δ 167.0 (ipso, py -*C*^2^), 152.0 (ipso, py-*C*^4^), 147.1 (s, py-C^6^), 117.6 (d, *J_P–C_* = 2.5 Hz, py-*C*^5^), 110.8 (s, *J_P–C_* = 1.7 Hz, py-*C*^3^), 35.3 (d, 1C, ^1^*J_P–C_* = 30.3 Hz P-^t^Bu), 33.2 (d, 1C, ^1^*J_P–C_* = 25.5 Hz P-^t^Bu), 32.7 (d, 3C, ^2^*J_P_*–_*C*_ = 3.2 Hz P-^t^Bu), 32.4 (d, 3C, ^2^*J_P–C_* = 3.2 Hz P-^t^Bu), 31.1 (s, 3C, CH_3_-^t^Bu), 29.7 (s, 3C, CH_3_-^t^Bu), 20.6 (s, *C*H_3_-py). ^31^P{^1^H} NMR (121 MHz, 298 K, C_6_D_6_): δ 26.9 (s).^29^Si{^1^H} NMR (60 MHz, 298 K, C_6_D_6_) plus HMBC ^1^H–^29^Si: δ 43.3(s). High-resolution mass spectrometry (ESI^+^): calcd. *m*/*z* = 625.2247; found *m*/*z* = 590.2519 (M^+^-Cl). IR: 2266 cm^–1^ (Ir–H).

### Preparation of [IrH(OTF)(κ^2^-NSi^tBu2^)(PCy_3_)] (**3a**)

Toluene (8 mL) was added to a mixture of complex **2a** (269 mg, 0.354 mmol) and silver triflate (90.9 mg, 0.354 mmol). The resulting suspension was stirred overnight at room temperature. After which, a yellow solution was filtered through celite, and the solvent was removed *in vacuo*. The resulting residue was washed with pentane (2 × 5 mL) and dried *in vacuo*, at r.t. for 1 h, to give off-white powder of **3a**. Yield: 253.5 mg (82%). ^1^H NMR plus HSQC ^1^H–^13^C (300 MHz, 298 K, C_6_D_6_): δ 8.71 (dd, ^3^*J_H–H_* = 6.2 Hz, ^4^*J_H–P_* = 2.9 Hz 1H, py-*H*^6^), 6.32 (s, 1H, py-*H^3^*), 6.16 (d, ^3^*J_H–H_* = 6.2 Hz, 1H, py-*H*^5^), 2.40 (m, 3H, CH-PCy_3_), 2.17 (m, 3H, CH_2_-PCy_3_), 2.06 (s, 3H, CH_2_-PCy_3_), 1.74 (m, 9H, CH_2_-PCy_3_), 1.65 (m, 3H, CH_2_-PCy_3_), 1.64 (m, 3H, CH_2_-PCy_3_), 1.62 (m, 3H, CH_2_-PCy_3_), 1.51 (s, 3H, CH_3_-Py), 1.49 (m, 3H, CH_2_-PCy_3_), 1.36 (s, 9H, CH_3_-^t^Bu), 1.21 (s, 9H, CH_3_-^t^Bu), 1.20 (m, 3H, CH_2_-PCy_3_), −29.21 (Ir–H; d, ^2^*J_P–H_* = 17.1 Hz). ^13^C{^1^H} APT plus HSQC ^1^H–^13^C (75 MHz, 298 K, C_6_D_6_): δ 165.4 (s, ipso-py-*C*^2^), 153.2 (s, ipso-py-*C*^4^), 147.8 (s, py-*C*^6^), 118.4 (s, py-*C*^5^), 111.1 (s, py-*C^3^*), 36.2 (d, ^1^*J_P–C_* = 27.4 Hz, 3C, CH-PCy_3_), 31.4 (s, 3C, CH_2_-PCy_3_), 31.2 (s, 3C, CH_3_-^t^Bu), 29.6 (s, 3C, CH_2_-PCy_3_), 29.4 (s, 3C, CH_3_-^t^Bu), 27.7 (d, ^2^*J_P–C_* = 10.9 Hz 3C, CH_2_-PCy_3_), 27.5 (d, *J_P–C_* = 11.1 Hz, 3C, CH_2_-PCy_3_), 26.6 (d, 3C, *J_P–C_* = 1.7 Hz CH_2_-PCy_3_), 25.9 (s, 1C, C-^t^Bu), 25.3 (s, 1C, C-^t^Bu), 20.7 (s, *C*H_3_-py). ^31^P{^1^H} NMR (121 MHz, 298 K, C_6_D_6_): δ 10.3 (s). ^29^Si{^1^H} NMR (60 MHz, 298 K, C_6_D_6_) plus HMBC ^1^H–^29^Si: δ 44.5 (s). ^19^F{^1^H} NMR (282 MHz, 298 K, C_6_D_6_): δ −77.79 (s, OTf). High-resolution mass spectrometry (ESI^+^): calcd. *m*/*z* = 873.3174; found *m*/*z* = 724.3515 (M^+^-OTf). IR: 2309 cm^–1^ (Ir–H).

### Preparation of [IrH(OTf)(κ^2^-NSi^tBu2^)(PH^t^Bu_2_)] (**3b**)

Toluene (8 mL) was added to a mixture of complex **2b** (228 mg, 0.365 mmol) and silver triflate (93.7 mg, 0.365 mmol). The resulting suspension was stirred overnight at room temperature. A yellow solution was filtered through celite, and the solvent was removed *in vacuo*. The residue was washed with pentane (2 × 5 mL). and dried *in vacuo*, at r.t. for 1 h, to give off-white powder of **3b**. Yield: 212 mg (79%). ^1^H NMR (300 MHz, 298 K, C_6_D_6_): δ 8.70 (dd, ^3^*J_H–H_* = 6.0 Hz, ^4^*J_H–P_* = 2.9 Hz 1H, py-*H*^6^), 6.29 (s, 1H, py-*H*^3^), 6.18 (d, ^3^*J_H–H_* = 6.0 Hz, 1H, py-*H*^5^), 4.85 (dd, ^1^*J_P–H_* = 355 Hz, ^3^*J_H–H_* = 2.4 Hz, 1H, P–H), 1.45 (d, 9H^4^*J_H–P_* = 17.1 Hz P-^t^Bu), 1.38 (d, 9H, ^4^*J_H-P_* = 17.1 Hz P-^t^Bu), 1.31 (s, 3H, Me-py), 1.29 (s, 9H, P-^t^Bu), 1.14 (s, 9H, CH_3_-^t^Bu), −29.31 (Ir–H; dd, ^1^*J_P–H_* = 17.1 Hz, ^3^*J_H–H_* = 2.4 Hz). ^13^C{^1^H} APT plus HSQC ^1^H–^13^C (75 MHz, 298 K, C_6_D_6_): δ 165.4 (ipso, py -*C*^2^), 153.6 (ipso, py-*C*^4^), 147.4 (s, py-C^*6*^), 118.5 (d, J_*P–C*_ = 3.0 Hz, py-*C*^5^), 111.2 (d, J_*P–C*_ = 1.9 Hz, py-*C^3^*), 36.4 (d, 1C, ^1^*J_P–C_* = 30.2 Hz P-^t^Bu), 36.3 (d, 1C, ^1^*J_P–C_* = 30.2 Hz P-^t^Bu), 32.3 (d, 3C, ^2^*J_P–C_* = 3.4 Hz, *C*H_3_-P-^t^Bu), 32.0 (d, 1C, ^1^*J_P–C_* = 24.2 Hz, *C*-P-^t^Bu), 31.3 (d, 3C, ^2^*J_P–C_* = 4.1 Hz, *C*H_3_-P-^t^Bu), 30.8 (s, 3C, CH_3_-^t^Bu), 29.2 (s, 3C, CH_3_-^t^Bu), 25.9 (d, J_*P–C*_ = 2.1 Hz, 1C, C-^t^Bu), 25.3 (s, 1C, C-^t^Bu), 20.7 (s, *C*H_3_-py). ^31^P{^1^H} NMR (121 MHz, 298 K, C_6_D_6_): δ 27.9 (s). ^29^Si{^1^H} NMR (60 MHz, 298 K, C_6_D_6_) plus HMBC ^1^H–^29^Si: δ 45.4 (s). ^19^F{^1^H} NMR (282 MHz, 298 K, C_6_D_6_): δ −77.5 (s, OTf). High-resolution mass spectrometry (ESI^+^): calcd. *m*/*z* = 739.2079; found *m*/*z* = 590.2540 (M^+^-OTf). IR: 2338 cm^–1^ (Ir–H).

### Reactions of HSiMe(OSiMe_3_)_2_ with Water Catalyzed by [IrH(X)(κ^2^-NSi^tBu2^)(L)] Species (X = Cl and OTf; L = coe, PCy_3_, and PH^t^Bu_2_)

The reactions were carried out in a Man on the Moon X102 kit microreactor^33^ with a total volume of 14.2 mL at 298, 323, and 353 K. In a typical procedure, 1.0 mmol of siloxane (HSiMe(OSiMe_3_)_2_, 272 μL) was added to the catalyst **3a**, **3b**, and **4** (0.005 mmol, 4.36 mg; 3.7 mg; 3.5 mg) under argon. The reactor was placed into an isothermal bath at the corresponding temperature. Once the pressure was stabilized, water (50 μL) was added with a syringe. Hydrogen evolution was measured until the inner pressure in the microreactor remained constant. The difference in pressure was used to calculate the amount of H_2_ produced during the reaction using the ideal gas law, *P*·*V* = *n*·*R*·*T*. Once the reaction finished, the product was analyzed by ^1^H and ^29^Si{^1^H} NMR spectroscopy.

### Selected Data for HOSiMe(OSiMe_3_)_2_

^29^Si{^1^H} plus ^1^H-^29^Si HMQC NMR (79.5 MHz, CD_2_Cl_2_, 298 K): δ 8.6 (s, (Me_3_*Si*O)_2_MeSi), −54.6 (s, (Me_3_SiO)_2_Me*Si*). ^13^C{^1^H}-APT NMR (75.5 MHz, CD_2_Cl_2_, 298 K): δ 1.9 (s, (*Me*_3_SiO)_2_MeSi), −2.6 (s, (Me_3_SiO)_2_*Me*Si).

### Selected Data for {O(SiMe(OSiMe_3_)_2_}_2_

^29^Si{^1^H} NMR plus ^29^Si–^1^H HMBC (60 MHz, C_6_D_6_, 298 K): 7.5 (s, (Me_3_*Si*O)_2_MeSi), −65.5 (s, (Me_3_SiO)_2_Me*Si*).

### Single-Crystal Structure Determination

X-ray diffraction data were collected on an APEX DUO (compound **2b** and **4-H_2_O**) and D8 VENTURE (compounds **2a**, **3a**, and **3b-H_2_O**) Bruker diffractometers, using graphite-monochromated Mo Kα radiation (λ = 0.71073 Å). Single crystals were mounted on a fiber or a MiTeGen support coated with protecting perfluoropolyether oil and cooled to 100(2) K with open-flow nitrogen gas. Data were collected using ω scans (and φ scans in compounds **2a**, **3a**, and **3b-H_2_O** data collection) with narrow oscillation frame strategies. Diffracted intensities were integrated and corrected of absorption effects by using multiscan method using SAINT^34^ and SADABS^35^ programs included in APEX4 packages. Structures were solved by direct methods with SHELXS^36^ and refined by full-matrix least squares on *F*^2^ with the SHELXL program^37^ included in the Wingx program system.^38^

Hydrogen atoms have been observed in Fourier difference maps. Most of them have been included in the models in calculated positions and refined with a riding model. Several strategies, adapted to data and structural model, have been applied to locate and refine hydride ligands. It has been included in the model in observed position and freely refined for compounds **2a**, **3b**-H_2_O, and refined with a restrain in the Ir–H bond length in **4**-H_2_O structure refinement. For compounds **2b** and **3a**, the position of the hydride has been located with potential energy minimization with HYDEX program.^39^ The hydride position has been fixed (**2b**) or refined with a restraint in the Ir–H bond (**3a**).

#### Structural Data for **2a**

C_32_H_58_ClIrNOPSi·C_6_H_14_; *M_r_* = 845.67; yellow prism, 0.140 × 0.140 × 0.240 mm^3^; triclinic *P*1̅; *a* = 9.7011(4) Å, *b* = 12.2283(6) Å, *c* = 17.5724(9) Å, α = 74.379(2)°, β = 89.318(2)°, γ = 83.510(2)°; *V* = 1994.34(16) Å^3^, *Z* = 2, *D*_c_ = 1.408 g/cm^3^; μ = 3.513 cm^–1^; min. and max. absorption correction factors: 0.6437 and 0.7457; 2θ_max_ = 56.65°; 96,004 reflections measured, 9595 unique; *R*_int_ = 0.0270; number of data/restraint/parameters: 9595/0/410; *R*_1_ = 0.0136 [9525 reflections, *I* > 2σ(*I*)], w*R*(*F^2^*) = 0.0331 (all data); largest difference peak: 0.500 e·Å^–3^.

#### Structural Data for **2b**

C_22_H_44_ClIrNOPSi; *M_r_* = 625.29; yellow prism, 0.200 × 0.240 × 0.400 mm^3^; monoclinic P2_1_/*n*; *a* = 11.5133(11) Å, *b* = 15.0495(15) Å, *c* = 16.1930(16) Å, β = 108.2540(10)°; *V* = 2664.6(5) Å^3^, *Z* = 4, *D*_c_ = 1.559 g/cm^3^; μ = 5.229 cm^–1^; min. and max. absorption correction factors: 0.2152 and 0.3669; 2θ_max_ = 60.44°; 64,539 reflections measured, 7655 unique; *R*_int_ = 0.0211; number of data/restraint/parameters: 7655/0/281; *R*_1_ = 0.0130 [7333 reflections, *I* > 2σ(*I*)], w*R*(*F^2^*) = 0.0327 (all data); largest difference peak: 1.172 e·Å^–3^.

#### Structural Data for **3a**

C_33_H_58_F_3_IrNO_4_PSSi; *M_r_* = 873.12; yellow prism, 0.030 × 0.050 × 0.070 mm^3^; monoclinic C2/*c*; *a* = 19.5534(6) Å, *b* = 15.2963(4) Å, *c* = 26.1826(6) Å, β = 110.1530(10)°; *V* = 7351.6(3) Å^3^, *Z* = 8, *D*_c_ = 1.578 g/cm^3^; μ = 3.816 cm^–1^; min. and max. absorption correction factors: 0.6692 and 0.7461; 2θ_max_ = 61.046°; 115,743 reflections measured, 11,152 unique; *R*_int_ = 0.0444; number of data/restraint/parameters: 11,152/1/417; *R*_1_ = 0.0202 [9828 reflections, *I* > 2σ(*I*)], w*R*(*F^2^*) = 0.0455 (all data); largest difference peak: 1.105 e·Å^–3^.

#### Structural Data for **3b-H_2_O**

C_22_H_46_IrNO_2_PSSi·CF_3_O_3_S; *M_r_* = 756.93; yellow prism, 0.180 × 0.200 × 0.240 mm^3^; triclinic P1̅ *a* = 11.0134(8) Å, *b* = 11.4780(9) Å, *c* = 12.8243(9) Å, α = 88.882(2)°, β = 68.872(2)°, γ = 80.166(3)°; *V* = 1488.39(19) Å^3^, *Z* = 2, *D*_c_ = 1.689 g/cm^3^; μ = 4.701 cm^–1^; min. and max. absorption correction factors: 0.5459 and 0.7457; 2θ_max_ = 56.66°; 72,671 reflections measured, 7370 unique; *R*_int_ = 0.0274; number of data/restraint/parameters: 7370/0/351; *R*_1_ = 0.0141 [7296 reflections, *I* > 2σ(*I*)], w*R*(*F^2^*) = 0.0339 (all data); largest difference peak: 0.660 e·Å^–3^.

#### Structural Data for **4-H_2_O**

C_22_H_41_IrNO_2_Si·CF_3_O_3_S; *M_r_* = 720.92; white needle, 0.050 × 0.090 × 0.320 mm^3^; monoclinic P2_1_/*na* = 7.6656(4) Å, *b* = 30.0971(14) Å, *c* = 12.3863(6) Å, β = 95.0090(10)°; *V* = 2846.8(2) Å^3^, *Z* = 4, *D*_c_ = 1.682 g/cm^3^; μ = 4.857 cm^–1^; min. and max. absorption correction factors: 0.3924 and 0.5870; 2θ_max_ = 56.49°; 30,387 reflections measured, 7044 unique; *R*_int_ = 0.0258; number of data/restraint/parameters 7044/2/341; *R*_1_ = 0.0177 [6405 reflections, *I* > 2σ(*I*)], w*R*(*F*^2^) = 0.0376 (all data); largest difference peak: 1.021 e·Å^–3^.

CCDC-2177342-2177346 contains the supplementary crystallographic data for this paper. These data can be obtained free of charge from the Cambridge Crystallographic Data Centre via www.ccdc.cam.ac.uk/data_request/cif.

### Computational Details

Geometry optimizations of the complexes were performed without symmetry constraints using the Gaussian09^40^ energies and gradients at the BP86^42^/def2-SVP^43^ level of theory using the D3 dispersion correction suggested by Grimme et al.^44^ Vibrational analysis was performed to ensure that the optimized geometry corresponds to an energy minimum. NBO calculations were carried out using the NBO6.0 program^45^ at the BP86-D3/def2-SVP level.

The interaction Δ*E*_int_ between the selected fragments is analyzed with the help of the EDA method.^46^ Within this approach, ΔE_int_ can be decomposed into the following physically meaningful terms:
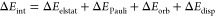


The term Δ*E*_elstat_ corresponds to the classical electrostatic interaction between the unperturbed charge distributions of the deformed reactants and is usually attractive. The Pauli repulsion Δ*E*_Pauli_ comprises the destabilizing interactions between occupied orbitals and is responsible for any steric repulsion. The orbital interaction Δ*E*_orb_ accounts for electron-pair bonding, charge transfer (interaction between occupied orbitals on one moiety with unoccupied orbitals on the other, including HOMO–LUMO interactions), and polarization (empty-occupied orbital mixing on one fragment due to the presence of another fragment). Finally, the Δ*E*_disp_ term takes into account the interactions, which are due to dispersion forces. Moreover, the NOCV^47^ extension of the EDA method has been also used to further partition the Δ*E*_orb_ term. The EDA-NOCV approach provides pairwise energy contributions for each pair of interacting orbitals to the total bond energy.

The program package AMS 2020.101^48^ was used for the EDA-NOCV calculations at the same BP86-D3 level, in conjunction with a triple-ζ-quality basis set using uncontracted Slater-type orbitals (STOs) augmented by two sets of polarization functions with a frozen-core approximation for the core electrons.^49^ Auxiliary sets of s, p, d, f, and g STOs were used to fit the molecular densities and to represent the Coulomb and exchange potentials accurately in each SCF cycle.^50^ Scalar relativistic effects were incorporated by applying the zeroth-order regular approximation (ZORA).^51^ This level of theory is denoted ZORA-BP86-D3/TZ2P//BP86-D3/def2-SVP.

## Supporting Information Available

Additional experimental details, NMR data, and methods and Cartesian coordinates of computed structures (PDF)
