# Supplementary material for: Synthesis and Characterization of Ir-(κ2-NSi) Species Active toward the Solventless Hydrolysis of HSiMe(OSiMe3)2
Source: Inorg Chem. 2022 Oct 4;61(41):16282–94. doi: 10.1021/acs.inorgchem.2c01973 (PMC10468103; doi:10.1021/acs.inorgchem.2c01973)
Supplement: Supplementary file 1 — ic2c01973_si_001.pdf [file ic2c01973_si_001.pdf]

Supporting Information

Synthesis and Characterization of Ir-(κ^2-NSi) Species Active Towards the Solventless Hydrolysis of $HSiMe(OSiMe_3)_2$

Alejandra Gómez-España,[a] Pilar García-Orduña,[a] Jefferson Guzmán,[a] Israel Fernández[b] and Francisco J. Fernández-Alvarez*[a]*

[a]Departamento de Química Inorgánica-Instituto de Síntesis Química y Catálisis Homogénea (ISQCH), Universidad de Zaragoza–CSIC, Facultad de Ciencias, 50009 Zaragoza, Spain, e-mail, F. J. Fernández-Alvarez: paco@unizar.es

[b]Departamento de Química Orgánica I and Centro de Innovación en Química Avanzada, Facultad de Ciencias Químicas, Universidad Complutense de Madrid, 28040-Madrid, Spain, e-mail: israel@quim.ucm.es

1. Spectra

1. 1. Selected spectra of complexes 2a, 2b, 3a y 3b

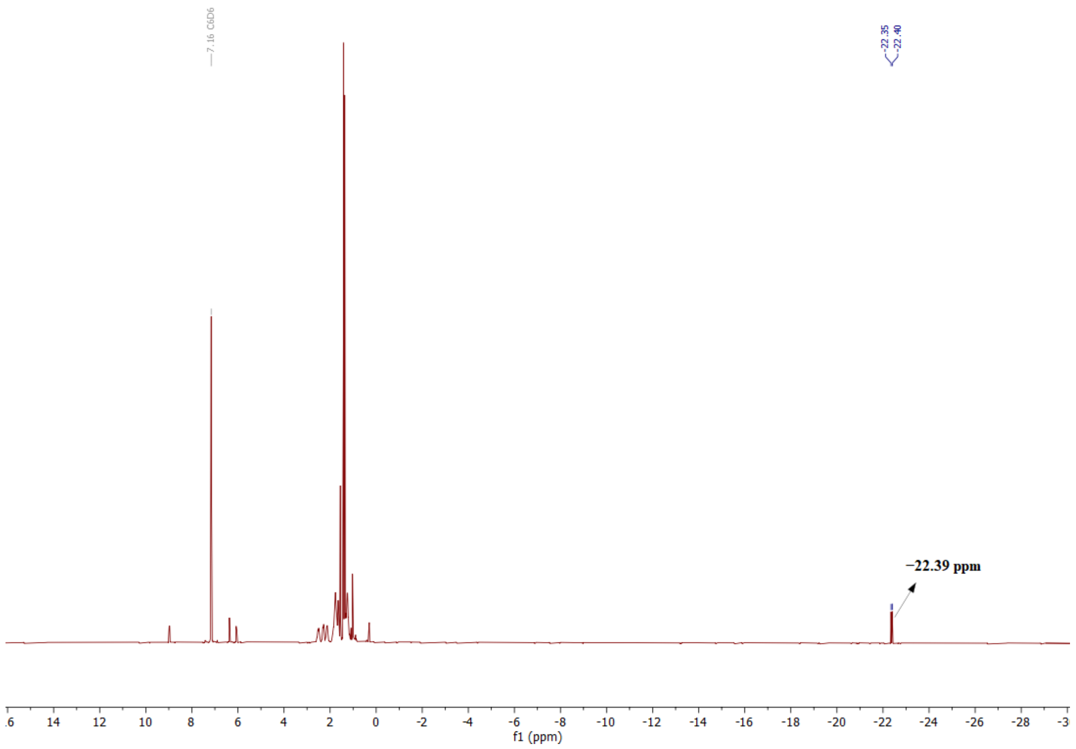

Figure S1. ^{1}H NMR spectrum of **2a** in C_6D_6 (300 MHz, 298K).

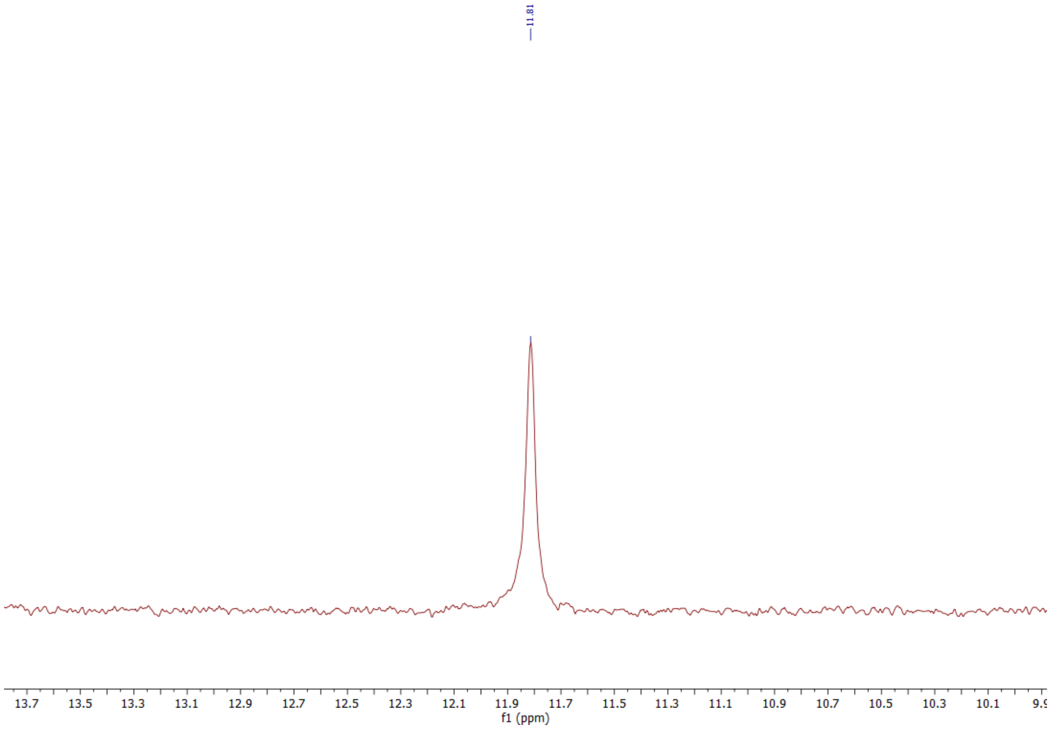

Figure S2. ^{31}P{1H} NMR spectrum of **2a** in C_6D_6 (121 MHz, 298K).

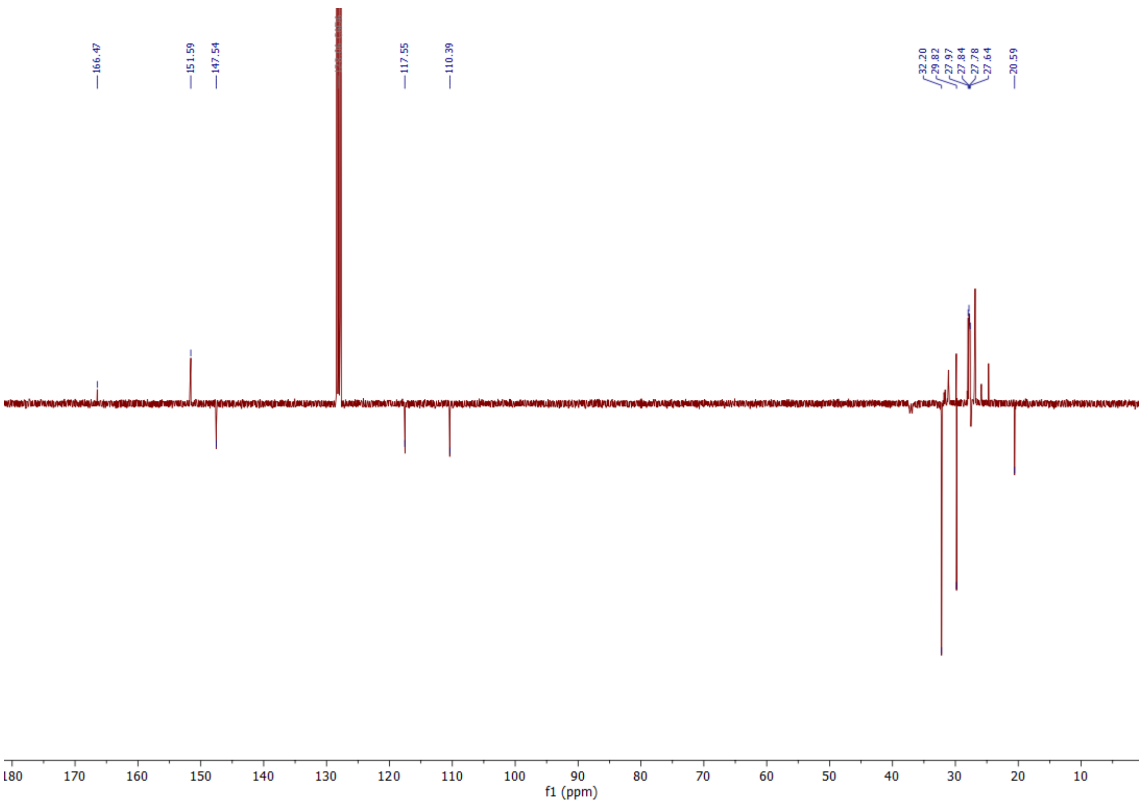

Figure S3. ^{13}C APT NMR spectrum of **2a** in C_6D_6 (75 MHz, 298K).

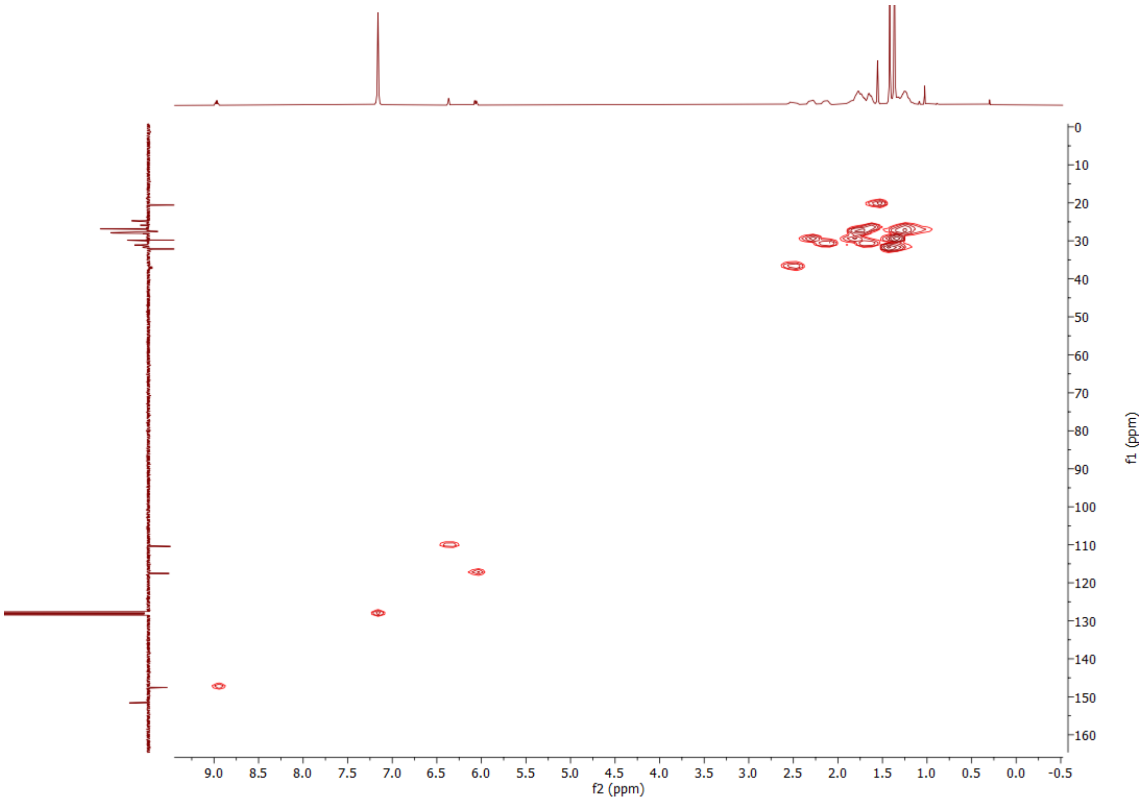

Figure S4. ^{1}H–^{13}C HSQC NMR spectrum of **2a** in C_6D_6 (298K).

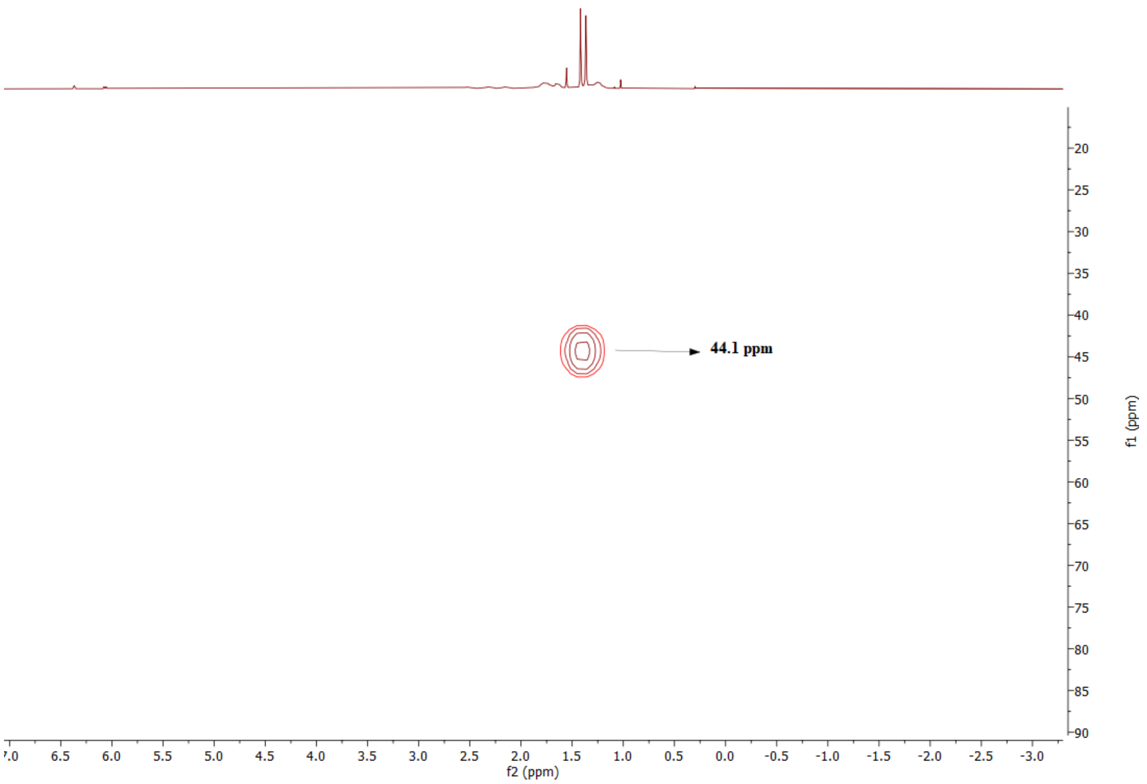

Figure S5. ^{1}H–^{29}Si HMBC spectrum of **2a** in C_6D_6 (60 MHz, 298K).

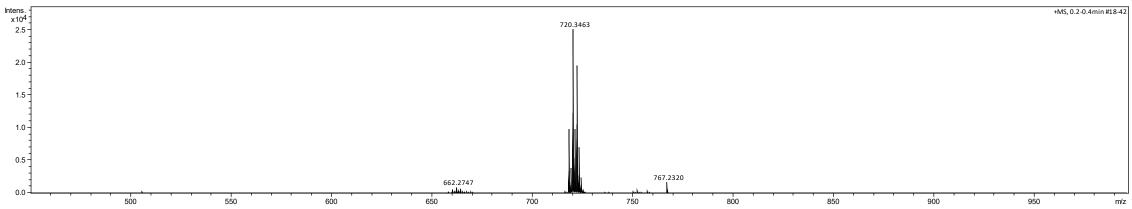

Figure S6. HR-MS of complex **2a.**

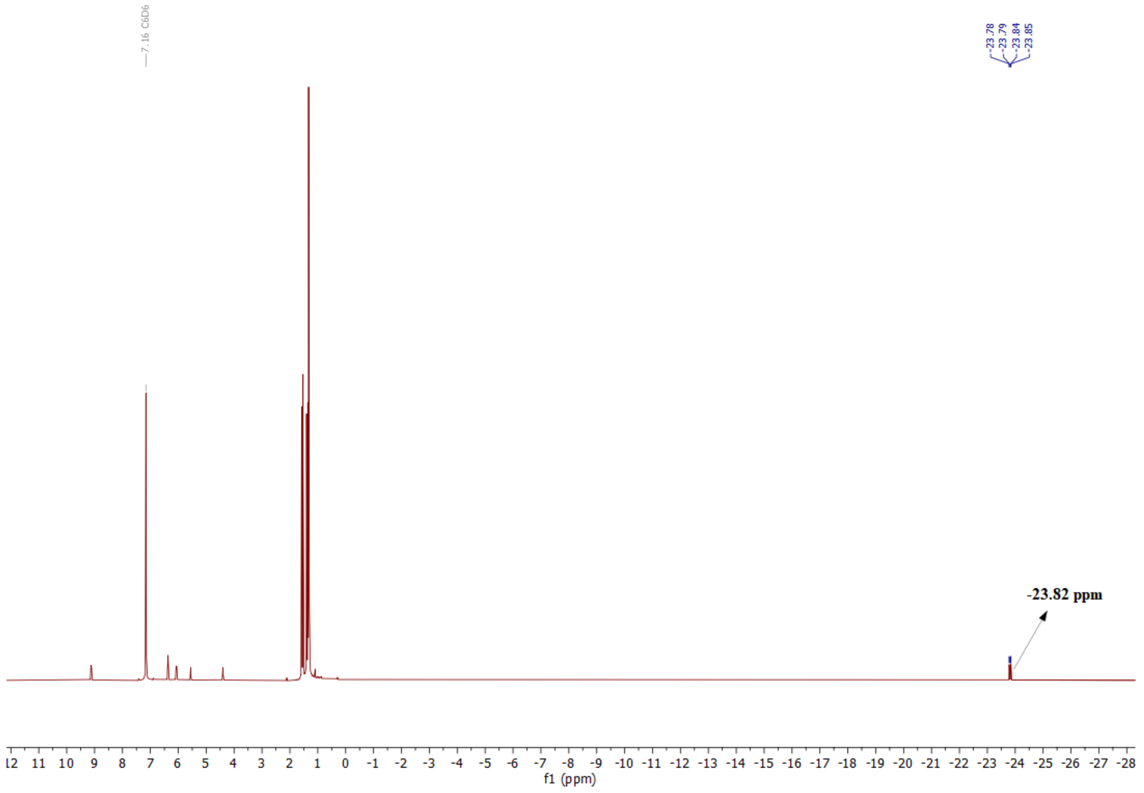

Figure S7. ^{1}H NMR spectrum of **2b** in C_6D_6 (300 MHz, 298K).

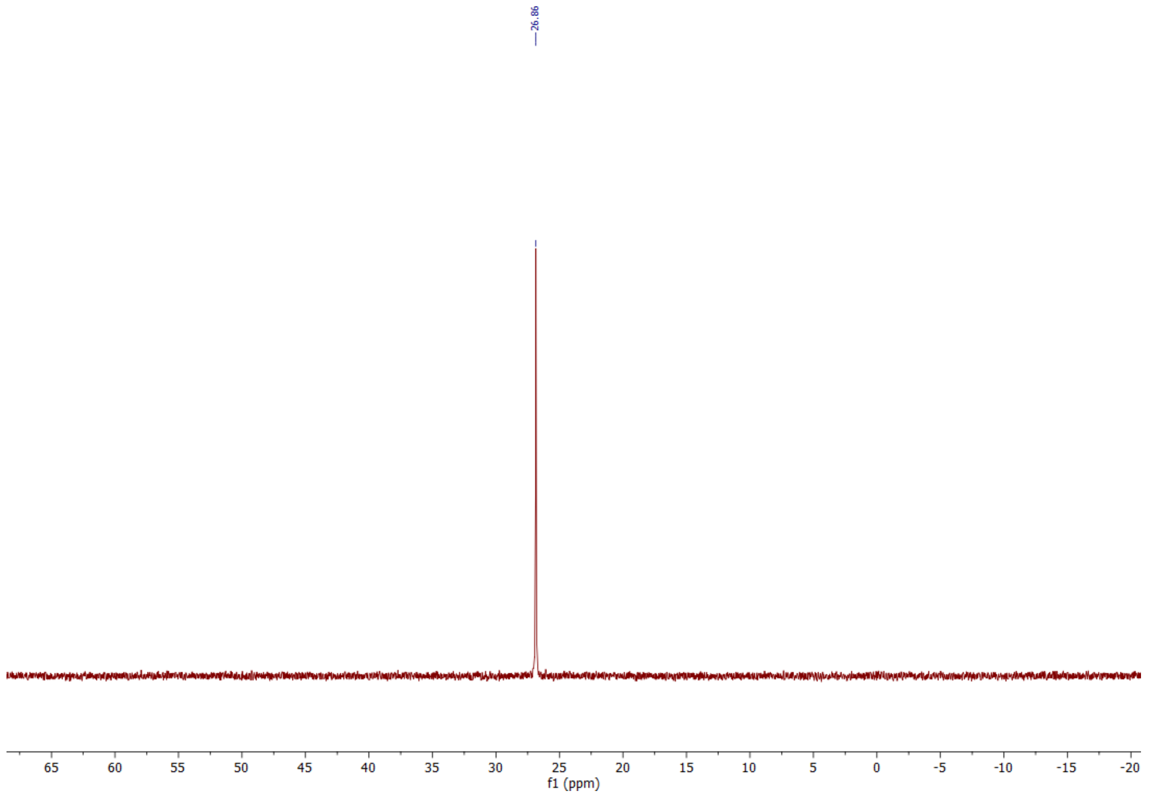

Figure S8. ^{31}P{1H} NMR spectrum of **2b** in C_6D_6 (121 MHz, 298K).

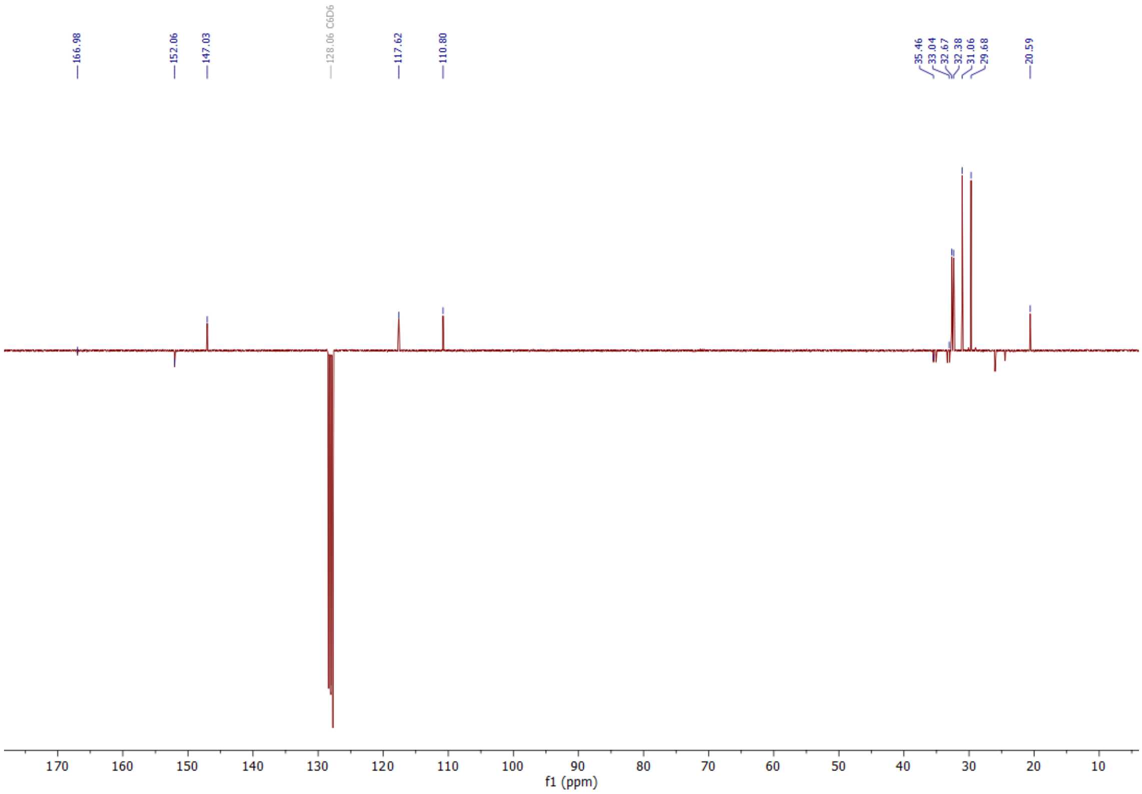

Figure S9. ^{13}C APT NMR spectrum of **2b** in C_6D_6 (75 MHz, 298K).

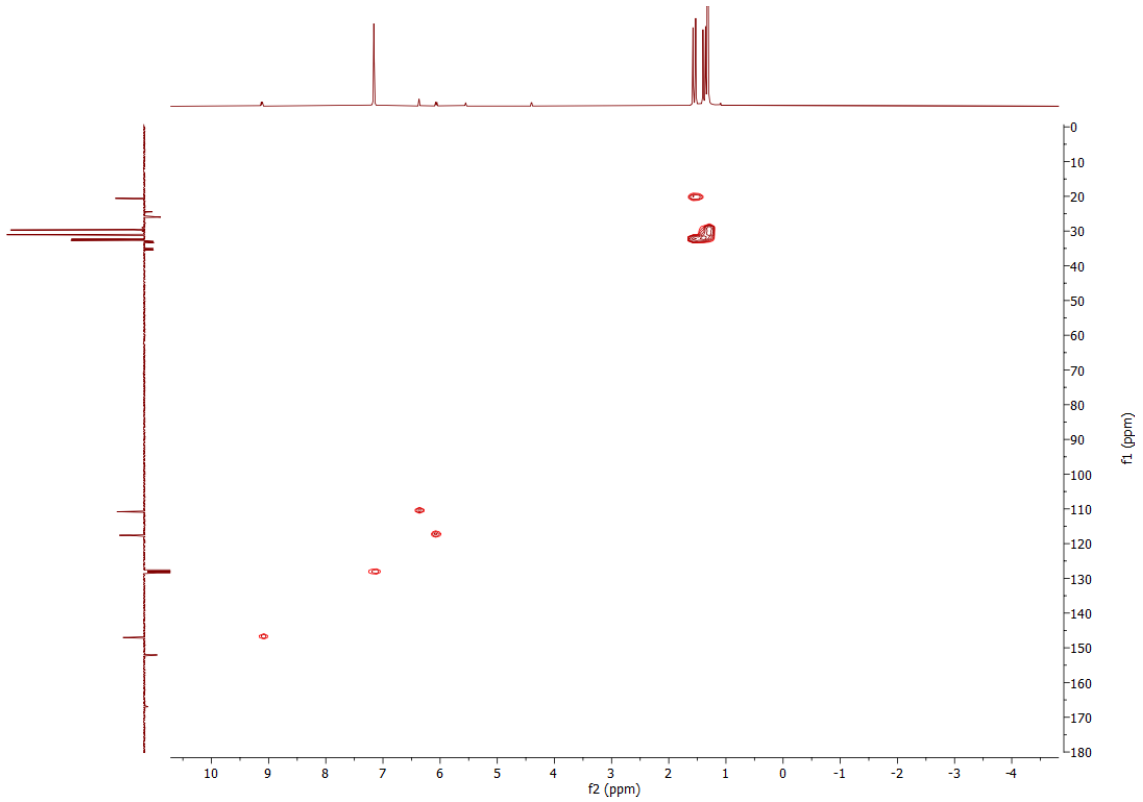

Figure S10. ^{1}H–^{13}C HSQC NMR spectrum of **2b** in C_6D_6 (298K).

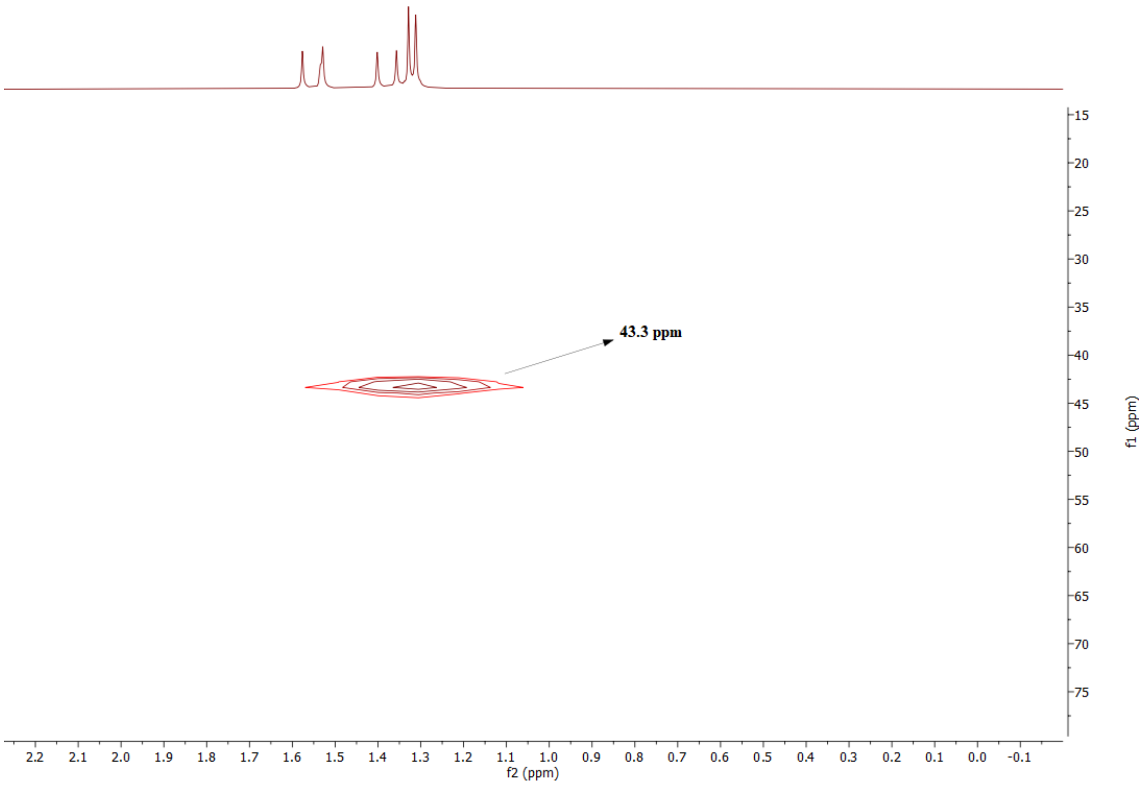

Figure S11. ^{1}H–^{29}Si HMBC spectrum of **2b** in C_6D_6 (60 MHz, 298K).

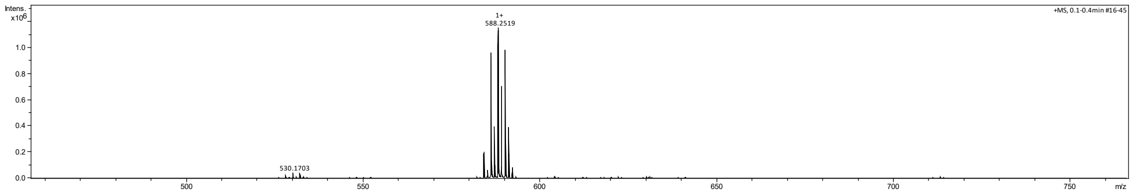

Figure S12. HR-MS of complex **2b.**

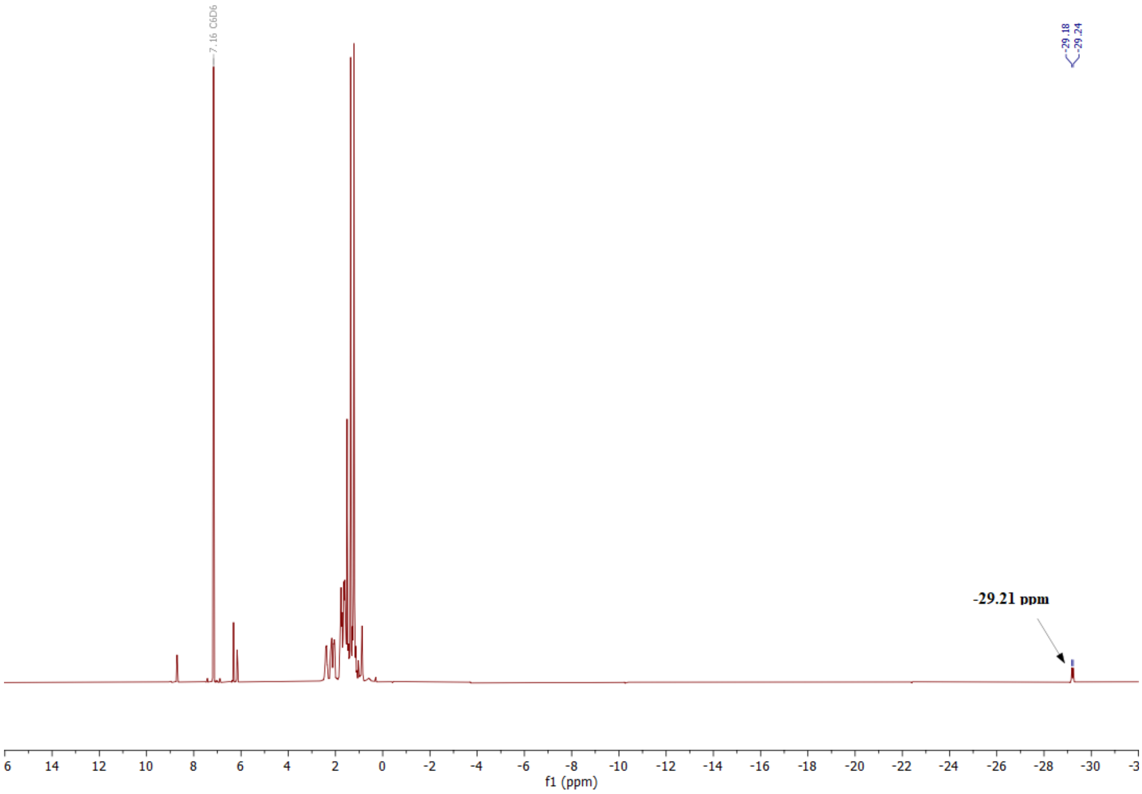

Figure S13. ^{1}H NMR spectrum of **3a** in C_6D_6 (300 MHz, 298K).

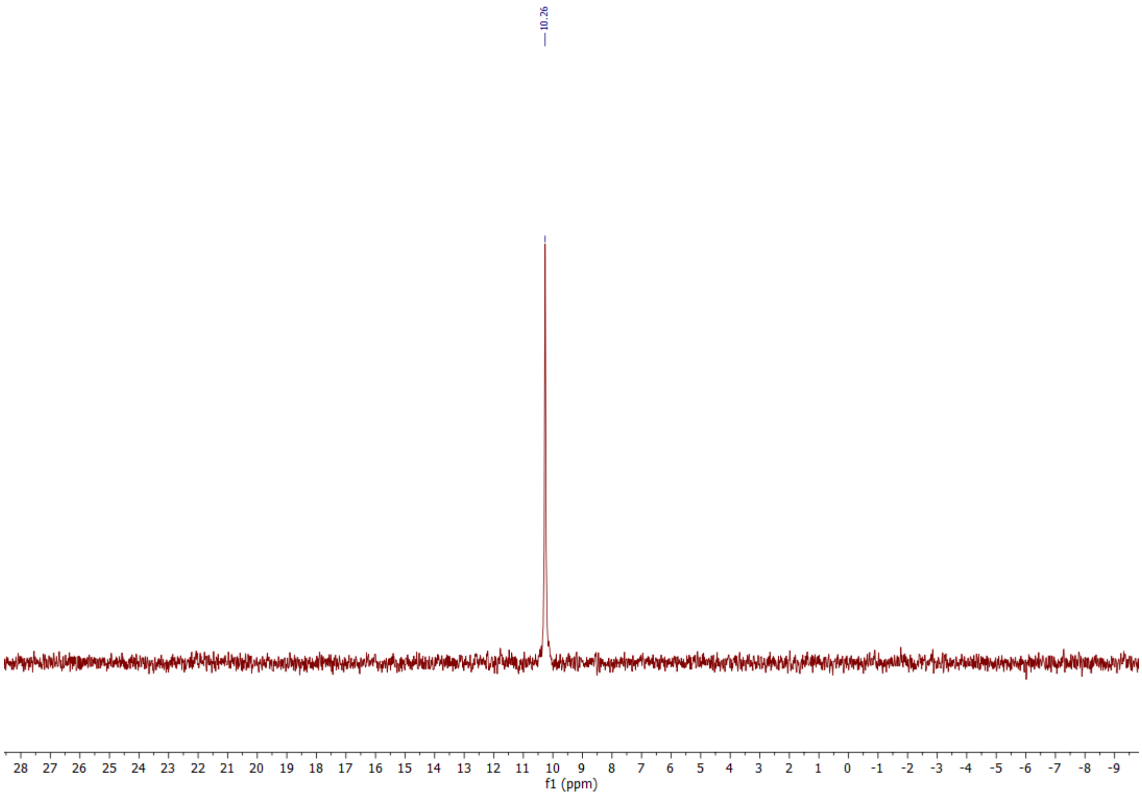

Figure S14. $^{31}P\{1H\}$ NMR spectrum of **3a** in C_6D_6 (121 MHz, 298K).

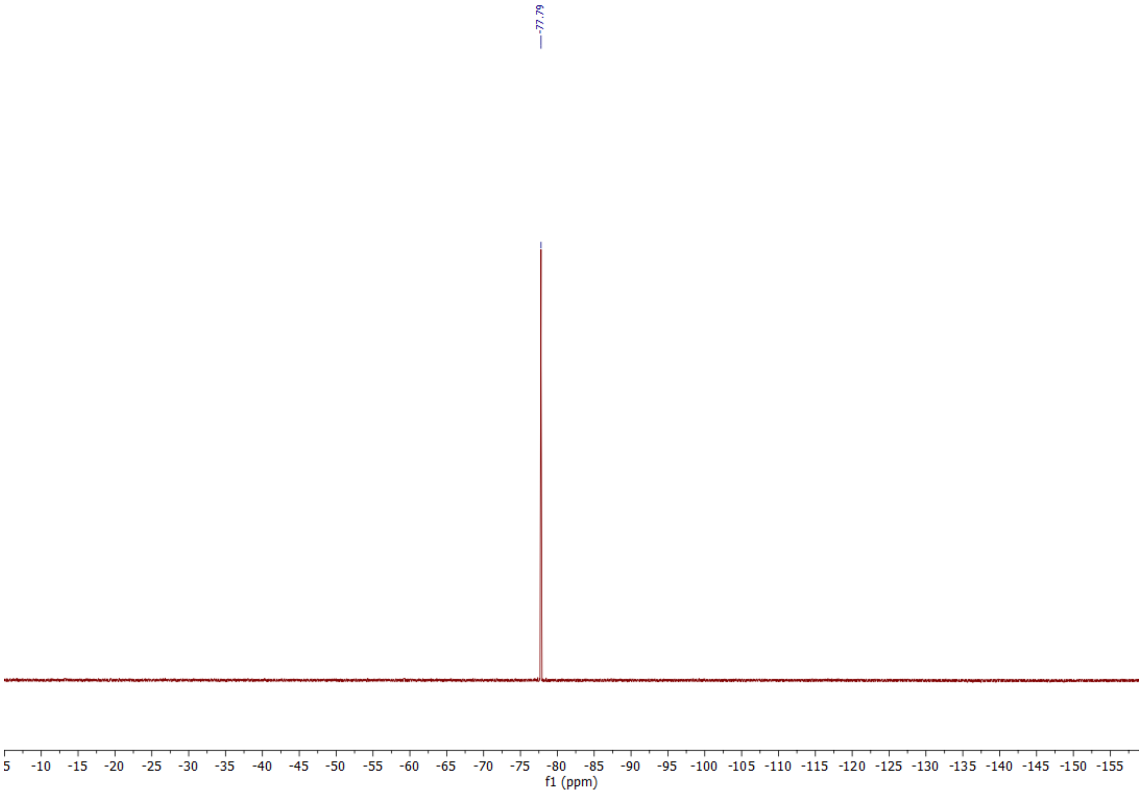

Figure S15. $^{19}F\{^{1}H\}$ NMR spectrum of **3a** in C_6D_6 (282 MHz, 298K).

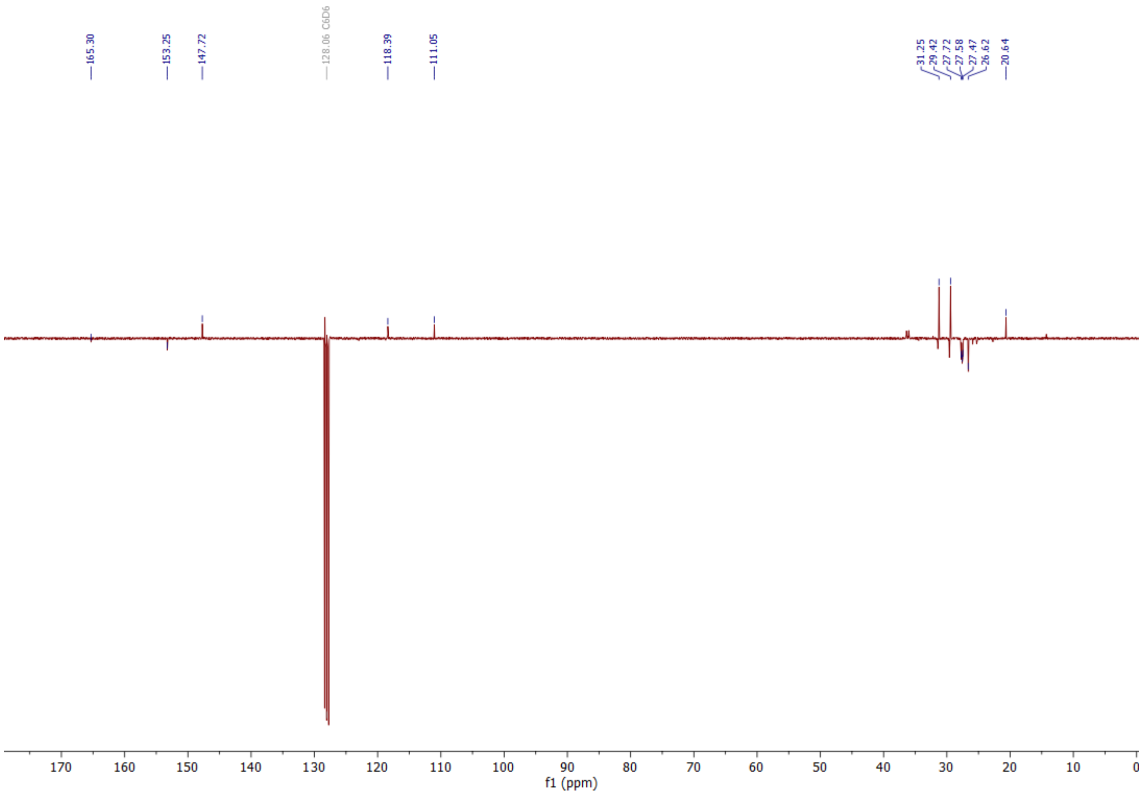

Figure S16. ^{13}C APT NMR spectrum of **3a** in C_6D_6 (75 MHz, 298K).

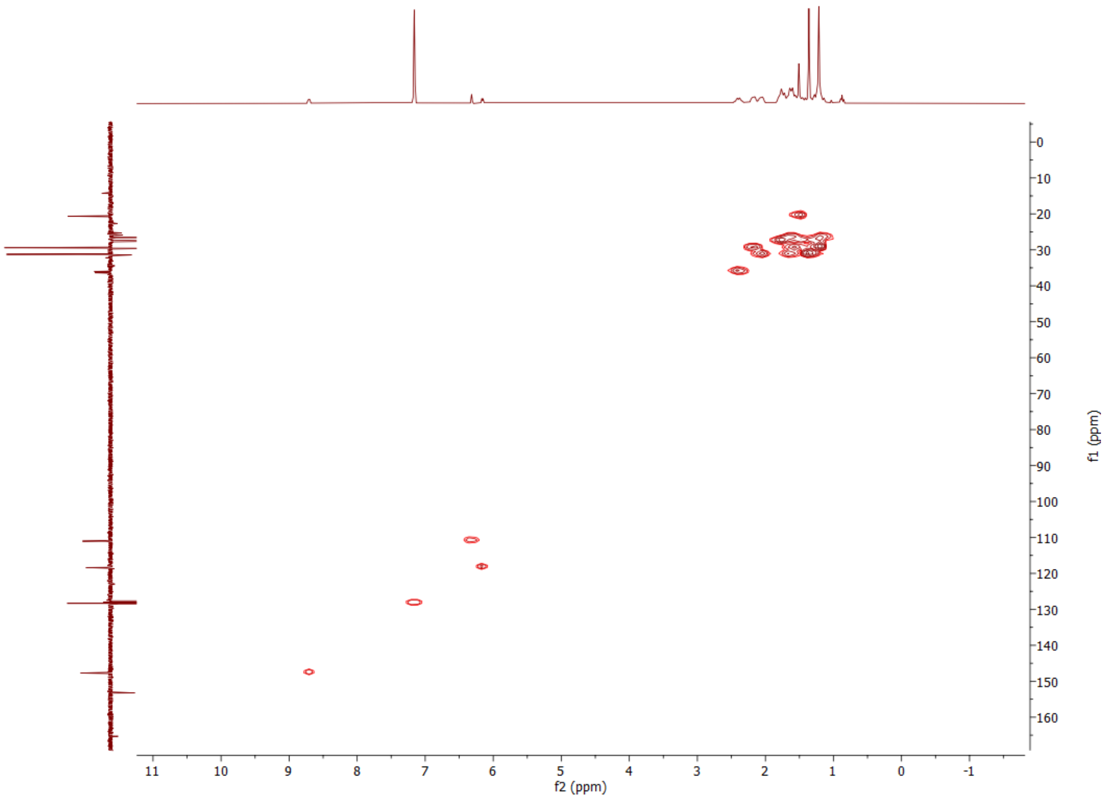

Figure S17. ^{1}H–^{13}C HSQC NMR spectrum of **3a** in C_6D_6 (298K).

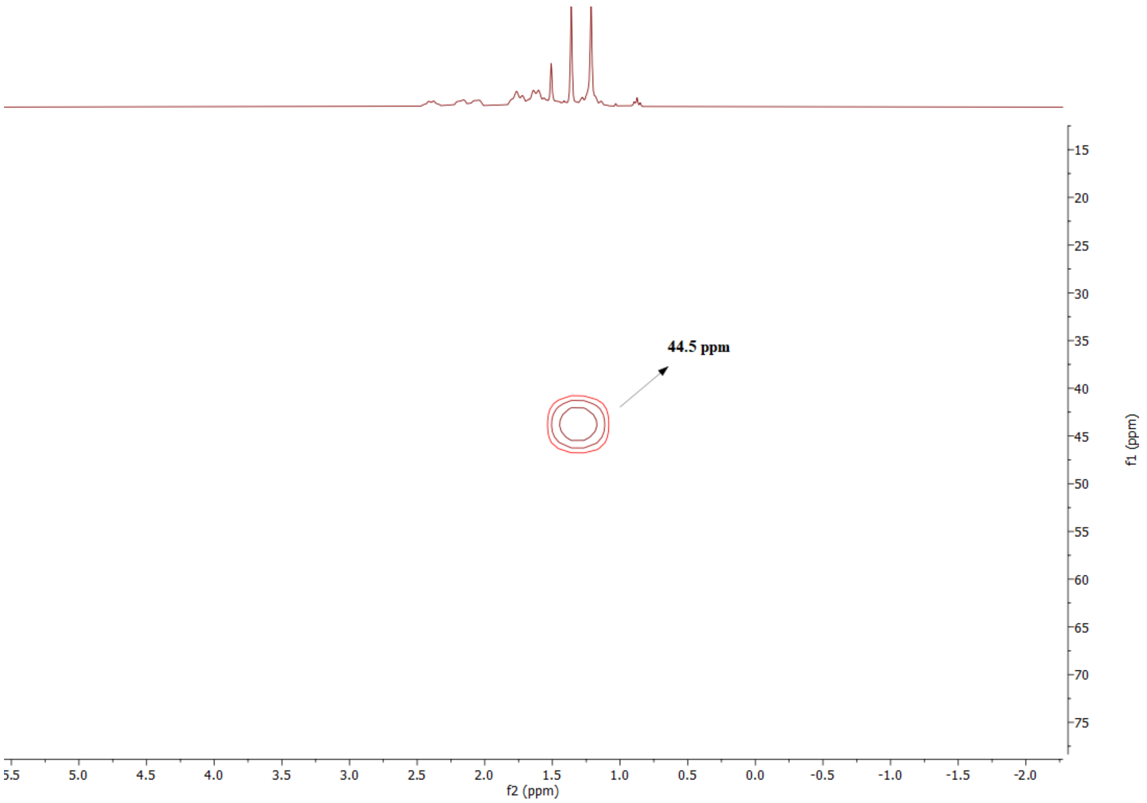

Figure S18. ^{1}H–^{29}Si HMBC spectrum of **3a** in C_6D_6 (60 MHz, 298K).

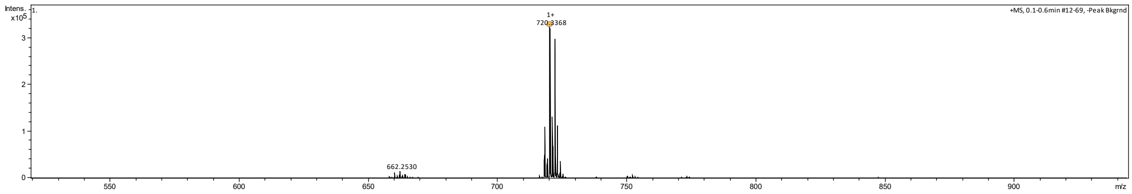

Figure S19. HR-MS of complex **3a.**

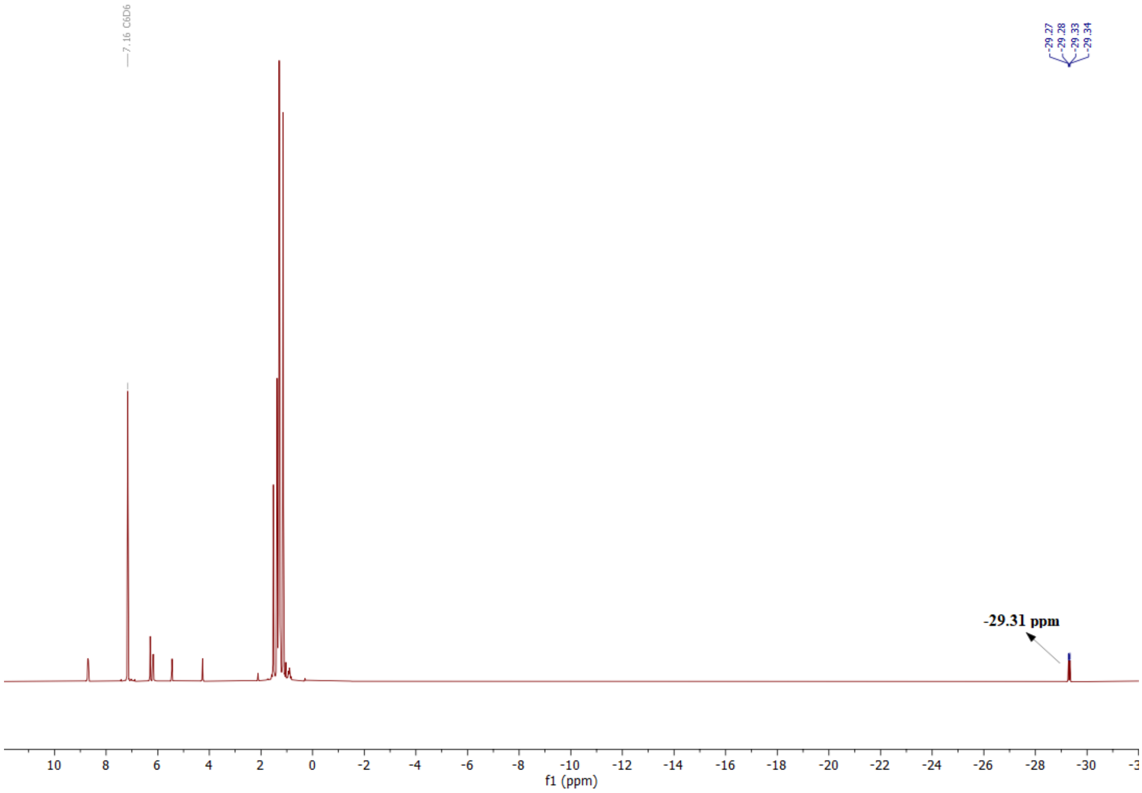

Figure S20. ^{1}H NMR spectrum of **3b** in C_6D_6 (300 MHz, 298K).

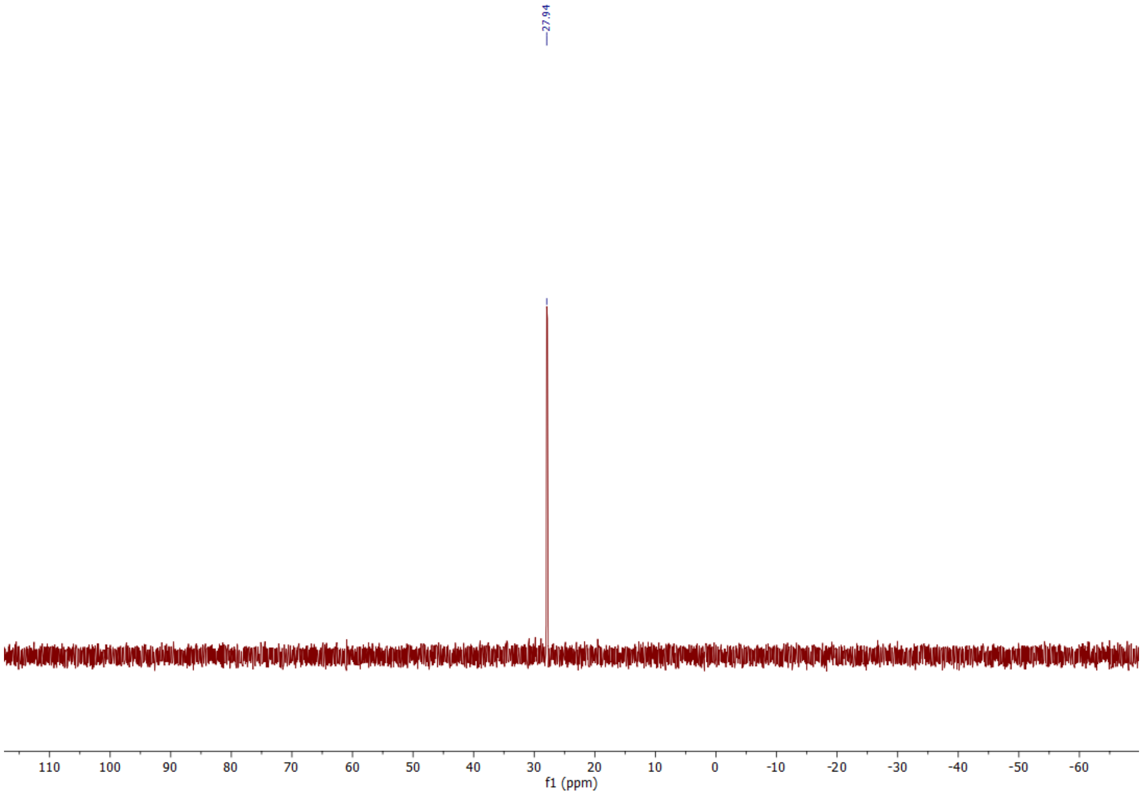

Figure S21. $^{31}P\{1H\}$ NMR spectrum of **3b** in C_6D_6 (121 MHz, 298K).

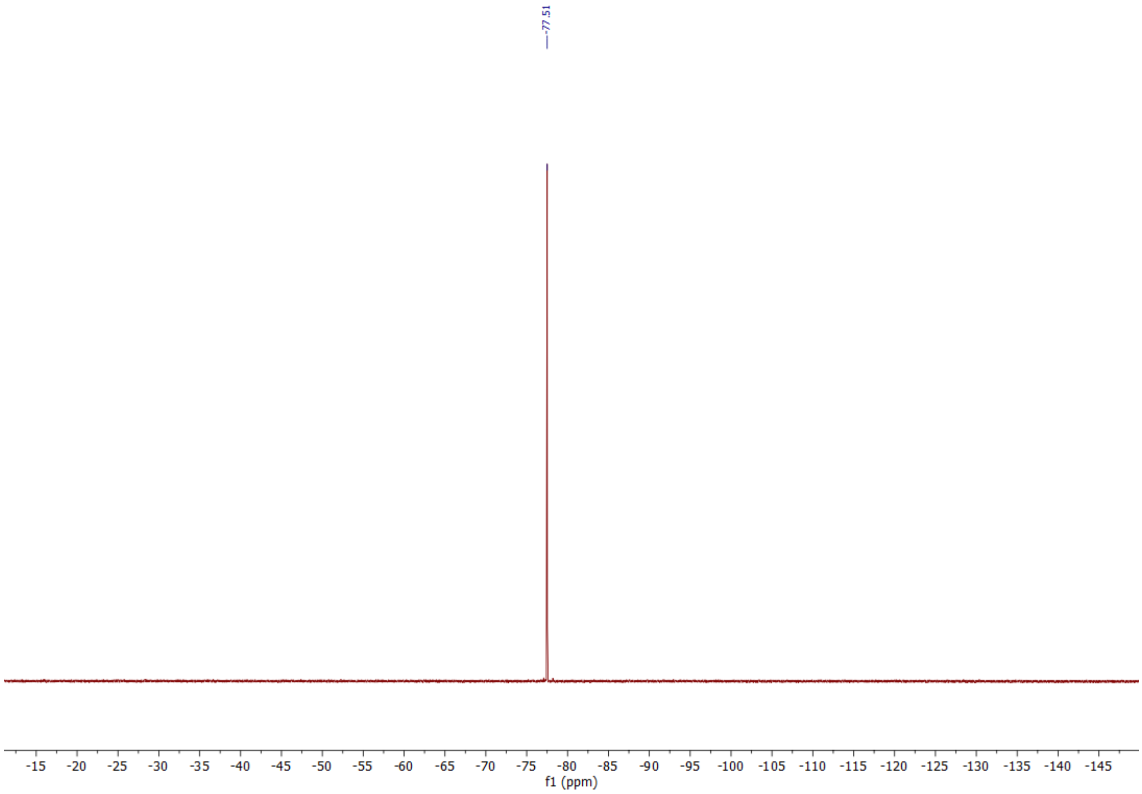

Figure S22. $^{19}F\{^1H\}$ NMR spectrum of **3b** in C_6D_6 (282 MHz, 298K).

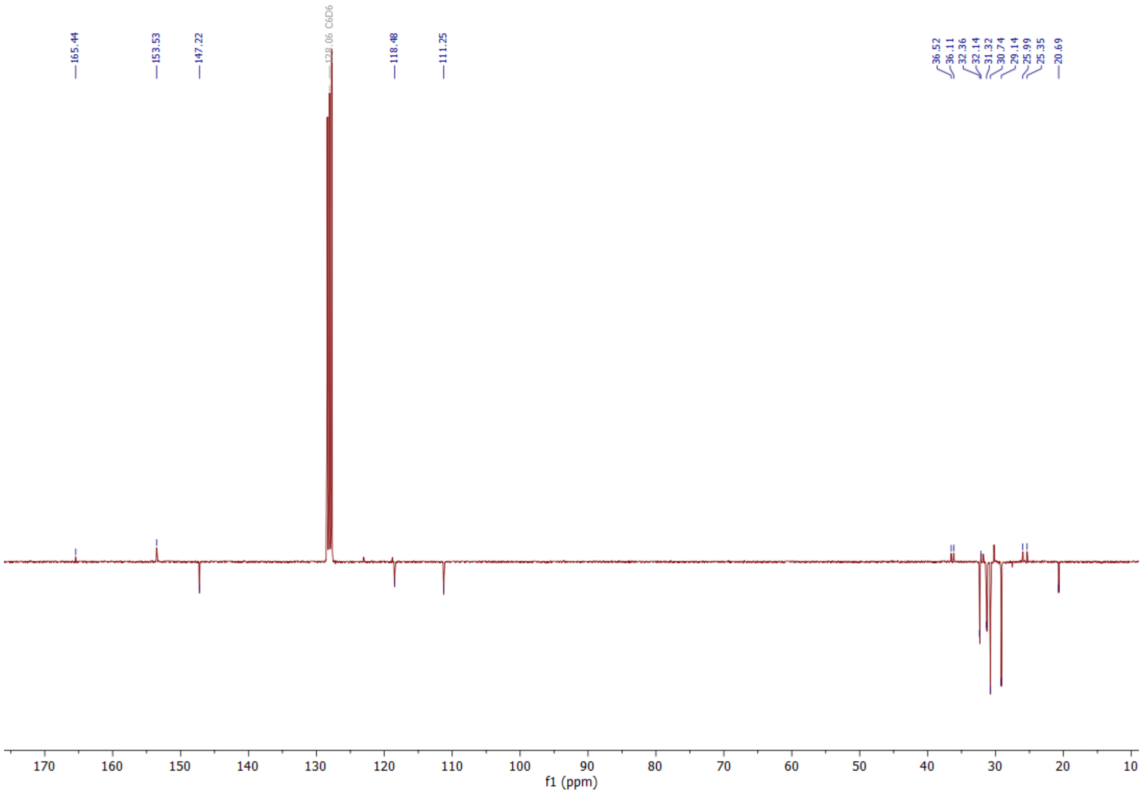

Figure S23. ^{13}C APT NMR spectrum of **3b** in C_6D_6 (75 MHz, 298K).

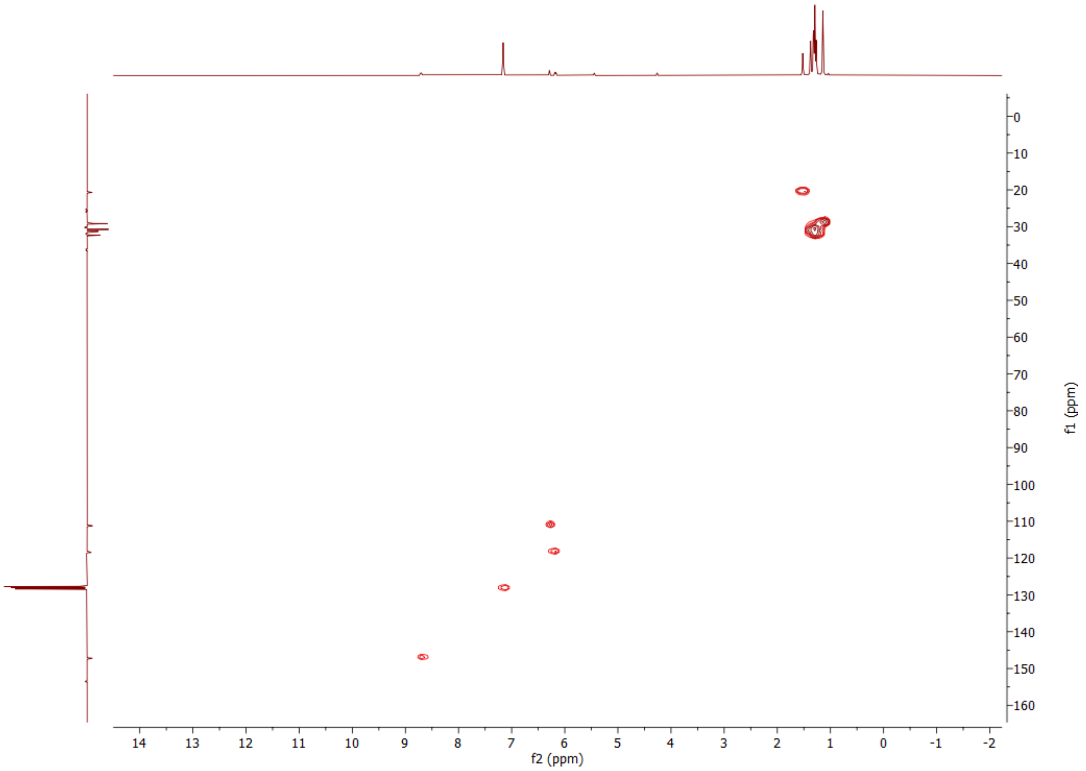

Figure S24. ^{1}H–^{13}C HSQC NMR spectrum of **3b** in C_6D_6 (298K).

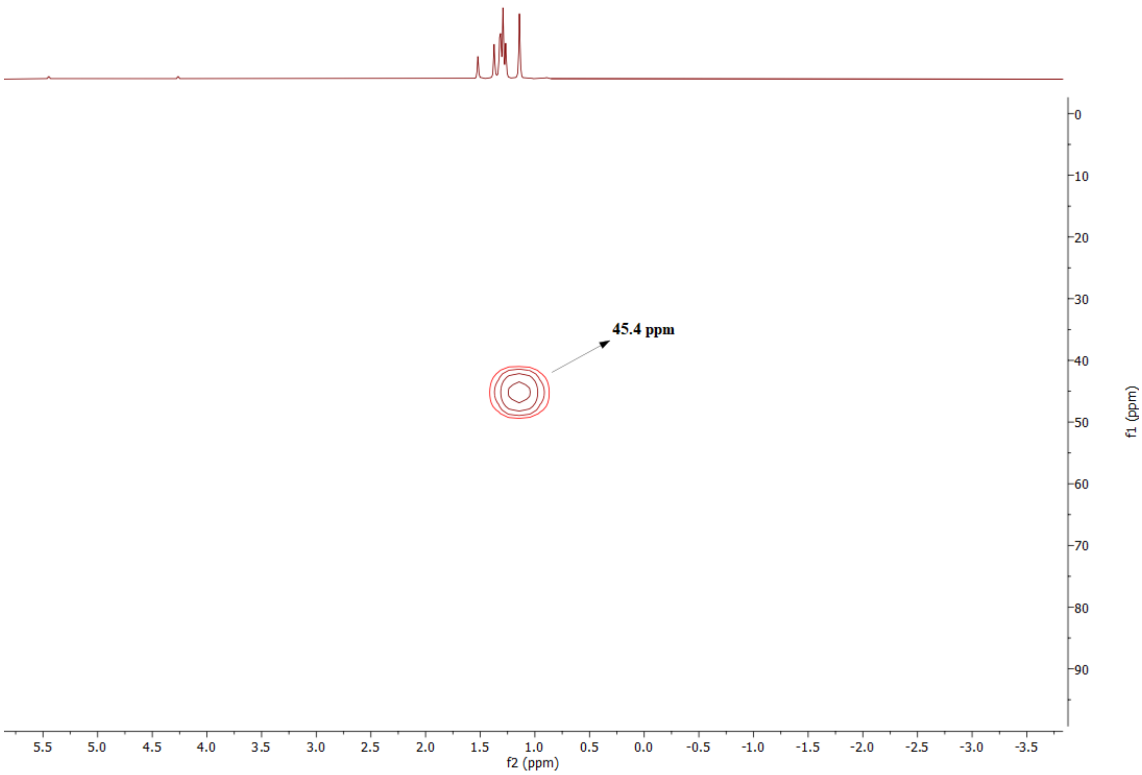

Figure S25. ^{1}H–^{29}Si HMBC spectrum of **3b** in C_6D_6 (60 MHz, 298K).

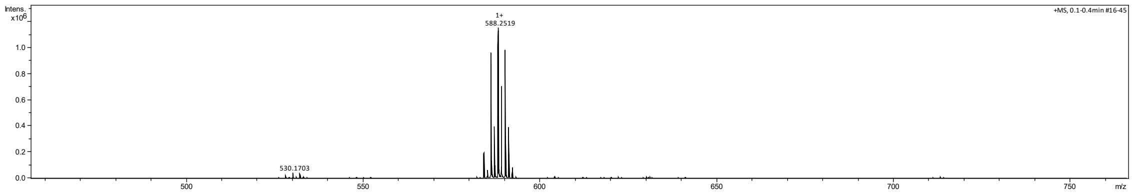

Figure S26. HR-MS of complex **3b.**

1. ^{1}H NMR Studies of the complexes 3a-H_2O, 3b-H_2O y 4-H_2O in CD_2Cl_2 (298K)

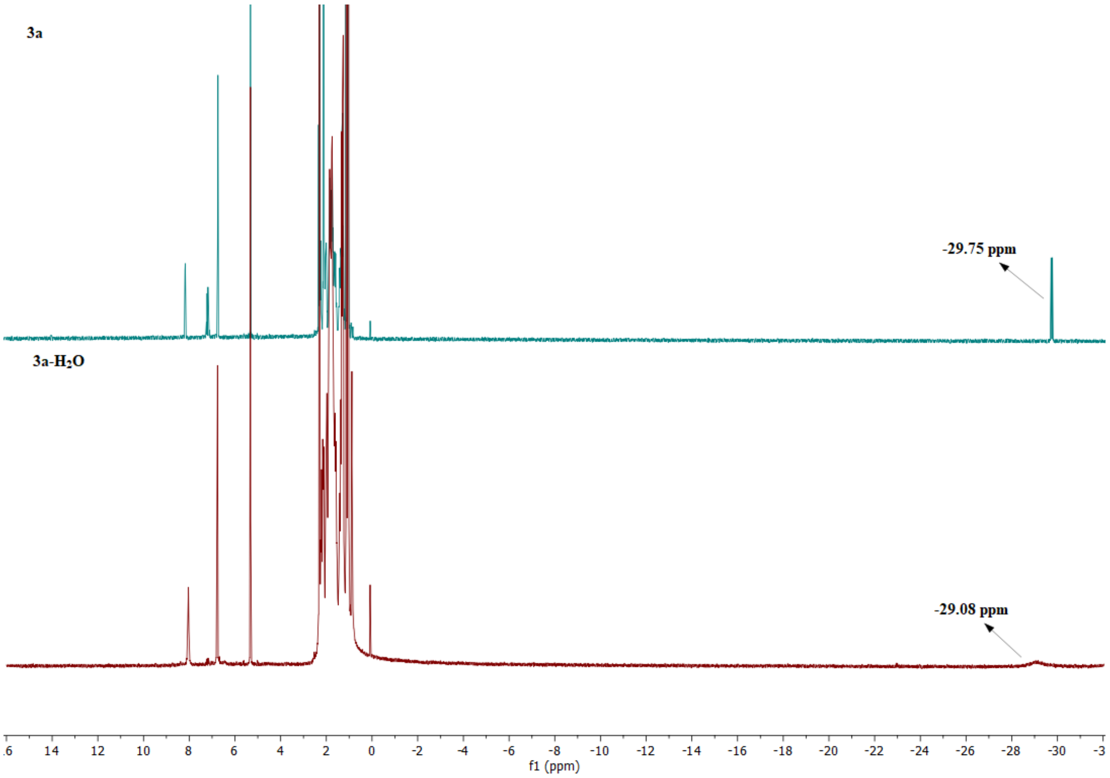

Figure S27. ^{1}H spectra of complexes **3a** y **3a-H_2O**.

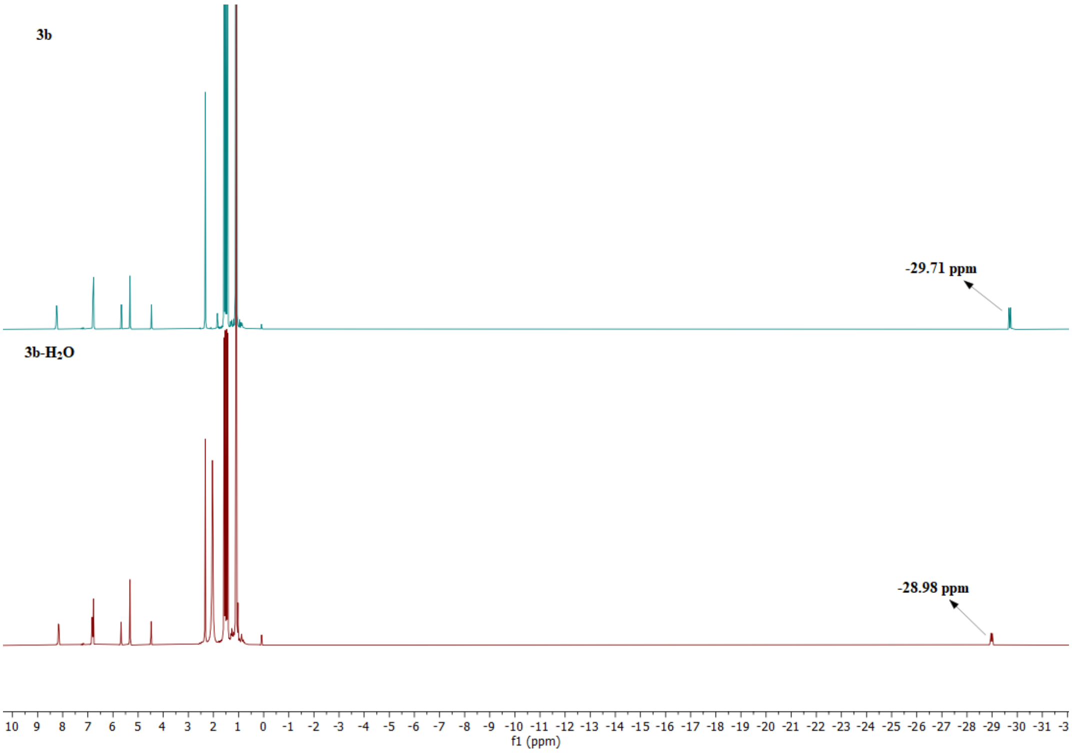

Figure S28. ^{1}H spectra of complexes **3b** y **3b-H_2O**.

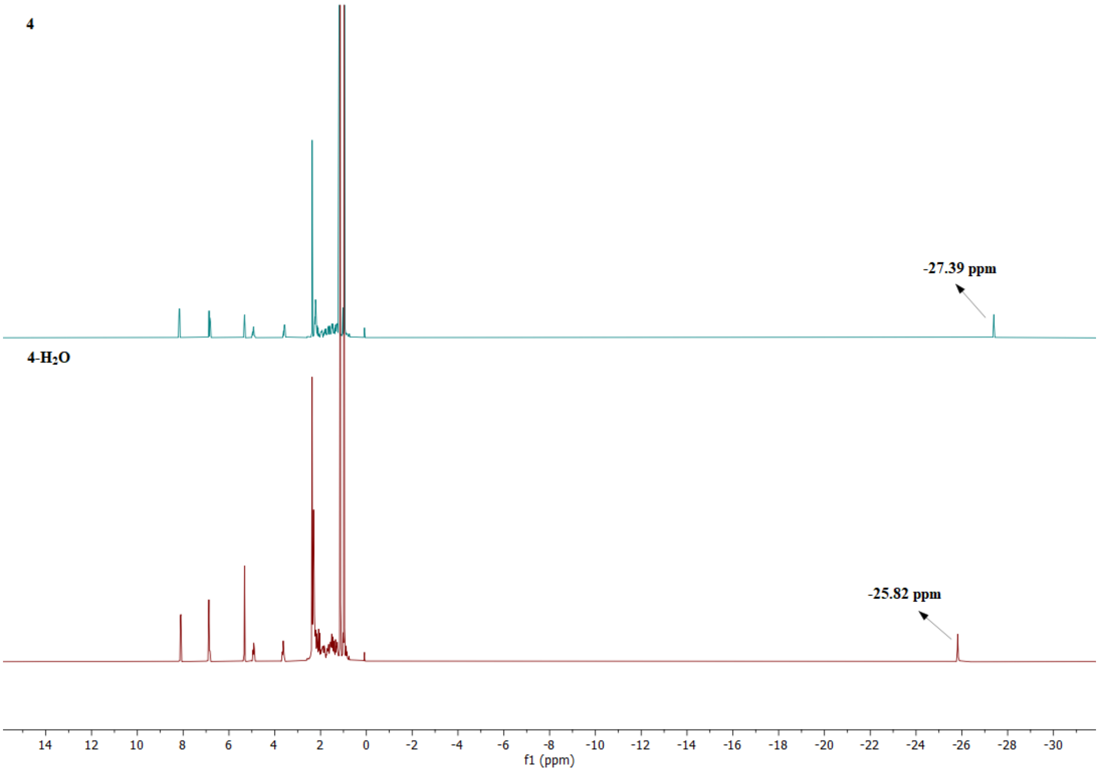

Figure S29. ^{1}H spectra of complexes **4** y **4-H_2O**.

2. VT NMR Studies (from 298 K to 193 K) of CD_2Cl_2 solutions of 3a-H_2O, 3b-H_2O and 4-H_2O

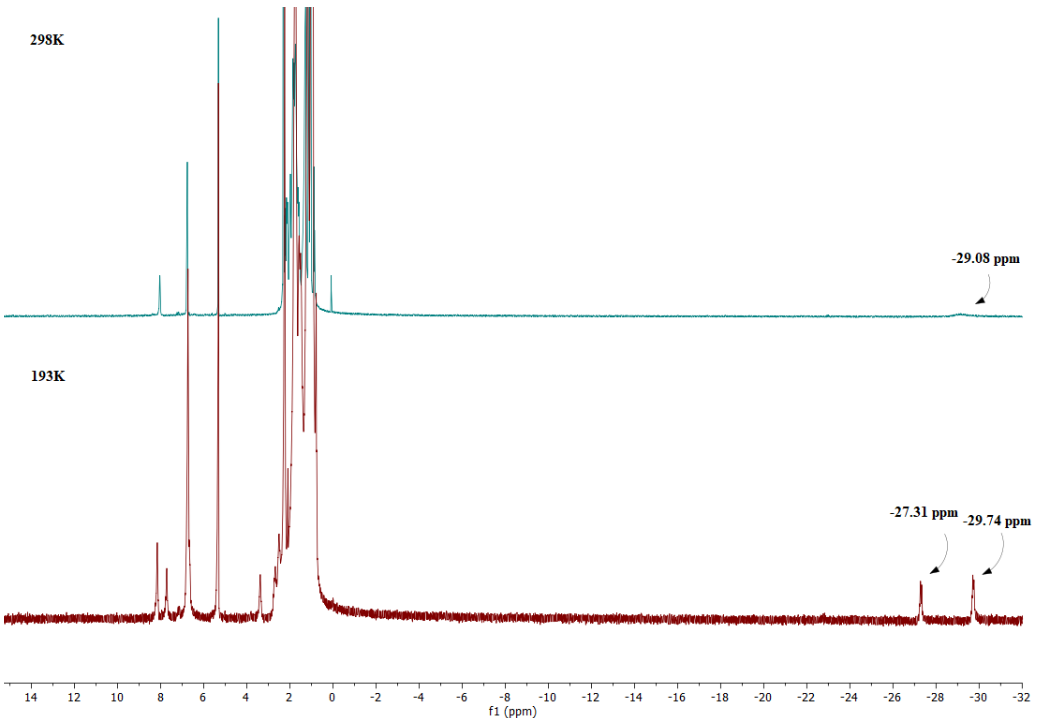

Figure S30. ^{1}H NMR spectrum of **3a-H_20** at 193 K and 298K.

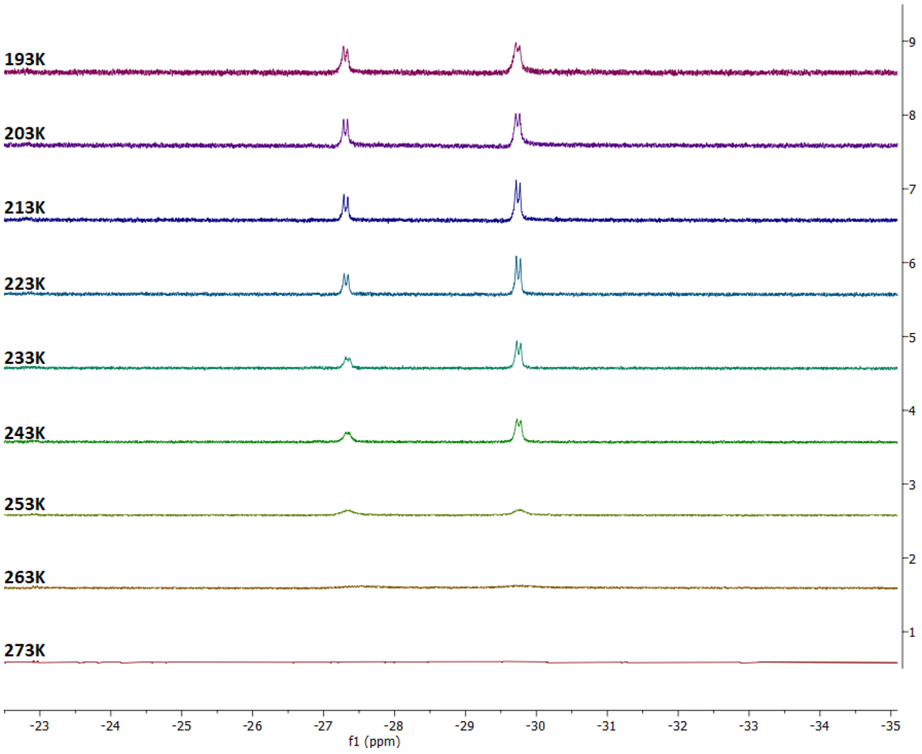

Figure S31. ^{1}H NMR spectrum of **3a-H_20** from 273K to 193K.

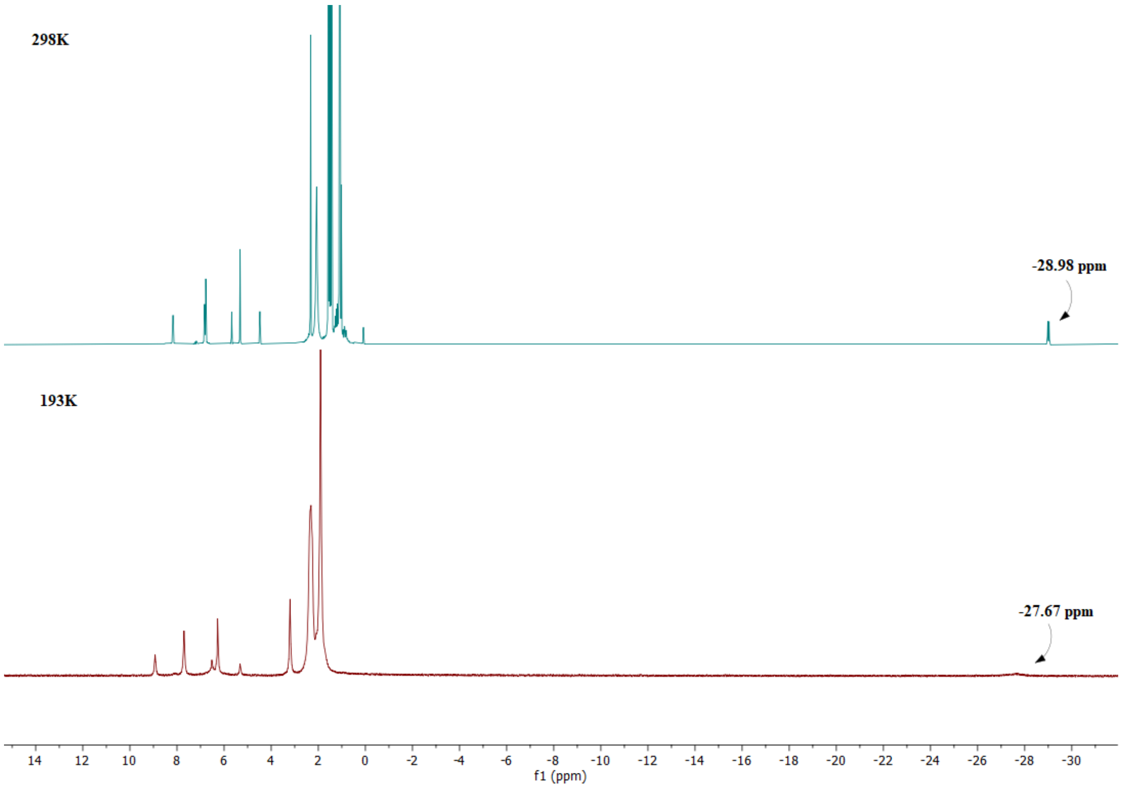

Figure S32. ^{1}H NMR spectrum of **3b-H₂0** at 193 K and 298K.

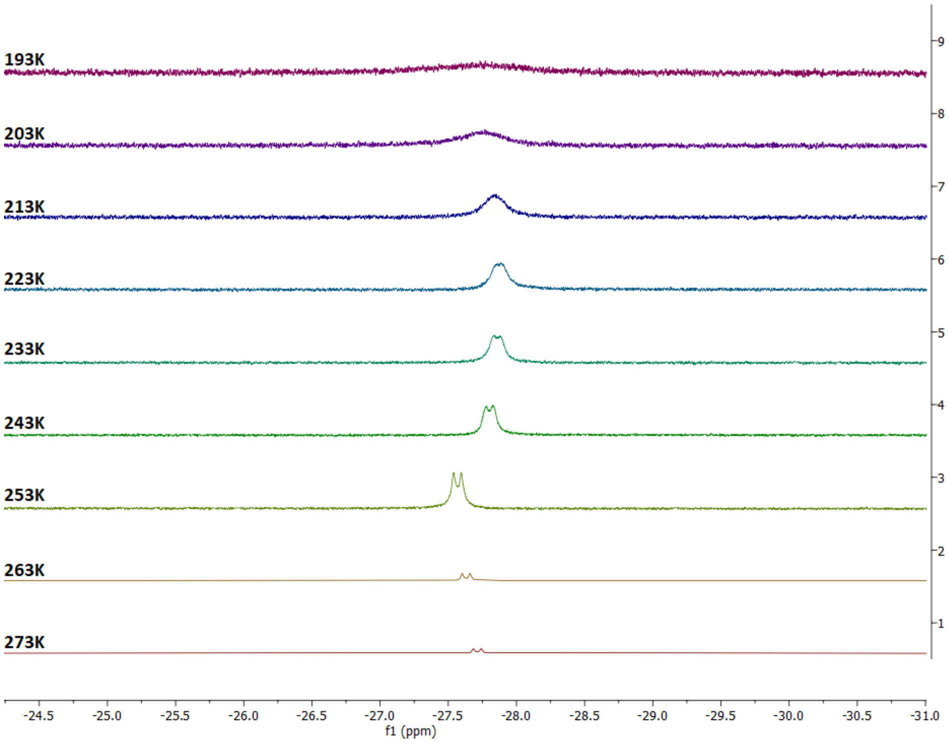

Figure S33. ^{1}H NMR spectrum of **3b-H₂0** from 273K to 193K.

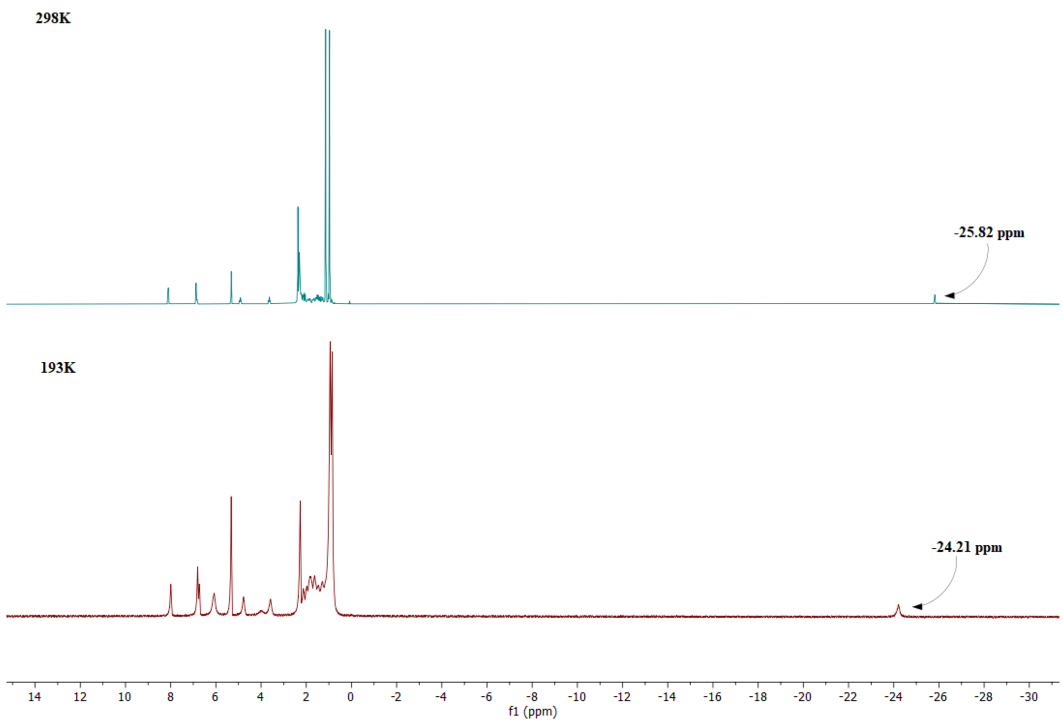

Figure S34. ^{1}H NMR spectrum of **4-H$_2$0** at 193 K and 298K.

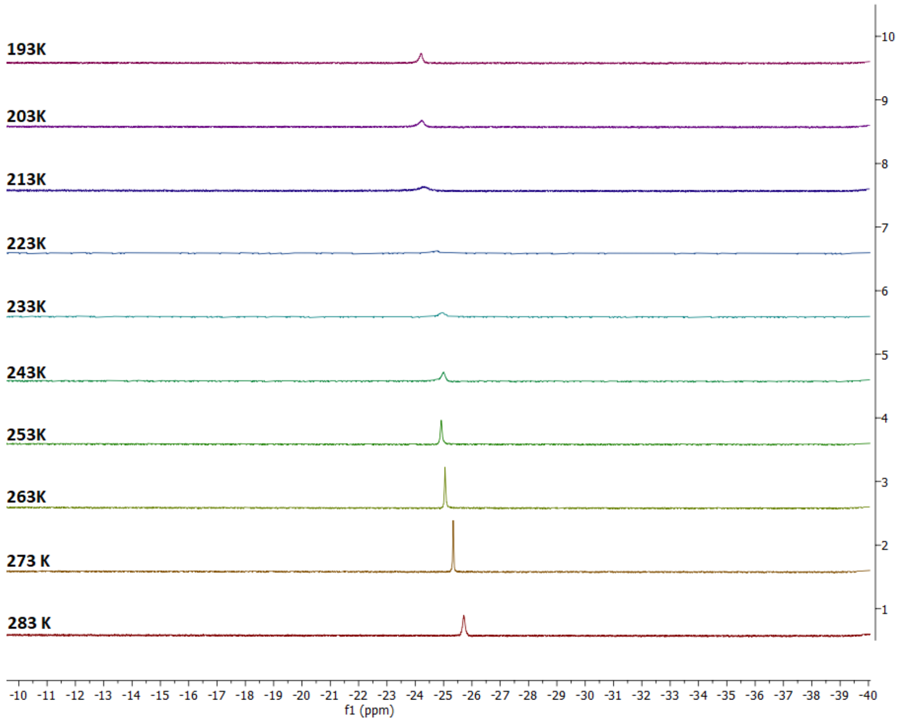

Figure S35. ^{1}H NMR spectrum of **4-H$_2$0** from 283K to 193K.

3. **Representation of H_2 evolution with the time**

Solventless reaction of $HSiMe(OSiMe_3)_2$ (1.0 mmol) with water using 3a (0.5 mol %) as catalyst at 323K.

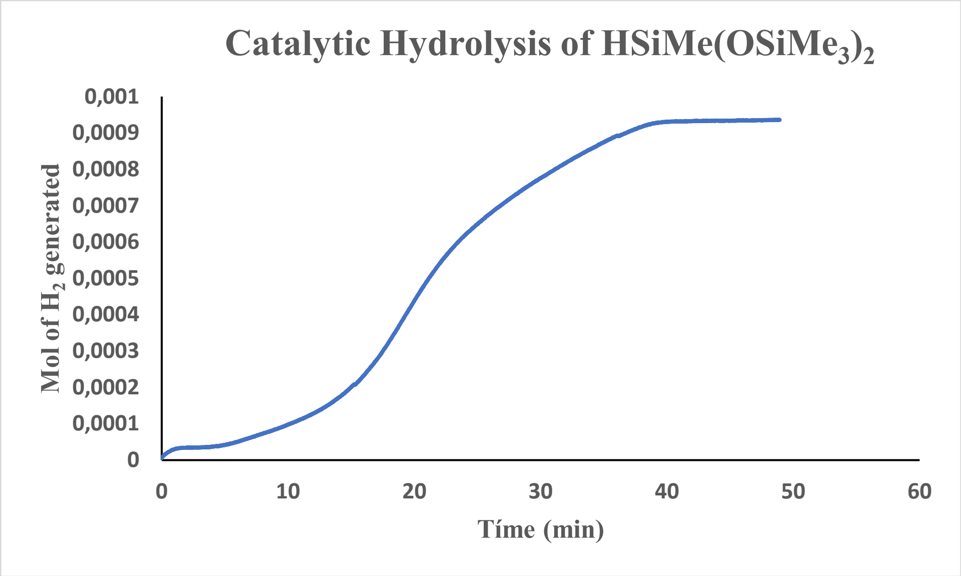

Figure S36. Representation of the amount of H2 (mol) released *versus* time (min).

Solventless reaction of $HSiMe(OSiMe_3)_2$ (1.0 mmol) with water using 3b (0.5 mol %) as catalyst at 323K.

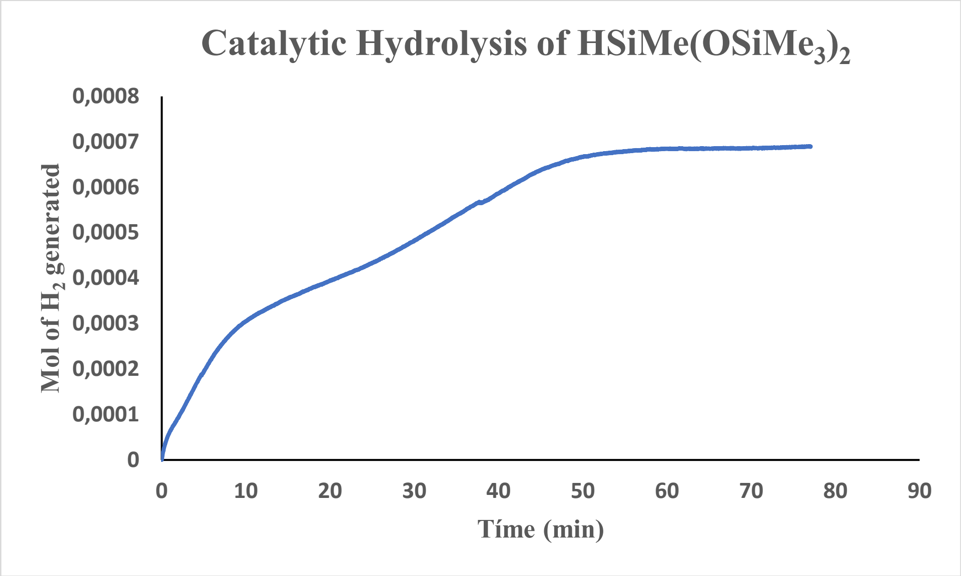

Figure S37. Representation of the amount of H2 (mol) released *versus* time (min).

Solventless reaction of $HSiMe(OSiMe_3)_2$ (1.0 mmol) with water using 4 (0.5 mol %) as catalyst at 323K.

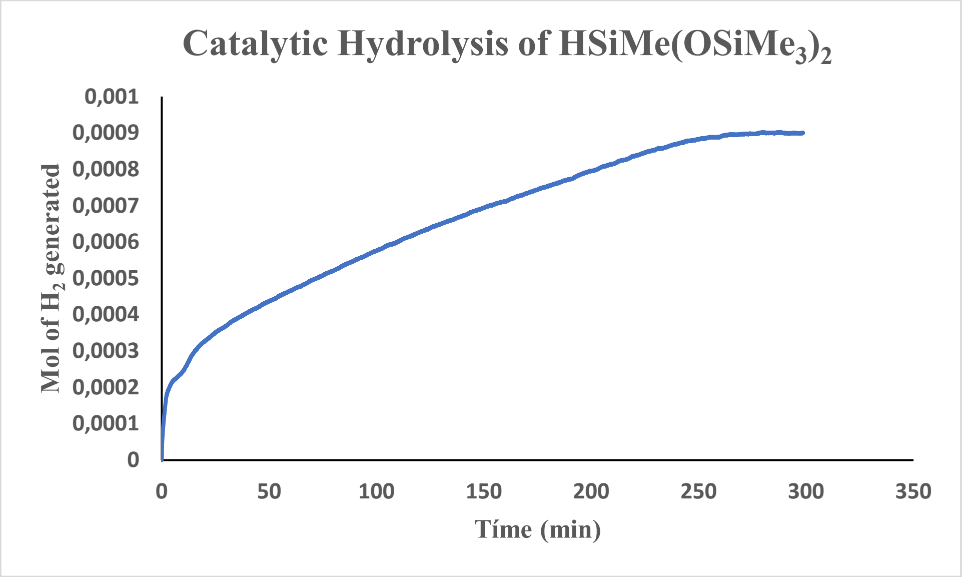

Figure S38. Representation of the amount of H2 (mol) released *versus* time (min).

Solventless reaction of $HSiMe(OSiMe_3)_2$ (1.0 mmol) with water using 3a (0.5 mol %) as catalyst at 298K.

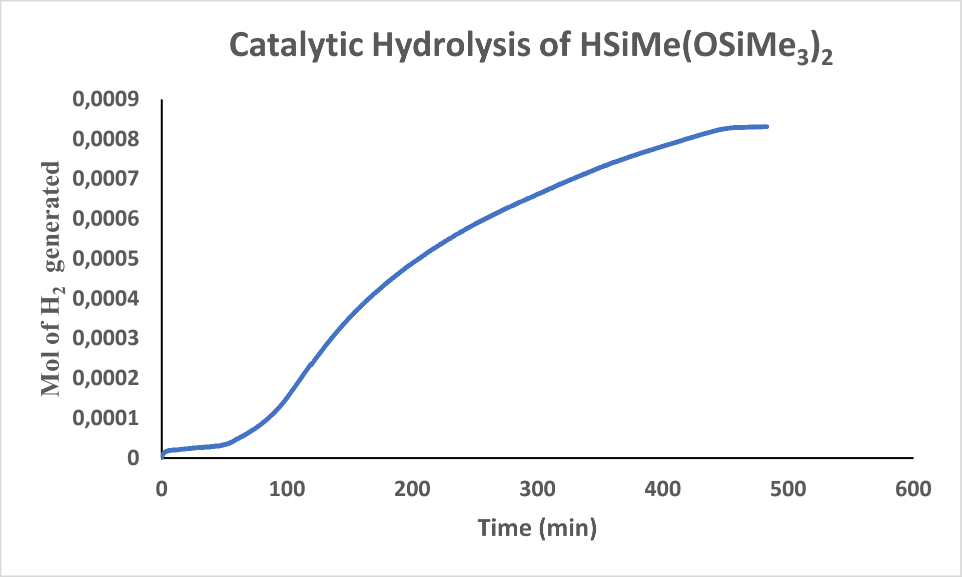

Figure S39. Representation of the amount of H2 (mol) released *versus* time (min).

Solventless reaction of HSiMe(OSiMe$_3$)$_2$ (1.0 mmol) with water using 3a (0.5 mol %) as catalyst at 353K.

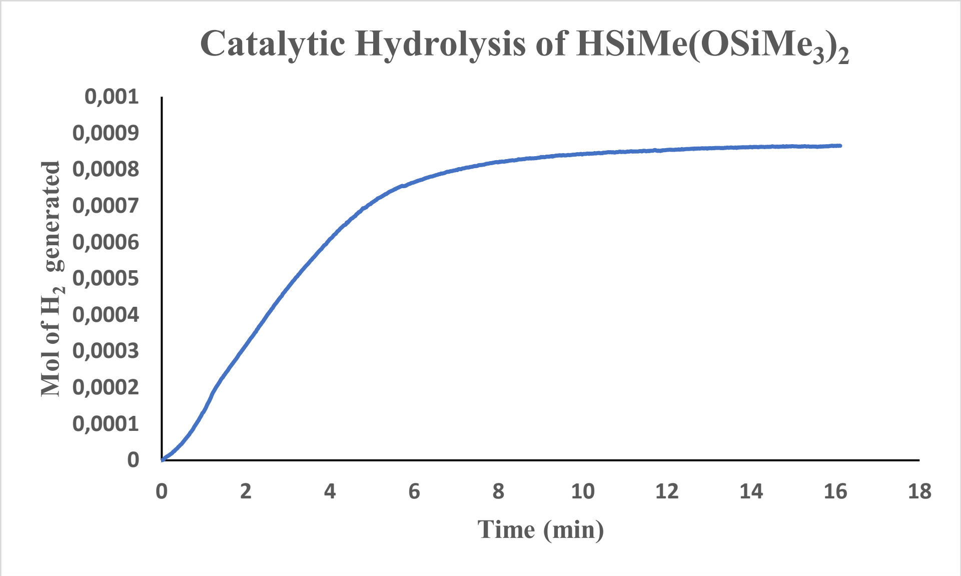

Figure S40. Representation of the amount of H2 (mol) released *versus* time (min).

Solventless reaction of extra HSiMe(OSiMe$_3$)$_2$ (1.0 mmol) with water using 3a (0.5 mol %) as catalyst at 323K.

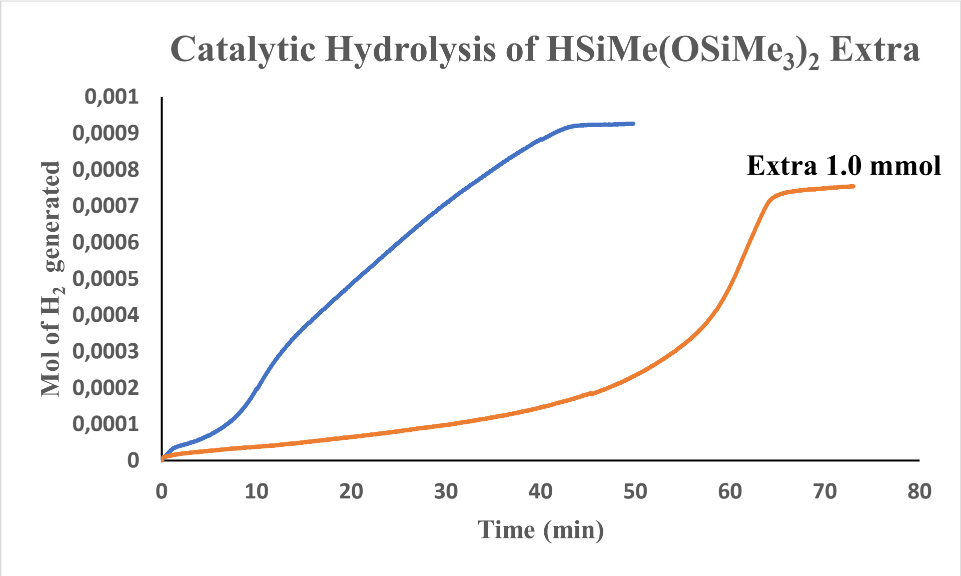

Figure S41. Representation of the amount of H2 (mol) released with extra 1.0 mmol of HSiMe(OSiMe$_3$)$_2$ *versus* time (min).

4. NMR studies of the products of the catalytic reactions

A NMR tube containing the finish product of the reaction in the micro-reactor and was analysed in CD_2Cl_2 by ^1H, 29Si{^1H} NMR spectroscopy.

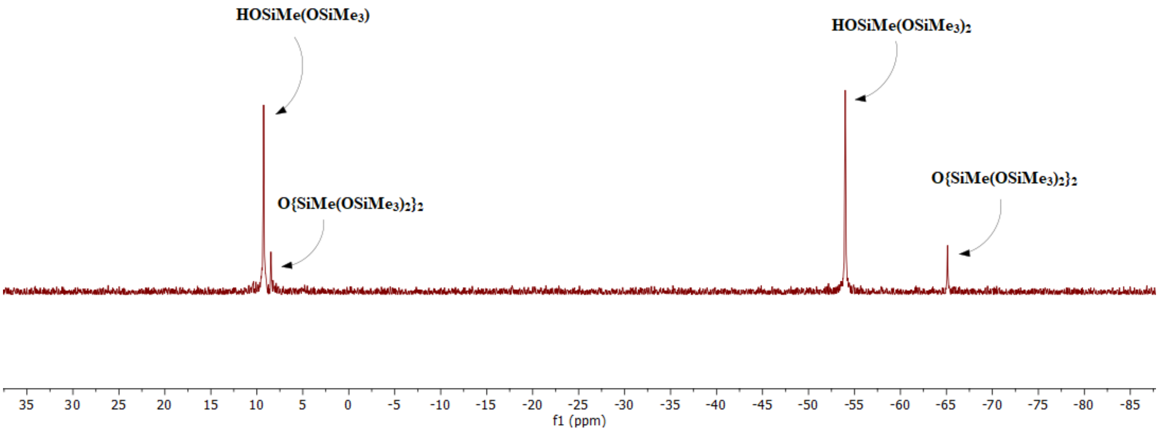

Figure S42. ^{29}Si DEP45 spectrum of solventless reaction of $HSiMe(OSiMe_3)_2$ (1.0 mmol) with water using **3a** (0.5 mol %) as catalyst at 353K.

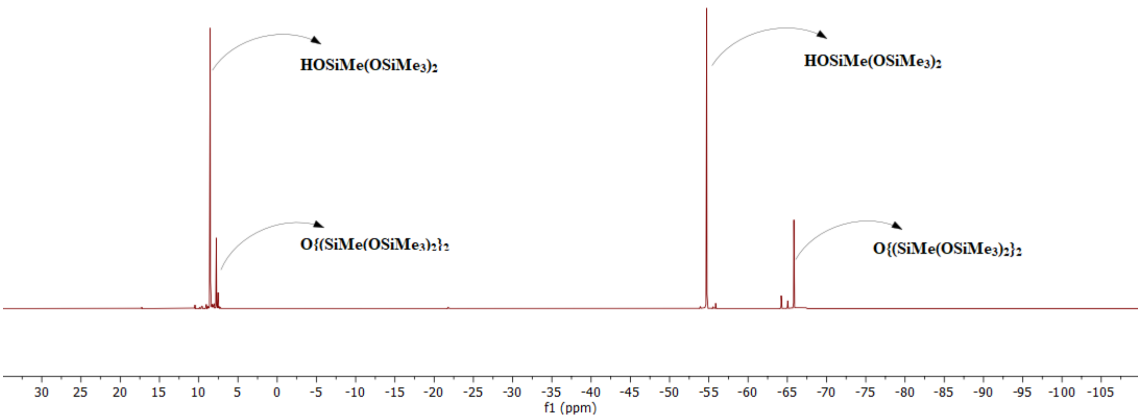

Figure S43. ^{29}Si DEP45 spectrum of solvent-less reaction of $HSiMe(OSiMe_3)_2$ (1.0 mmol) with water using **3b** (0.5 mol %) as catalyst at 323K.

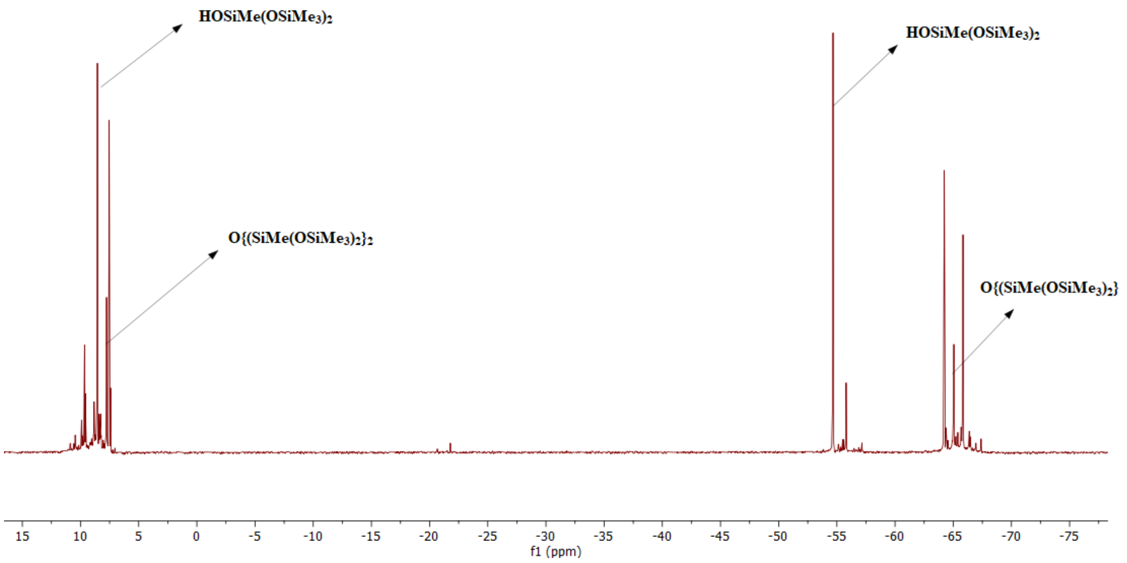

Figure S44. ^{29}Si DEP45 spectrum of solvent-less reaction of $HSiMe(OSiMe_3)_2$ (1.0 mmol) with water using **4** (0.5 mol %) as catalyst at 323K.

2. Computational Details

Cartesian coordinates (in Å) and total energies (in a. u., ZPVE included) of all the stationary points described in the text (BP86-D3/def2-SVP).

1: E= -1844.826634

Ir	-0.227704000	-0.577113000	-0.062400000
H	-0.001994000	-0.214207000	-1.592045000
Cl	-0.203168000	-1.766991000	2.062429000
Si	0.770422000	1.483884000	-0.081317000
O	2.392266000	1.005959000	-0.663065000
N	1.803183000	-1.194906000	-0.219654000
C	2.182583000	-2.490952000	-0.050226000
H	1.381407000	-3.174767000	0.266113000
C	3.493564000	-2.915668000	-0.232431000
H	3.742841000	-3.977219000	-0.087143000
C	4.492636000	-1.973981000	-0.592609000
C	4.102226000	-0.638887000	-0.740319000
H	4.819192000	0.153957000	-0.996851000
C	2.752218000	-0.262959000	-0.543484000
C	5.922705000	-2.408913000	-0.792760000
H	5.992975000	-3.162122000	-1.606305000
H	6.584472000	-1.559127000	-1.049819000
H	6.318103000	-2.892306000	0.125760000
C	1.098730000	2.087237000	1.748611000
C	2.096898000	1.104545000	2.405478000
H	2.275916000	1.413066000	3.460553000
H	3.080408000	1.107361000	1.890601000
H	1.706206000	0.066597000	2.428227000
C	1.707715000	3.505672000	1.788777000
H	1.006382000	4.280836000	1.416382000
H	2.649011000	3.577832000	1.204832000

H	1.952351000	3.770970000	2.842958000
C	-0.223300000	2.068563000	2.549625000
H	-0.625681000	1.038699000	2.633112000
H	-1.003711000	2.719324000	2.098739000
H	-0.040054000	2.445593000	3.581669000
C	0.283138000	2.836391000	-1.380519000
C	1.415331000	3.881828000	-1.538735000
H	1.158420000	4.582395000	-2.365697000
H	2.381630000	3.400434000	-1.795329000
H	1.566725000	4.492918000	-0.630150000
C	-1.021306000	3.529109000	-0.927515000
H	-1.339966000	4.286671000	-1.679383000
H	-0.899486000	4.059782000	0.039994000
H	-1.860266000	2.810403000	-0.810316000
C	0.074375000	2.173827000	-2.760887000
H	-0.771274000	1.457551000	-2.767742000
H	0.980980000	1.624863000	-3.091153000
H	-0.144140000	2.955089000	-3.523942000
C	-2.171682000	-0.214448000	-0.938005000
C	-2.266700000	0.073427000	0.455458000
C	-3.110264000	-0.749113000	1.405971000
H	-2.688978000	-0.701819000	2.429818000
H	-3.067817000	-1.822758000	1.129851000
C	-4.571612000	-0.249710000	1.416941000
H	-5.165962000	-0.939024000	2.058704000
H	-4.613290000	0.743106000	1.919940000
C	-5.253241000	-0.124998000	0.039946000
H	-4.796304000	0.717249000	-0.526770000
H	-6.307300000	0.181172000	0.215597000
C	-5.231849000	-1.399024000	-0.843637000
H	-5.096544000	-2.300556000	-0.203081000
H	-6.230415000	-1.524716000	-1.315764000
C	-4.190836000	-1.421550000	-1.981080000
H	-4.341082000	-0.525077000	-2.626482000
H	-4.400654000	-2.299245000	-2.631735000

C	-2.702437000	-1.492925000	-1.566920000
H	-2.554195000	-2.354666000	-0.879163000
H	-2.092798000	-1.710163000	-2.470575000
H	-2.156560000	0.642426000	-1.635901000
H	-2.231118000	1.143495000	0.736467000

4: E= -2345.635825

Ir	-0.131841000	-0.308839000	-0.679016000
H	-1.200423000	-0.903720000	-1.663522000
O	1.674700000	0.724857000	-0.068457000
Si	-2.118585000	-0.880477000	0.350669000
O	-3.011900000	0.630318000	0.031937000
N	-1.053136000	1.568394000	-0.783881000
C	-0.378058000	2.669350000	-1.220820000
H	0.685421000	2.514614000	-1.447969000
C	-0.982194000	3.912958000	-1.342045000
H	-0.386889000	4.766793000	-1.696747000
C	-2.342519000	4.071454000	-0.973047000
C	-3.026004000	2.942531000	-0.506046000
H	-4.077405000	2.987029000	-0.188612000
C	-2.366109000	1.696657000	-0.415458000
C	-3.009522000	5.420336000	-1.060240000
H	-2.913092000	5.844031000	-2.082117000
H	-4.085090000	5.371411000	-0.801785000
H	-2.519856000	6.138344000	-0.367935000
C	-2.001544000	-0.885987000	2.294137000
C	-0.927185000	0.141144000	2.713466000
H	-0.911148000	0.241198000	3.822139000
H	-1.106707000	1.152235000	2.294376000
H	0.092526000	-0.156254000	2.403746000
C	-3.355435000	-0.440987000	2.900038000
H	-4.174377000	-1.162909000	2.716892000
H	-3.675056000	0.546224000	2.509737000
H	-3.243238000	-0.346009000	4.004045000

C	-1.623972000	-2.282010000	2.832681000
H	-0.640603000	-2.628385000	2.453902000
H	-2.381628000	-3.054346000	2.583245000
H	-1.546665000	-2.247329000	3.943153000
C	-3.272890000	-2.215301000	-0.452276000
C	-4.554312000	-2.431290000	0.384362000
H	-5.229287000	-3.144843000	-0.140820000
H	-5.120186000	-1.487889000	0.531143000
H	-4.337957000	-2.864532000	1.382165000
C	-2.512615000	-3.553673000	-0.584461000
H	-3.167796000	-4.320982000	-1.056023000
H	-2.191343000	-3.957609000	0.398381000
H	-1.612576000	-3.455817000	-1.226491000
C	-3.684663000	-1.722914000	-1.858307000
H	-2.806794000	-1.578123000	-2.524477000
H	-4.235043000	-0.761044000	-1.812585000
H	-4.345410000	-2.475449000	-2.345014000
C	0.952979000	-2.076605000	-1.353553000
C	0.799410000	-2.179479000	0.052904000
C	1.947431000	-2.048101000	1.027484000
H	1.595969000	-1.632684000	1.993714000
H	2.692295000	-1.318905000	0.659895000
C	2.604637000	-3.425837000	1.263740000
H	3.498132000	-3.268570000	1.908125000
H	1.913755000	-4.071050000	1.853107000
C	3.019017000	-4.190139000	-0.010074000
H	2.109569000	-4.545769000	-0.545095000
H	3.543393000	-5.114835000	0.314571000
C	3.919886000	-3.416769000	-1.008140000
H	4.455298000	-2.596146000	-0.478507000
H	4.718533000	-4.100655000	-1.368782000
C	3.223538000	-2.844200000	-2.261011000
H	2.687812000	-3.667814000	-2.787657000
H	4.008141000	-2.494344000	-2.967600000
C	2.247750000	-1.663338000	-2.032634000

H	2.760543000	-0.871892000	-1.448215000
H	1.995698000	-1.213277000	-3.017914000
H	0.292554000	-2.699502000	-1.986273000
H	0.007275000	-2.866996000	0.402653000
S	1.933897000	1.628279000	1.175200000
O	0.859278000	2.621599000	1.418055000
O	2.488683000	0.875484000	2.322359000
C	3.379528000	2.612784000	0.448501000
F	4.385711000	1.790396000	0.106139000
F	2.973808000	3.280047000	-0.656261000
F	3.825387000	3.502918000	1.346969000

3b: E= -2689.918453

Ir	0.227109000	-0.061031000	-0.428903000
H	1.148333000	-0.806468000	-1.449214000
O	-1.286432000	1.319550000	0.416557000
Si	2.135248000	-0.820856000	0.612405000
N	1.431461000	1.687085000	-0.647301000
O	3.246427000	0.500739000	0.171077000
P	-1.030983000	-1.994995000	-0.399421000
C	0.936660000	2.853328000	-1.150197000
C	1.733850000	3.971094000	-1.374050000
C	3.116900000	3.926049000	-1.068136000
C	3.619252000	2.731355000	-0.538795000
C	2.762510000	1.625862000	-0.333751000
C	4.000004000	5.125812000	-1.301006000
H	5.047473000	4.938547000	-0.994113000
H	3.998302000	5.410947000	-2.374723000
H	3.623725000	6.005464000	-0.736835000
C	2.040739000	-0.633850000	2.551446000
C	3.408564000	-0.215078000	3.140736000
H	3.776776000	0.727451000	2.687649000
H	4.194420000	-0.982847000	3.007553000
H	3.298310000	-0.043697000	4.236101000

C	1.589256000	-1.968954000	3.181038000
H	1.367452000	-1.834186000	4.264207000
H	2.374618000	-2.749911000	3.102773000
H	0.670015000	-2.371873000	2.705147000
C	1.027827000	0.489750000	2.876887000
H	0.003670000	0.261443000	2.529491000
H	1.316785000	1.453037000	2.406377000
H	0.988073000	0.654508000	3.977905000
C	3.181085000	-2.323229000	-0.043089000
C	4.501627000	-2.406621000	0.758283000
H	5.166456000	-3.176767000	0.304858000
H	4.330188000	-2.705531000	1.812377000
H	5.053195000	-1.444028000	0.754706000
C	2.441294000	-3.672377000	0.064237000
H	1.550635000	-3.705138000	-0.594180000
H	2.107861000	-3.902248000	1.097104000
H	3.113332000	-4.500267000	-0.258137000
C	3.525939000	-2.053727000	-1.525483000
H	4.160620000	-2.877715000	-1.923506000
H	4.083336000	-1.103140000	-1.651014000
H	2.618053000	-2.001444000	-2.161761000
C	-2.472648000	-2.248163000	0.860500000
C	-3.834108000	-1.793744000	0.301909000
H	-4.590707000	-1.866597000	1.112950000
H	-4.186464000	-2.433086000	-0.531753000
H	-3.809657000	-0.744586000	-0.046562000
C	-2.523788000	-3.735993000	1.274217000
H	-1.571727000	-4.069253000	1.739232000
H	-2.751057000	-4.413784000	0.429493000
H	-3.326672000	-3.870383000	2.031741000
C	-2.129647000	-1.402474000	2.103362000
H	-2.081988000	-0.325018000	1.864853000
H	-1.164802000	-1.705177000	2.555318000
H	-2.918516000	-1.552184000	2.871865000
C	-1.525001000	-2.547822000	-2.168943000

C	-0.210511000	-2.766417000	-2.953664000
H	-0.457966000	-3.131083000	-3.974221000
H	0.447933000	-3.524717000	-2.480426000
H	0.362818000	-1.824810000	-3.062044000
C	-2.329496000	-1.422675000	-2.848755000
H	-2.551444000	-1.715784000	-3.898181000
H	-1.754135000	-0.474091000	-2.871246000
H	-3.284492000	-1.201366000	-2.339116000
C	-2.310628000	-3.873044000	-2.165106000
H	-3.291274000	-3.788012000	-1.659104000
H	-1.736950000	-4.694421000	-1.686700000
H	-2.511425000	-4.176638000	-3.215739000
H	-0.265176000	-3.143835000	-0.029105000
H	-0.144132000	2.886618000	-1.349531000
H	1.268685000	4.881957000	-1.779030000
H	4.676929000	2.613779000	-0.262871000
S	-2.493488000	1.972283000	-0.299748000
O	-2.175331000	3.295439000	-0.892876000
O	-3.277180000	1.019701000	-1.129170000
C	-3.587601000	2.370582000	1.196766000
F	-3.996123000	1.230798000	1.796059000
F	-2.915078000	3.110278000	2.090962000
F	-4.669052000	3.049495000	0.785482000

1-H2O: E= -1921.184154

Ir	-0.240350000	-0.477086000	-0.103757000
O	-0.246599000	-1.112920000	2.161118000
Si	0.902198000	1.531574000	-0.092725000
O	2.529885000	0.925379000	-0.565502000
N	1.758297000	-1.235099000	-0.160380000
C	2.057618000	-2.557156000	0.005767000
H	1.179163000	-3.206345000	0.167638000
C	3.356146000	-3.049138000	-0.050663000
H	3.528089000	-4.126532000	0.092166000

C	4.435765000	-2.163730000	-0.305622000
C	4.127920000	-0.812529000	-0.486463000
H	4.903555000	-0.058773000	-0.683941000
C	2.788089000	-0.357722000	-0.408993000
C	5.853504000	-2.672066000	-0.372193000
H	5.954179000	-3.451405000	-1.157174000
H	6.144977000	-3.147449000	0.588671000
H	6.576433000	-1.862512000	-0.591633000
C	0.608419000	2.856553000	-1.484683000
C	-0.654029000	3.682468000	-1.153061000
H	-0.863899000	4.416608000	-1.964421000
H	-0.542548000	4.259566000	-0.210963000
H	-1.558637000	3.046191000	-1.048057000
C	0.414995000	2.113191000	-2.827720000
H	-0.486798000	1.469244000	-2.837588000
H	1.287490000	1.468082000	-3.063363000
H	0.306807000	2.849215000	-3.656708000
C	1.826396000	3.796204000	-1.654477000
H	1.679992000	4.436103000	-2.554857000
H	2.766324000	3.224776000	-1.800764000
H	1.968566000	4.476633000	-0.794813000
C	1.274697000	2.249367000	1.698207000
C	1.934959000	3.643920000	1.675275000
H	1.265947000	4.419420000	1.249196000
H	2.882830000	3.648841000	1.098514000
H	2.178621000	3.962913000	2.715316000
C	-0.037789000	2.324252000	2.511212000
H	0.164033000	2.755064000	3.518991000
H	-0.488416000	1.321976000	2.658570000
H	-0.793747000	2.979107000	2.026149000
C	2.253599000	1.282411000	2.405819000
H	2.416146000	1.608455000	3.458456000
H	3.243523000	1.256302000	1.906085000
H	1.874077000	0.241724000	2.446127000
C	-2.190936000	0.189858000	-0.949512000

C	-2.168720000	0.438135000	0.449718000
C	-3.002360000	-0.275320000	1.494692000
H	-2.529967000	-0.134796000	2.488666000
H	-3.001930000	-1.364686000	1.311030000
C	-4.449182000	0.276951000	1.536295000
H	-4.437958000	1.281186000	2.017625000
H	-5.051126000	-0.376271000	2.207696000
C	-5.156510000	0.408254000	0.175027000
H	-4.610697000	1.149600000	-0.451234000
H	-6.157548000	0.861633000	0.350803000
C	-4.094156000	-1.661333000	-1.075872000
H	-3.646419000	-2.233098000	-0.241791000
C	-2.993530000	-0.830941000	-1.760808000
H	-2.275988000	-1.531511000	-2.237822000
H	-3.448803000	-0.257814000	-2.602082000
H	-1.937280000	1.072578000	-1.560290000
H	-1.968319000	1.490582000	0.727575000
H	-0.658716000	-1.981398000	1.838339000
H	0.667273000	-1.363859000	2.403966000
H	-0.078395000	-0.325442000	-1.650742000
Cl	-1.162567000	-2.950084000	0.126212000
C	-5.355548000	-0.888011000	-0.633211000
H	-6.003094000	-1.582211000	-0.050172000
H	-5.943719000	-0.623641000	-1.542048000
H	-4.414635000	-2.438754000	-1.803993000

4-H2O: E= -2421.993398

Ir	0.297243000	-0.142179000	0.162131000
O	-0.368120000	0.421886000	-2.028628000
Si	2.516183000	-0.713786000	-0.013567000
O	3.243226000	0.857326000	0.450737000
N	1.130338000	1.805927000	0.207803000
C	0.369270000	2.936899000	0.117915000

H	-0.717189000	2.795741000	0.020337000
C	0.912923000	4.213780000	0.187723000
H	0.237647000	5.079400000	0.120242000
C	2.310485000	4.378530000	0.366950000
C	3.087982000	3.219143000	0.458088000
H	4.178685000	3.256980000	0.590338000
C	2.488703000	1.940068000	0.375769000
C	2.920060000	5.753791000	0.458150000
H	2.499293000	6.308152000	1.323959000
H	2.681845000	6.350261000	-0.447919000
H	4.020775000	5.716083000	0.572088000
C	3.328358000	-1.901160000	1.283726000
C	3.014690000	-3.368124000	0.912284000
H	3.453219000	-4.060934000	1.666029000
H	3.437465000	-3.649420000	-0.075422000
H	1.922966000	-3.572803000	0.879370000
C	2.759850000	-1.575768000	2.684670000
H	1.671853000	-1.771061000	2.764705000
H	2.929060000	-0.512629000	2.956456000
H	3.266700000	-2.203427000	3.452111000
C	4.861048000	-1.687508000	1.349175000
H	5.281528000	-2.276255000	2.196015000
H	5.119110000	-0.622309000	1.521690000
H	5.380625000	-2.022854000	0.432988000
C	3.123860000	-0.911540000	-1.868588000
C	4.576439000	-1.421395000	-1.980084000
H	4.696551000	-2.447232000	-1.575101000
H	5.298011000	-0.756970000	-1.461503000
H	4.875691000	-1.460307000	-3.052768000
C	2.186434000	-1.881900000	-2.620960000
H	2.534540000	-2.008831000	-3.671562000
H	1.146217000	-1.500947000	-2.653238000
H	2.174159000	-2.892802000	-2.159861000
C	3.059414000	0.478859000	-2.543435000
H	3.328548000	0.391041000	-3.620350000

H	3.763094000	1.199444000	-2.079262000
H	2.045313000	0.922579000	-2.501563000
C	-0.569380000	-2.008814000	1.016364000
C	-0.466109000	-2.133900000	-0.393934000
C	-1.623988000	-2.189914000	-1.369102000
H	-1.259485000	-1.925350000	-2.382988000
H	-2.388135000	-1.436803000	-1.110714000
C	-2.249608000	-3.608807000	-1.407107000
H	-1.571383000	-4.284303000	-1.976009000
H	-3.192315000	-3.555577000	-1.996841000
C	-2.531223000	-4.256485000	-0.038872000
H	-1.569469000	-4.402506000	0.503231000
H	-2.916225000	-5.284799000	-0.219720000
C	-3.203922000	-2.078847000	1.295769000
H	-3.371690000	-1.380670000	0.459542000
C	-1.809057000	-1.821901000	1.892809000
H	-1.815529000	-0.789272000	2.302187000
H	-1.663209000	-2.486015000	2.775124000
H	0.260487000	-2.479331000	1.570269000
H	0.397408000	-2.734132000	-0.736795000
H	-1.250301000	0.683704000	-1.621833000
H	0.054796000	1.274352000	-2.253968000
H	0.638061000	-0.191381000	1.685572000
S	-3.056661000	1.550340000	0.587052000
C	-4.441914000	1.339978000	-0.695407000
O	-1.978390000	0.665069000	-0.086742000
O	-3.562367000	0.993234000	1.859106000
O	-2.726074000	2.999382000	0.522974000
F	-3.987870000	1.725271000	-1.908378000
F	-4.835177000	0.052150000	-0.783854000
F	-5.499200000	2.093762000	-0.366202000
C	-3.528893000	-3.528262000	0.882674000
H	-4.531819000	-3.528163000	0.397969000
H	-3.642606000	-4.143955000	1.804461000
H	-3.942857000	-1.759004000	2.059952000

3b-H2O: E= -2766.300401

Ir	0.231564000	-0.020713000	0.019986000
P	0.041192000	-2.319457000	0.079660000
H	1.288297000	-3.011569000	0.211189000
Si	2.528115000	0.181307000	0.095599000
O	2.658660000	1.968360000	0.270948000
O	-0.473599000	0.320248000	-2.166581000
H	-1.465044000	0.540379000	-2.101410000
H	-0.052555000	1.129150000	-2.514392000
N	0.356407000	2.108417000	0.039664000
C	1.577517000	2.717215000	0.174547000
C	1.687323000	4.129324000	0.200735000
H	2.693569000	4.557013000	0.316422000
C	0.550090000	4.934396000	0.071957000
C	-0.698487000	4.280096000	-0.089751000
H	-1.633115000	4.848612000	-0.203572000
C	-0.749004000	2.891024000	-0.099972000
H	-1.694726000	2.354332000	-0.226656000
C	0.635129000	6.439649000	0.102444000
H	0.085328000	6.843296000	0.979600000
H	0.163222000	6.880949000	-0.800742000
H	1.682055000	6.796255000	0.158482000
C	3.588319000	-0.072190000	-1.520621000
C	4.843119000	0.830885000	-1.562127000
H	5.598100000	0.559009000	-0.801186000
H	4.583345000	1.899142000	-1.415777000
H	5.334605000	0.734188000	-2.557637000
C	4.004998000	-1.552657000	-1.647907000
H	3.138674000	-2.240619000	-1.562118000
H	4.742918000	-1.842233000	-0.870703000
H	4.479697000	-1.743398000	-2.637805000
C	2.693636000	0.327973000	-2.715405000
H	3.265589000	0.254662000	-3.668669000

H	2.348576000	1.382284000	-2.622541000
H	1.802634000	-0.319857000	-2.806554000
C	3.423144000	-0.366979000	1.740839000
C	2.794606000	0.414920000	2.917416000
H	1.706386000	0.218699000	3.017531000
H	2.926192000	1.509792000	2.797369000
H	3.277740000	0.115369000	3.875263000
C	4.930534000	-0.032336000	1.697018000
H	5.110539000	1.040108000	1.476195000
H	5.475528000	-0.637797000	0.944252000
H	5.394334000	-0.251995000	2.686064000
C	3.244963000	-1.879258000	1.990871000
H	3.793630000	-2.184610000	2.911421000
H	3.631769000	-2.500952000	1.155959000
H	2.179883000	-2.143462000	2.145530000
C	-0.534723000	-3.209003000	-1.519021000
C	0.508862000	-2.834517000	-2.592850000
H	0.223372000	-3.310765000	-3.556041000
H	1.526597000	-3.193786000	-2.335537000
H	0.540741000	-1.740376000	-2.751970000
C	-0.534243000	-4.740646000	-1.343495000
H	-1.308465000	-5.089614000	-0.633390000
H	0.452065000	-5.121984000	-1.005439000
H	-0.754429000	-5.213286000	-2.325678000
C	-1.917535000	-2.715161000	-1.981150000
H	-2.183920000	-3.233918000	-2.927725000
H	-1.910924000	-1.632600000	-2.198206000
H	-2.725938000	-2.903348000	-1.254086000
C	-0.859216000	-3.044667000	1.636301000
C	-0.151209000	-4.348948000	2.065502000
H	0.912656000	-4.174067000	2.330601000
H	-0.186445000	-5.135517000	1.288011000
H	-0.655823000	-4.755143000	2.969486000
C	-2.354184000	-3.302902000	1.361142000
H	-2.833283000	-3.638512000	2.306697000

H	-2.524504000	-4.101332000	0.612919000
H	-2.881410000	-2.391113000	1.024424000
C	-0.761812000	-2.023093000	2.791724000
H	-1.293026000	-1.083565000	2.544907000
H	0.287430000	-1.778442000	3.054421000
H	-1.235668000	-2.466540000	3.694829000
H	0.510337000	-0.095854000	1.550390000
S	-3.357562000	0.135068000	-0.401368000
F	-5.625131000	1.529873000	-0.218256000
F	-3.868540000	2.644835000	0.450414000
F	-4.701265000	1.062050000	1.708435000
O	-2.143039000	0.075021000	0.517984000
O	-4.206068000	-1.076801000	-0.435198000
O	-3.015036000	0.766654000	-1.735220000
C	-4.462700000	1.430676000	0.441325000
